# Liver fat metabolism of broilers regulated by *Bacillus amyloliquefaciens* TL *via* stimulating IGF-1 secretion and regulating the IGF signaling pathway

**DOI:** 10.3389/fmicb.2022.958112

**Published:** 2022-07-27

**Authors:** Pinpin Chen, Shijie Li, Zutao Zhou, Xu Wang, Deshi Shi, Zili Li, Xiaowen Li, Yuncai Xiao

**Affiliations:** State Key Laboratory of Agricultural Microbiology, College of Veterinary Medicine, Huazhong Agricultural University, Wuhan, China

**Keywords:** *Bacillus amyloliquefaciens* TL, broilers, lipid metabolism, liver, insulin-like factor 1, IGF signaling pathway, fatty acid synthase

## Abstract

*Bacillus amyloliquefaciens* TL (B.A-TL) is well-known for its capability of promoting protein synthesis and lipid metabolism, in particular, the abdominal fat deposition in broilers. However, the underlying molecular mechanism remains unclear. In our study, the regulations of lipid metabolism of broilers by B.A-TL were explored both *in vivo* and *in vitro*. The metabolites of B.A-TL were used to simulate *in vitro* the effect of B.A-TL on liver metabolism based on the chicken hepatocellular carcinoma cell line (i.e., LMH cells). The effects of B.A-TL on lipid metabolism by regulating insulin/IGF signaling pathways were investigated by applying the signal pathway inhibitors *in vitro*. The results showed that the B.A-TL metabolites enhanced hepatic lipid synthesis and stimulated the secretion of IGF-1. The liver transcriptome analysis revealed the significantly upregulated expressions of four genes (*SI, AMY2A, PCK1*, and *FASN*) in the B.A-TL treatment group, mainly involved in carbohydrate digestion and absorption as well as biomacromolecule metabolism, with a particularly prominent effect on fatty acid synthase (FASN). Results of cellular assays showed that B.A-TL metabolites were involved in the insulin/IGF signaling pathway, regulating the expressions of lipid metabolism genes (e.g., *FASN, ACC*α*, LPIN*, and *ACOX*) and the FASN protein, ultimately regulating the lipid metabolism *via* the IGF/PI3K/FASN pathway in broilers.

## Graphical Abstract

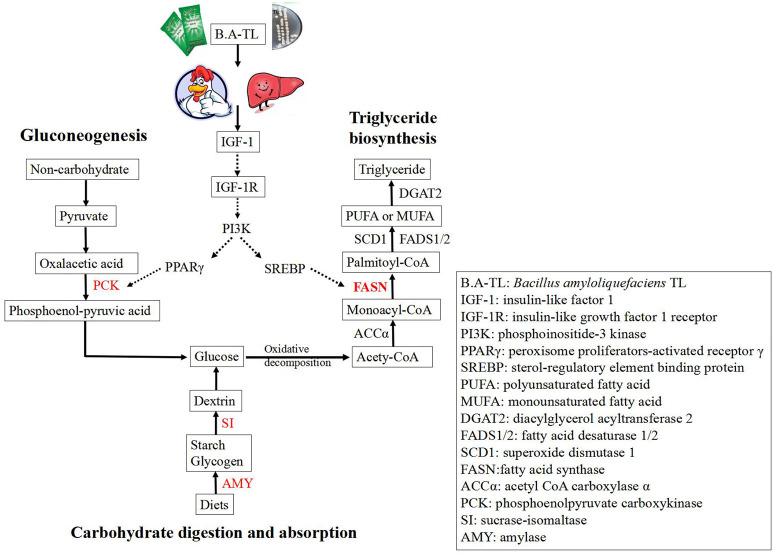

## Introduction

More than 60 years ago, Salmon and Daughaday (1957) first described a serum factor that was controlled by hormones to stimulate sulfate incorporation by cartilage *in vitro*. This serum factor was later identified as the insulin-like growth factor (IGF) (Daughaday et al., 1987). The IGF system consists of two ligands, i.e., IGF-1 and IGF-2 (Moerth et al., 2007; Poreba and Durzynska, 2020). The IGF-1 is a pleiotropic factor, expressed mainly in the liver (Poreba and Durzynska, 2020), and its physiological effects are to promote tissue growth and development and to regulate both lipid and carbohydrate metabolisms as well as the anti-inflammatory, antioxidant, and neuro- and hepato-protective properties (Liu et al., 2016; Halmos and Suba, 2019; Stanley et al., 2021). The IGF-2 is expressed in many tissues, in particular the placenta (Forbes et al., 2020). The IGF-2 plays an important role in follicular growth and in oocyte and embryonic development (Sirotkin et al., 2000; Chang et al., 2021), generally considered as a major growth factor involved in the early body development (Chang et al., 2021).

The growth hormone (GH)/IGF system, also known as the pituitary-liver axis, has shown somatotrophic effects *in vivo* (Li, 2022). However, both animal and clinical studies have revealed that IGF-2 is not a substitute for IGF-1 to stimulate normal postnatal body growth (Sjögren et al., 1999; Moerth et al., 2007), while animal weight growth is regulated by GH and IGF-1 (Sjögren et al., 1999; Anh et al., 2015; Jia et al., 2018). Although the effect of GH can be directly activated by tyrosine kinase *via* the GH receptor, more peripheral effects are mainly mediated by stimulating the expression of IGF-1 in the liver and peripheral tissues (Sjögren et al., 1999; Brooks and Waters, 2010; Sigalos and Pastuszak, 2018). Serum IGF-1 level is an indicator for GH level simply because IGF-1 is both a downstream effector and an upstream regulator of GH (Sigalos and Pastuszak, 2018). Several studies have shown that IGF-1 can stimulate physical growth in animal models with GH deficiency/GH receptor mutation (Sjögren et al., 1999; Laron, 2008; Castilla-Cortazar et al., 2017). In a study on LI-IGF-1/- mice with the *IGF-1* gene knocked out by the Cre/loxP recombinant system, Sjögren et al. (1999) reported that the liver-derived IGF-1 exerted a negative feedback regulation on the pulsatile secretion pattern of GH. Furthermore, studies showed that the Benha line chickens with improved growth performance, carcass characteristics, and meat quality traits showed the highest levels of hepatic *GH* and *IGF-1* as well as the muscle *IGF-1* gene expression compared to both Rhode Island Red and Golden Montazah chickens (El-Attrouny et al., 2021). Moreover, as the two major components in the somatotropic axis, the GH is the main regulator of growth rate, while the IGF-1 regulates both growth rate and body weight in broilers (Jia et al., 2018).

It is well known that the binding of IGF to its receptor leads to the activation of the insulin (INS)/IGF signaling cascade pathway, which contains two major downstream signaling pathways, including (1) the IRS-initiated PI3K-AKT/rapamycin pathway primarily involved in metabolism and (2) the SHC-initiated RAS/MAPK pathway controlling mitosis (Forbes et al., 2020). At the cellular level, the INS/IGF signaling pathway has been considered to contribute to cell division and glucose (GLU) metabolism (Perry and Shulman, 2020), whereas the current understanding of insulin signaling in chickens is rather inadequate compared to that in mammals (Dupont et al., 2009). Despite the lack of convincing evidence, it appears that the insulin signaling in the chicken liver is similar to that in mammals but different from that in chicken muscle (Dupont et al., 2009). Furthermore, it has been reported that the activation of insulin signaling in the liver during the early developmental stages of broiler chickens could cause the acceleration of lipogenesis, ultimately fattening fatty broilers (Dupont et al., 1999).

Previous studies revealed significantly increased body weight in broilers fed with *Bacillus amyloliquefaciens* TL (B.A-TL) in 21 days as well as improved feed efficiency and enhanced stress resistance of broilers (Hong et al., 2019). In particular, the results of the analysis of the cecum microbiota showed that the relative abundance of *Firmicutes* was increased, whereas the relative abundance of *Bacteroidetes* was decreased in the B.A-TL group (Hong et al., 2019). Furthermore, studies showed that a higher *Firmicutes/Bacteroidetes* ratio promoted not only body growth (Mancabelli et al., 2016; Salaheen et al., 2017) but also fat deposition (Ley et al., 2005; Hong et al., 2019) in various types of animal models. Moreover, the ileal transcriptome analysis showed that the treatment of B.A-TL reduced the levels of both intestinal inflammation and intestinal damage and facilitated the energy accumulation in broilers, with the upregulated differentially expressed genes (DEGs) significantly enriched in the “insulin signaling pathway” and both genes *FGF16* and *FGF10* expressed only in the B.A-TL group (Hong et al., 2021). This unique expression of the FGF family members in the B.A-TL group suggested enhanced glycogen and protein syntheses and lipid differentiation in the B.A-TL group (Hong et al., 2021).

In avian species, lipogenesis takes place primarily in the liver, accounting for 95% of *de novo* fatty acid (FA) synthesis (Han et al., 2015), while the gut-derived products exit the gut and enter the portal circulation to bath the liver and potentially regulate the hepatic metabolism and function (Kieffer et al., 2016; Arab et al., 2018; Albillos et al., 2020). Therefore, in this study, we performed liver transcriptome analysis, liver oil red O staining, and serum analysis of broilers at 21 days of age to elucidate the lipid metabolism changes in response to the probiotic treatment of B.A-TL. Since B.A-TL cannot but its metabolites can reach liver tissues through the gut-liver axis, the B.A-TL metabolites were used in this study to act on LMH cells to simulate the effect of B.A-TL entering broiler chickens on the liver. Furthermore, the quantitative real-time polymerase chain reaction (qRT-PCR) analysis and western blotting techniques as well as the cellular pathway blocking assays were performed to investigate the regulatory mechanism of B.A-TL underlying the INS/IGF signaling pathway and thus regulating the lipid metabolism.

## Materials and methods

### Bacterial strains and antibodies

As a facultative anaerobic bacterium closely related to *Bacillus subtilis* (Hong et al., 2019), the B.A-TL is an invention of a patented strain (Patent No. ZL 2015 1 0551645.9) obtained from the Hubei Huada Real Technology Co., Ltd. (Wuhan, China). Both the horseradish peroxidase (HRP)-conjugated goat anti-rabbit IgG and the rabbit β-Actin mAb antibody were purchased from ABclonal (Wuhan, China). Rabbit mAbs against FASN were purchased from the Hangzhou HuaAn Biotechnology Co., Ltd. (Hangzhou, China).

### Extraction and analysis of metabolites of fermentation supernatant and exopolysaccharides of B.A-TL

The metabolomics analysis of B.A-TL fermentation supernatant (FST) was performed by liquid chromatography with tandem mass spectrometry (LC-MS/MS). The B.A-TL was transferred from petri dishes to LB medium and incubated at 37°C for 12 h. Then, a 5 mL seed medium was inoculated into a 500 mL sterile seed medium and cultured at 37°C and 180 rpm for 24 h to generate the FST.

A total of 5 mL seed medium was inoculated into a 500 mL sterile seed medium, cultured at 37°C and 180 rpm for 72 h. After fermentation, the broth was centrifuged at 8,000 rpm for 15 min to extract exopolysaccharides (EPS) for 12 h at 4°C under various pH values with 60–90% ethanol. The precipitate was collected by filtration and then dissolved in distilled water. After the removal of proteins by Sevag reagent, the polysaccharide was dialyzed using a dialysis bag (molecular weight cutoff = 7 kDa; Beijing Solarbio Technology Co., Ltd., Beijing, China) extensively against distilled water for 72 h and then lyophilized.

The FST sample (20 mL) was frozen into powder, dissolved with 10 mL of methanol solution, shaken to mix for 1 min, then centrifuged at 12,000 rpm and 4°C for 10 min, and with the supernatant transferred to a new centrifuge tube. Samples were vacuum dried to concentrate, then dissolved with 2-chlorobenzalanine (4 ppm) and 80% methanol solution and filtered through 0.22 μm membrane for the liquid chromatography and mass spectrometry (LC-MS) analysis (UltiMate 3000 and Q Exactive Focus, Thermo Fisher, Waltham, MA, USA). A total of 20 μL of each sample was used as the quality control, while the rest of each sample was used for LC-MS analysis.

The EPS sample of 5 mg (±0.05 mg) was added to a clean chromatography bottle with configured TFA acid solution, heated for 2 h at 121°C, and blow-dried with nitrogen. Then, the sample was cleaned with methanol, blow-dried again, and repeated twice. Finally, the sample was dissolved in aseptic water and transferred to the chromatography bottle for testing using the Thermo ICS5000 plus ion chromatography system (Thermo Fisher, Waltham, MA, USA) and the DynexTM CarboPacTM PA10 liquid chromatography column (250 × 4.0 mm, 10 μm) with a sample size of 5 μL, flow phase A (H_2_O), and flow phase B (100 mM NaOH).

### Laboratory animals

The present study was performed in strict accordance with the Guide for the Care and Use of Laboratory Animals Monitoring Committee of Hubei Province, China, with the protocol approved by the Committee on the Ethics of Animal Experiments of the College of Veterinary Medicine, Huazhong Agricultural University (approval No. HZAUCH-2020-0009). All chickens were reared in cages. A total of 108 3-day-old male Cobb broiler chickens were randomly and evenly divided into two groups, i.e., the control and the B.A-TL treatment groups, with six pens (9 chickens per pen) in each group. Chickens in the control group were fed with a basal diet (no drugs or additives added) and the B.A-TL group was fed with grain containing B.A-TL (4 × 10^6^ CFU/g) for 21 days. The B.A-TL was added to the feed in the form of dry powder at a concentration of 200 g/ton and an effective viable number of 2 × 10^10^ CFU/g (Hong et al., 2019, 2021), as recommended by the manufacturer's instructions. Our previous studies have shown that the probiotics of B.A-TL were the most effective in promoting growth in broilers over a 21-day growth period (Hong et al., 2019, 2021).

The chicken coop, where the pens were housed, and the surrounding areas were thoroughly disinfected before the trial started, with the temperature of the chicken coop maintained at ~33°C until the chickens were 7 days old. Then, the temperature was gradually reduced to and finally maintained at 23°C. All chickens were free to feed and drink and were weighed once a week with no antibiotics used during the entire experiment. Feed consumption by chickens in both groups was recorded daily. The broiler pellet feed formulation and nutrient compositions are provided in Supplementary Table 1.

### Tissue sampling

A total of six chickens were sampled from each of the control and the B.A-TL groups on day 21, i.e., for a total of 12 chickens, with the pen number and weight of each individual chicken recorded before slaughter. The most representative chicken was selected based on the median weight of each pen for the liver transcriptomic analysis and the representative data were applied to predict the molecular patterns in the entire population (Supplementary Table 2). Blood samples (~3 mL in each of the 2 tubes for each chicken) were taken from the wing vein, collected into the vacuum blood collection tubes, and centrifuged at 3,000 rpm and 4°C to obtain the serum sample. The chickens were euthanized with the liver tissues collected immediately for further analyses. One-half of the liver tissue was washed with phosphate buffer solution (PBS 0.01M pH 7.4; Biosharp Co., Ltd., Hefei, China), snap-frozen in liquid nitrogen, and stored at −80°C for further mRNA expression analysis. Simultaneously, the other half of the liver tissue was immediately fixed in 4% paraformaldehyde (Biosharp Co., Ltd., Hefei, China) for subsequent morphological analysis.

### Serum analysis

The contents of glucose (GLU), triglycerides (TG), cholesterol (CHO), alanine aminotransferase (ALT), and aspartate aminotransferase (AST) were measured by a BK-280 automatic biochemical analyzer (Shandong Blobase Biotechnology Co., Ltd., Shandong, China). The contents of INS, GH, IGF-1, very low-density lipoprotein (VLDL), malondialdehyde (MDA), catalase (CAT), superoxide dismutase (SOD), total antioxidant capacity (T-AOC), and glutathione peroxidase (GSH-Px) were measured using the enzyme-linked immunosorbent assay (ELISA) kits (Wuhan Meimian Biotechnology Co., Ltd., Wuhan, China). All experiments were performed in strict accordance with the protocols and instructions recommended by the manufacturers.

### Hematoxylin and eosin staining and oil red O staining

Liver samples were embedded in paraffin, sectioned (3-μm thickness), stained with hematoxylin and eosin (HE) for panoramic scanning, and imaged with a Panoramic MIDI slide scanner (3D HISTECH Co., Ltd., Budapest, Hungary). According to a previous protocol (Pinterić et al., 2020), 0.5% Oil Red O solution (Sigma Aldrich, St. Louis, MO, USA) was prepared. Tissues were embedded in Optimal Cutting Temperature medium (CRYOSTAR NX50, Thermo Fisher Scientific, Waltham, MA, USA), sectioned (8-μm thickness), air-dried for 1 h, and fixed in 10% formaldehyde for 5 min. The LMH cells (70–80% confluent) cultured on the six-well plates were stimulated by FST (10^−4^), EPS (0.1 mg/mL), and L-Tyr (0.25 mmol/L) for 4 h, respectively, and washed thrice with PBS, and fixed in 10% formaldehyde for 30 min. All samples were briefly stained with 60% isopropanol, Oil Red O dye for 15 min, rinsed with 60% isopropanol, and incubated in ddH_2_O for 5 min, then stained with Mayer hematoxylin (Wuhan Baichan Biotechnology Co., Ltd., Wuhan, China) for 1 min, washed with tap water and ddH_2_O, and finally mounted in aqueous mounting solution (Wuhan Baichan Biotechnology Co., Ltd., Wuhan, China). Images were obtained using an Axiovert 40 CFL microscope (Olympus, Tokyo, Japan) and analyzed using Image-Pro plus 6.0 (Media Cybernetics, Rockville, MD, USA). All experiments were performed in triplicates.

### RNA extraction, library preparation, and sequencing

Total RNA was extracted from the liver tissue using RNApure Tissue and Cell Kit according to the manufacturer's instructions (Cowin Biotech Co., Ltd., Taizhou, China). RNA concentration and quality were determined by NanoDrop 2000c (Thermo Fisher, Waltham, MA, USA). RNA samples (each of 1 μg) with high quality (i.e., based on OD_260/280_ ratio = 1.8~2.2, OD_260/230_ ratio ≥ 2.0, RIN ≥ 6.5, 28S:18S ratio ≥ 1.0, and weighed more than 1 μg) were submitted to Shanghai Majorbio Bio-pharm Technology Co., Ltd. (Shanghai, China) to construct RNA-Seq transcriptomic library using the TruSeq™ RNA sample preparation Kit from Illumina (San Diego, CA, USA). The mRNAs were isolated according to the polyA selection method by oligo(dT) beads and then fragmented by fragmentation buffer. Then, the double-stranded cDNA was synthesized using the SuperScript double-stranded cDNA synthesis kit (Invitrogen, CA, USA) with random hexamer primers (Illumina, CA, USA). The synthesized cDNA was subjected to end-repair, phosphorylation, and “A” base addition according to Illumina's library construction protocol. Libraries were size selected for cDNA target fragments of 300 bp on 2% low-range ultra agarose followed by PCR amplification of 15 cycles using Phusion DNA polymerase (NEB, Beijing, China). After quantified by TBS380, the paired-end RNA-Seq library was sequenced with the Illumina HiSeq xten/NovaSeq 6000 sequencer (with 2 × 150 bp read length).

### RNA-Seq read mapping

The raw paired-end reads were trimmed, and quality controlled by SeqPrep and Sickle with default parameters. The clean reads of control and experimental groups were separately aligned to the reference genome with orientation mode using HISAT2 (Kim et al., 2015). The mapped reads of each sample were assembled by StringTie using a reference-based approach (Pertea et al., 2015).

### Differential expression analysis and functional enrichment analysis

To identify the DEGs between two different samples, the expression level of each transcript was calculated according to the transcripts per million reads (TPM) method. RSEM (Li and Dewey, 2011) was used to quantify gene abundances. The differential expression analysis was performed using the DESeq2 (Love et al., 2014) with Q value ≤ 0.05. DEGs were identified based on fold change > 2 or < −2 and Q value ≤ 0.05. Functional enrichment analysis based on Gene Ontology (GO) and Kyoto Encyclopedia of Genes and Genomes (KEGG) databases were performed by Goatools and KOBAS, respectively (Xie et al., 2011), to identify the DEGs significantly enriched in GO terms and KEGG metabolic pathways based on the Bonferroni-corrected *p*-value ≤ 0.05 compared with the background of the entire transcriptome.

### Quantitative real-time polymerase chain reaction analysis

In the validation experiments based on qRT-PCR analysis, a total of 12 genes were randomly selected to assess the reliability of RNA-Seq data (Supplementary Table 3). The RNA sample of 1 μg was reverse-transcribed into cDNA using the PrimeScript™ RT Reagent Kit with gDNA Eraser (TaKaRa, Dalian, China) according to the manufacturer's instructions. The cDNA was diluted 10-fold and used for qRT-PCR analyses with a Bio-Rad CFX96TM System and signal detection protocols in accordance with the manufacturer's instructions (TaKaRa, Dalian, China). The qRT-PCR experiments were performed in three replicates with the β*-Actin* used as the endogenous control and the expression of individual genes normalized to that of β*-Actin* (Xiang et al., 2017). Primers used for qRT-PCR experiments are shown in Supplementary Table 3.

### CCK8 cell viability and proliferation assays

The LMH cells (stored in the Microbiology and Immunology Laboratory of Huazhong Agricultural University, Wuhan, China) were cultured in Dulbecco's Modified Eagle Medium/Nutrient Mixture F-12 (DMEM/F12; Gibco, Waltham, MA, USA) supplemented with 10% fetal bovine serum (FBS; Gibco, Waltham, MA, USA). A total of 100 μL of the diluted cell culture was added into each well of a 96-well cell culture cluster (Biosharp Co., Ltd., Hefei, China), cultured for 12 h in DMEM/F12 at 37°C to achieve 70% of the cells of the full monolayer and then starved for 12 h in DMEM/F12 without FBS at 37°C. The cell cultures were washed with 100 μL PBS before the treatment with 100 μL DMEM/F12 containing FST (10^−3^, 10^−4^, 10^−5^, and 10^−6^, respectively), EPS (0.01, 0.1, 1, and 5 mg/mL, respectively), and L-Tyr (0.1, 0.25, 0.5, 0.75, and 1 mmol/L, respectively) for 2, 4, 8, and 12 h, respectively, while the control was treated with 100 μL DMEM/F12. Finally, the 10 μL Cell Counting Kit-8 (CCK8, Dojindo Molecular Technologies, Inc., USA) was added to the cell cultures and incubated for 1 h. The OD at 450 nm of the cultures was measured using a MULTISKAN MK3 (Thermo Electron Corporation, Waltham, MA, USA).

### Cell RNA preparation and qRT-PCR analysis

The LMH cells (70–80% confluence) were stimulated with FST (10^−4^), EPS (0.1 mg/mL), and L-Tyr (0.25 mmol/L), respectively. Total RNA was extracted using the Ultrapure RNA Kit (CW0581M, CoWin Biosciences, Beijing, China) and the reverse transcription reaction was performed using PrimeScript™ RT Reagent Kit with gDNA Eraser (TaKaRa, Dalian, China) following the manufacturer's protocol. After mixing the extracted RNA with ChamQ Universal SYBR qPCR Master Mix (Q711-02/03, Vazyme Biotech Co., Ltd., Nanjing, China) and specific primers, the qRT-PCR analyses were performed on a CFX96TM (Bio-Rad, Richmond, CA, USA). Primers used for the qRT-PCR analysis (Han et al., 2015) are shown in Supplementary Table 4.

### Western blotting

The LMH cells (70–80% confluence) were stimulated with FST (10^−4^), EPS (0.1 mg/mL), and L-Tyr (0.25 mmol/L), respectively. Cells were washed twice with PBS and incubated on ice with RIPA Lysis Buffer (Beyotime, Shanghai, China) containing a protease inhibitor cocktail (Roche, Basel, Switzerland). The cell lysates were separated with sodium dodecyl sulfate-polyacrylamide gel electrophoresis (SDS-PAGE) of 7.5% gel. The proteins were then transfer-embedded onto a polyvinylidene difluoride membrane (Bio-Rad, Richmond, CA, USA), which was blocked with TBST containing 5% skim milk (BD, San Jose, CA, USA) for 1 h and then incubated with the primary antibody overnight at 4°C, followed by the incubation with corresponding HRP-conjugated secondary antibody for 1 h. The antibodies used in the western blotting experiments included anti-FASN (1:1,000) and anti-β-Actin (1:10,000). β-Actin was employed as the loading standard. The protein bands were quantified with the ImageJ software. All western blotting analyses were performed as previously described (Guo et al., 2016).

### Signaling pathway inhibition assays

The LMH cells (70–80% confluence) were treated with pathway inhibitor picropodophyllin (2.5 μM; MedChemexpress, NJ, USA) for 1 h and then either LY294002 (10 nM; MedChemexpress, NJ, USA) or PD98059 (10 nM; MedChemexpress, NJ, USA) for 24 h before being stimulated with FST (10^−4^), EPS (0.1 mg/mL), and L-Tyr (0.25 mmol/L), respectively. The cells were harvested at the indicated time points (4 h) for RNA and protein extractions. The qRT-PCR was performed to detect the expression of specified genes (i.e., *FASN, ACC*α, *SREBP1, SCD1, LXR*α, *LPIN1, CPT1A, PPAR*α, and *ACOX1*) (Han et al., 2015). Western blotting assays were performed to evaluate the expression of specified proteins (i.e., β-Actin and FASN).

### Statistical analysis

Significant differences were determined with Student's *t*-test using GraphPad Prism 8.0 (GraphPad Software, Inc., San Diego, CA, USA). Unless indicated, data were shown as mean ± standard error of the mean (SEM). The significance levels for all statistical analyses were set to *p* < 0.05 (^*^), *p* < 0.01 (^**^), and *p* < 0.001 (^***^), respectively.

## Results

### Analysis of FST and EPS

The results of the metabolomics studies of B.A-TL FST showed that a total of 312 types of metabolites (Supplementary Table 5) were obtained and annotated in the samples with L-Tyr showing the highest content (29.02%) among all the identified components (supplementary figure 1C). Based on these results, L-Tyr was selected as the stimulant used in the following tests.

The EPS were acidified into monosaccharides with the monosaccharide compositions detected with an electrochemical detector (Supplementary Figure 2). The results showed that the EPS of B.A-TL contained mainly six types of sugars (Supplementary Table 6) with mannose, GLU, and galactose together accounting for 98.27% of the total EPS (Supplementary Figure 2B).

### Growth performance

The average body weight (ABW), average daily gain (ADG) and overall feed conversion ratio (FCR) in broilers are shown in Table 1. The ABWs and ADGs of the B.A-TL group were significantly higher than those of the control group at 7, 14, and 21 days with the most significant difference in ABW observed at 21 days (*p* < 0.001), while the overall FCR (*p* < 0.01) of broilers in the B.A-TL group was lower than that in the control group.

**Table 1:** Effects of *Bacillus amyloliquefaciens* TL on the growth performance of broilers in the control and experimental groups.

**Item**	**Time**	**Control group**	**B.A-TL group**	***p*** **-value**
Average body weight (g)	Initial	63.17 ± 0.09	63.20 ± 0.12	0.8050
	Week 1	175.40 ± 2.85^b^	185.30 ± 1.87^a^	0.0128
	Week 2	333.10 ± 6.62^b^	363.50 ± 6.57^a^	0.0117
	Week 3	696.80 ± 16.24^B^	787.10 ± 15.29^A^	<0.001
Average daily gain (g)	Week 1	15.78 ± 0.43^b^	17.12 ± 0.40^a^	0.0385
	Week 2	22.79 ± 0.61^B^	25.79 ± 0.62^A^	0.0040
	Week 3	51.94 ± 2.48^b^	60.51 ± 2.46^a^	0.0280
Feed conversion ratio	Overall	1.84 ± 0.02^A^	1.70 ± 0.03^B^	0.0035

*Data are expressed as mean ± SEM (n = 54 chickens per group). Means within a row with different superscripts differ significantly at p < 0.01 (A or B) and p < 0.05 (a or b), respectively*.

### Serum analysis

The results of the serum biochemical indices of Cobb broiler chickens treated with B.A-TL are shown in Table 2. No significant changes were observed in the contents of INS, GH, CHO, VLDL, AST, and GSH-Px between the B.A-TL and the control groups, while the other eight indices showed significant differences between the control and experimental groups. For example, in the B.A-TL group, the contents of GLU, IGF-1, and TG were increased by 35.92 (*p* < 0.001), 15.51 (*p* < 0.05), and 31.19% (*p* < 0.01), respectively, while the content of MDA was decreased by 63.15%, compared with those of the control group.

**Table 2:** Effects of *Bacillus amyloliquefaciens* TL on the levels of serum biochemical indices in broilers at 21 days of age in both the control and experimental groups.

**Biochemical index**	**Control group**	**B.A-TL group**	***p*** **-value**
INS (mL/L)	5.14 ± 0.24	5.58 ± 0.30	0.2830
GH (μg/L)	10.28 ± 0.20	10.04 ± 0.30	0.5260
IGF-1 (μg/L)	23.41 ± 0.35^b^	27.04 ± 1.36^a^	0.0275
GLU (mmol/L)	13.00 ± 0.55^B^	17.67 ± 1.19^A^	0.0051
TG (mmol/L)	0.37 ± 0.01^b^	0.50 ± 0.052^a^	0.0175
CHO (mmol/L)	4.09 ± 0.13	4.15 ± 0.27	0.9090
VLDL (mmol/L)	9.86 ± 1.73	9.94 ± 1.36	0.9725
ALT (U/L)	7.22 ± 0.48^b^	13.22 ± 2.14^a^	0.0211
AST (U/L)	341.10 ± 27.17	290.90 ± 3.98	0.1312
MDA (nmol/mL)	63.75 ± 9.16^A^	23.50 ± 6.64^B^	0.0081
CAT (U/mL)	1.72 ± 0.39^b^	3.92 ± 0.67^a^	0.0159
SOD (U/mL)	19.74 ± 0.14^B^	20.89 ± 0.17^A^	0.0004
T-AOC (U/mL)	0.16 ± 0.07^B^	0.59 ± 0.08^A^	0.0021
GSH-Px (μmol/L)	269.50 ± 29.20	277.80 ± 25.86	0.8360

*Data are expressed as mean ± SEM (n = 6 chickens per group). Means within a row with different superscripts differ significantly at p < 0.01 (A or B) and p < 0.05 (a or b), respectively*.

### HE staining and oil red O staining

**Figure 1:**
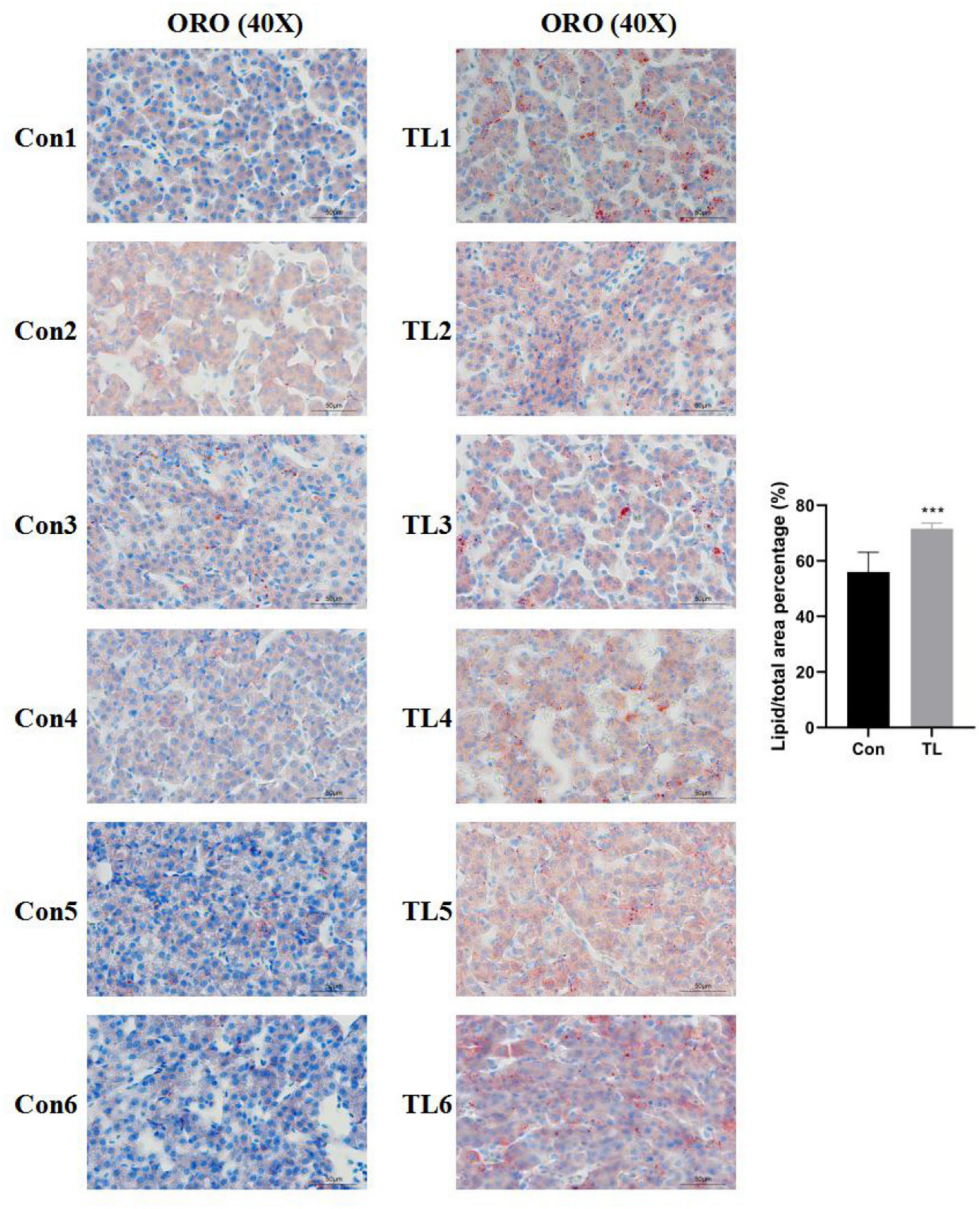
The liver tissues stained with Oil Red O of broilers in the control and experimental groups at 21 days of age. “Con” = control group; “TL” = Bacillus amyloliquefaciens TL experimental group. The significance level for all analyses is set to *p* < 0.001 (***).

**Figure 2:**
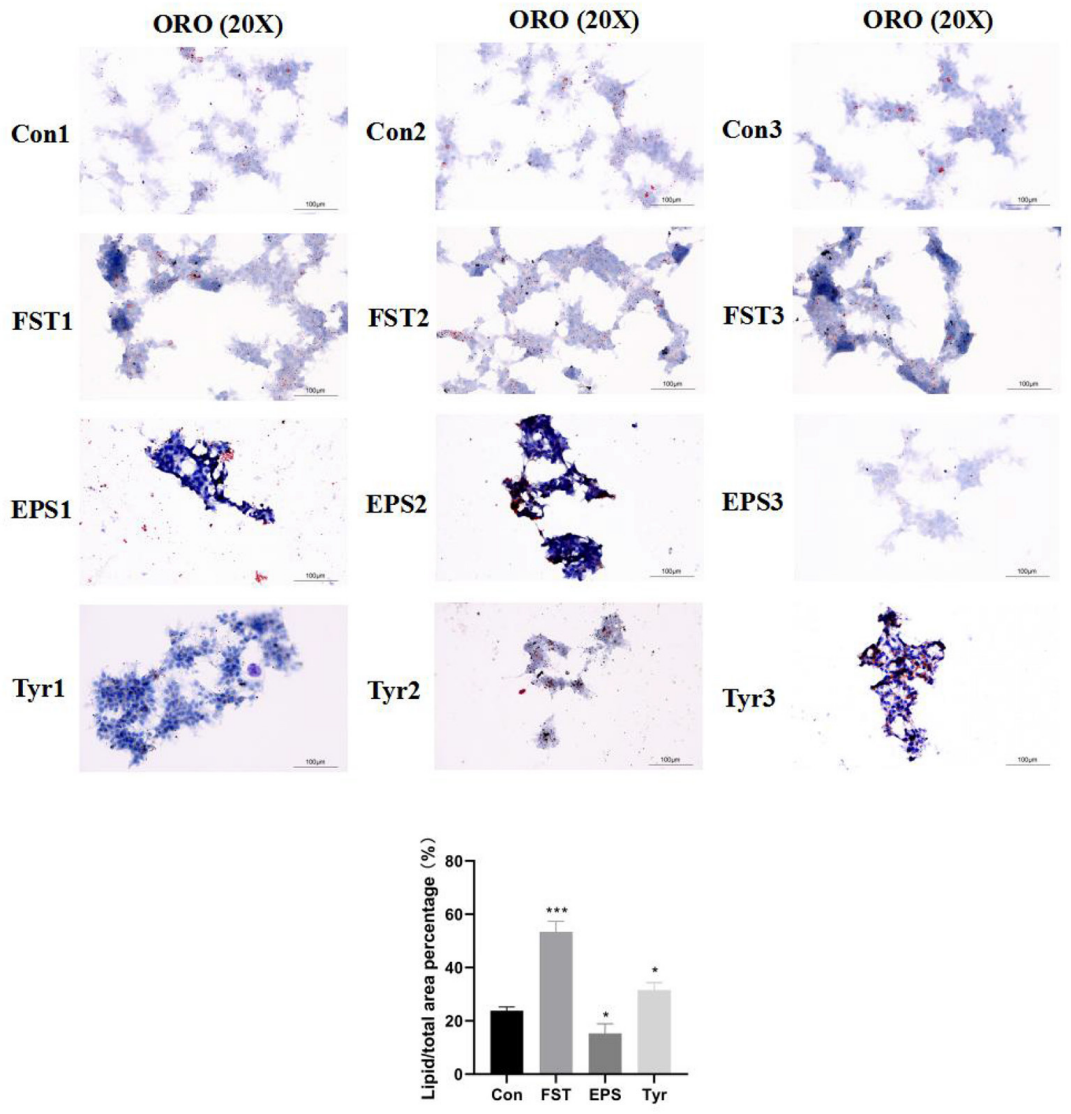
The Oil Red O staining of the LMH cells treated with Bacillus amyloliquefaciens TL fermentation supernatant, exopolysaccharide, and L-tyrosine, respectively. “Con” = control group; “FST” = fermentation supernatant group; “EPS” = exopolysaccharides group; “Tyr” = L-tyrosine group. The significance levels for all analyses were set at *p* < 0.05 (*), and *p* < 0.001 (***), respectively.

The observations of HE-stained sections showed that the liver tissue was not damaged by B.A-TL (Supplementary Figure 3). The results of the liver Oil Red O staining showed that the percentage of lipid droplets in the liver of broilers in the B.A-TL group was significantly higher than that in the control group (*p* < 0.001; Figure 1), indicating that B.A-TL accelerated the liver fat metabolism and promoted fat deposition in the liver tissue of the broiler chickens. The results of cellular Oil Red O staining (Figure 2) showed a significant increase in the percentage of lipid droplets in the FST (*p* < 0.001) and Tyr (*p* < 0.05) groups and a significant decrease in the EPS (*p* < 0.05) group, indicating that some metabolites of B.A-TL (i.e., FST and Tyr) could enhance the lipid synthesis activity of LMH cells and promote lipid droplet formation.

### Transcriptome analysis

The results of transcriptomics analysis based on the 11 liver samples (6 in control and 5 in experimental groups; the sample TL6 was removed due to its significant deviation from other samples) showed that after the removal of low-quality data, a total of 6.83–10.38 Gb of clean data were obtained for further analyses (Supplementary Table 7). The high Pearson correlation coefficients (*R*^2^) of the biological repeats in the control and B.A-TL groups (Supplementary Figure 4A) indicated that the RNA-Seq data were suitable for subsequent analyses. The transcriptome profiles of the liver tissues from the B.A-TL group were clearly separated from those of the control group based on the principal component analysis (PCA; Supplementary Figure 4B).

### Functional annotations of differentially expressed genes identified in the liver tissue of broilers

**Figure 3:**
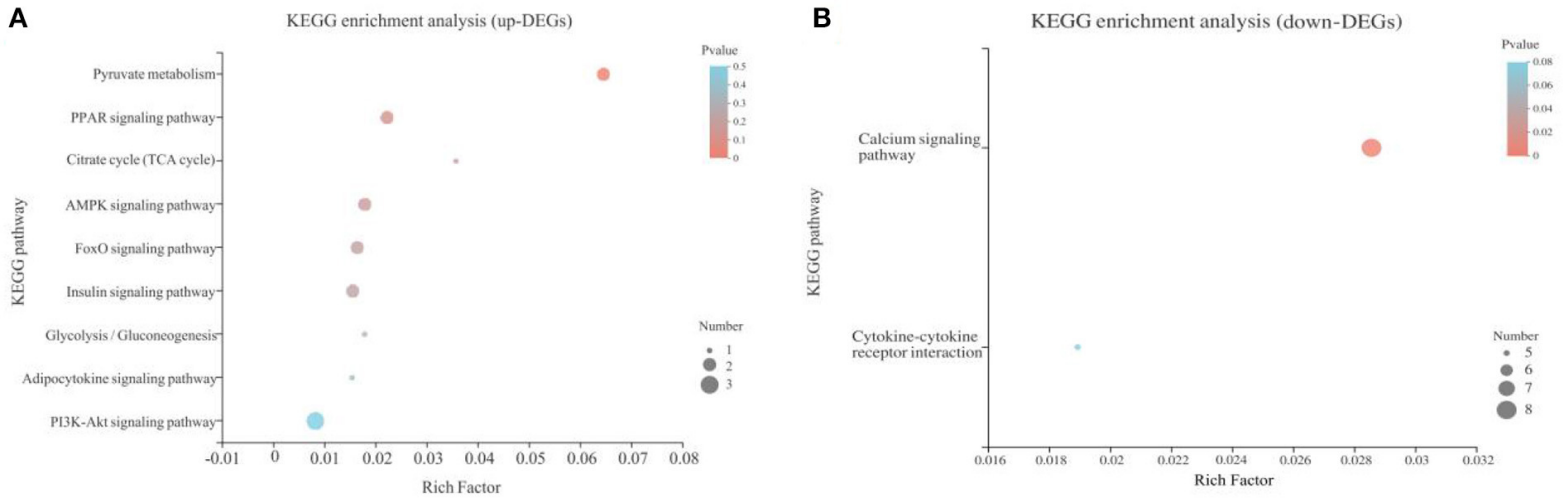
Functional annotation of differentially expressed genes (DEGs) identified in *Bacillus amyloliquefaciens* TL group of broilers in comparison to the control group. **(A)** Kyoto Encyclopedia of Genes and Genomes (KEGG) pathway enrichment analysis of upregulated DEGs. **(B)** KEGG pathway enrichment analysis of downregulated DEGs. The vertical axis represents the name of the pathways, and the horizontal axis represents the Rich factor. The size of the color dot proportionally indicates the number of DEGs enriched in the pathway, while the color dot corresponds to the Q value range.

A total of 358 DEGs (*p* < 0.05) were identified in the liver with 145 upregulated (40.50%) and 213 downregulated (59.50%) in the B.A-TL group (Supplementary Table 8), including four genes (i.e., *AMY2A, SI, PCK1*, and *FASN*) significantly upregulated (*p* < 0.001). The functions of these DEGs were further explored by the KEGG pathway enrichment analyses (Figures 3A,B). The results showed that the “calcium signaling pathway” was the most significantly enriched by the downregulated DEGs between the control and experimental groups, while the upregulated DEGs were significantly enriched in the metabolic pathways of “carbohydrate digestion and absorption,” “starch and sucrose metabolism,” and “PI3K-AKT signaling pathway” (Table 3).

**Table 3:** Kyoto Encyclopedia of Genes and Genomes (KEGG) pathway enrichment analysis of differentially expressed genes (DEGs) identified in the *Bacillus amyloliquefaciens* TL group of broilers.

**DEGs**	***p*** **-value**	**Number of gene**	**KEGG Pathway**
**Up-regulated**		15	
CACNA1D, AMY2A, SI	0.002235	3	Carbohydrate digestion and absorption
LOC101750607, AMY2A, SI	0.002982	3	Starch and sucrose metabolism
PCK1, CDKN1A, HSP90AA1	0.463672	3	PI3K-AKT signaling pathway
PCK1, FASN	0.222832	2	Insulin signaling pathway
PCK1, FASN	0.180216	2	AMPK signaling pathway
FASN	0.117013	1	Fatty acid biosynthesis
PCK1	0.362511	1	Adipocytokine signaling pathway
**Down-regulated**		13	
PLCD4, GRIN2A, DRD5, P2RX7, CAMK1D, PGR2/3, LOC121109428, LOC121112155	0.002702	8	Calcium signaling pathway
CCR8L, TNFSF11, GDF7, GDF9, LOC121112225	0.075272	5	Cytokine-cytokine receptor interaction

### Advanced transcriptome analysis of FASN

**Figure 4:**
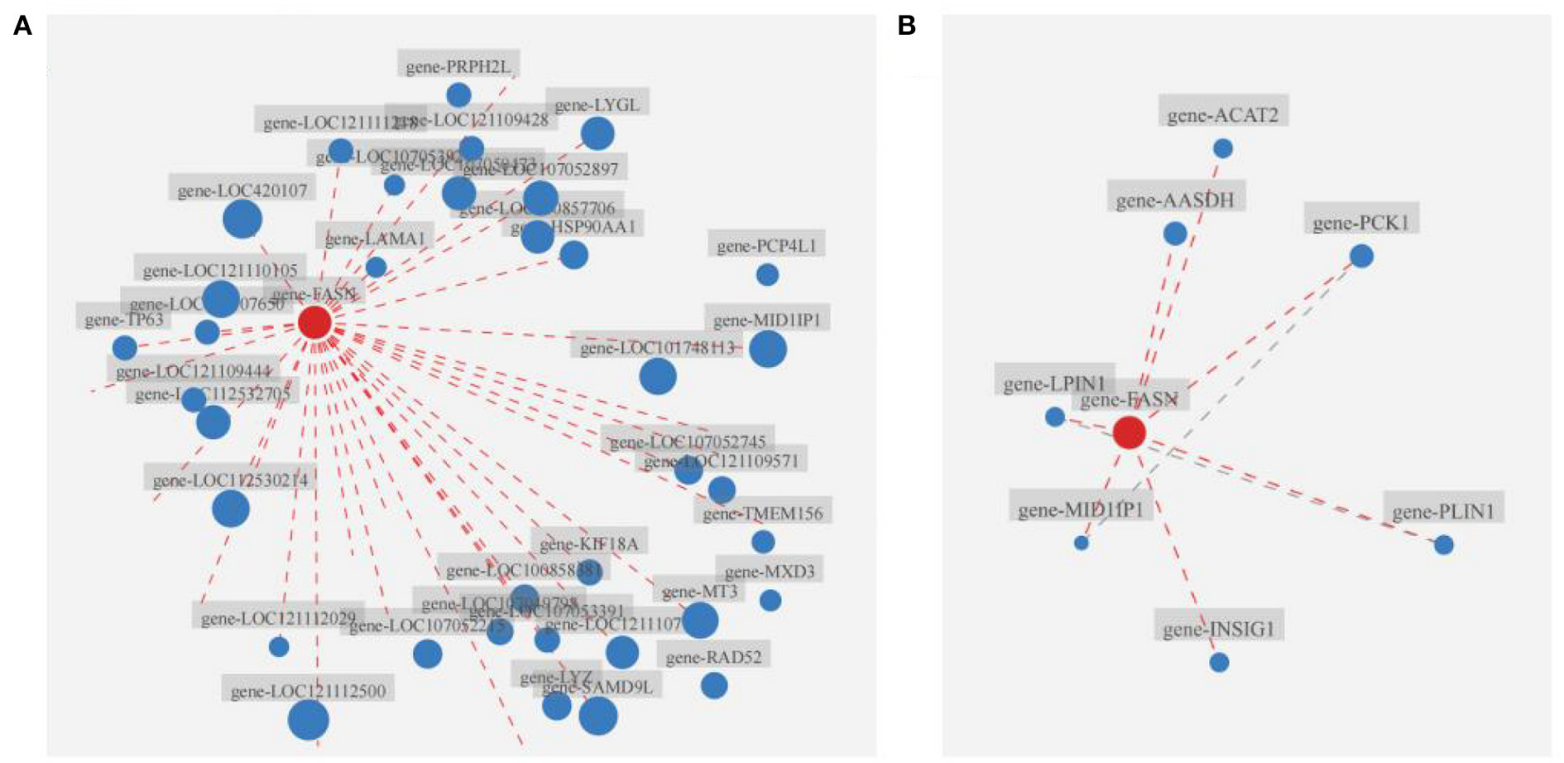
Correlation analysis of *FASN* with other genes and the relationships between FASN and other proteins. **(A)** Correlation analysis of *FASN* with other genes. **(B)** Interaction analysis of FASN with other proteins. Nodes (i.e., red and blue dots) indicate genes/transcripts, and dashed lines indicate the existence of the reciprocal relationship between two genes/transcripts. The size of a node is proportional to its connectivity (i.e., degree), i.e., the more edges connected to the node, the larger degree the node is, indicating the greater importance of the gene/transcript in the network.

Results of the correlation analysis showed that *FASN* and FASN played critical roles in the broiler liver of the B.A-TL group (Figures 4A,B; Supplementary Tables 9A,B), closely correlated with the expression of 36 (*p* < 0.05) out of 358 DEGs and establishing interactions with a total of 7 proteins (*p* < 0.05), respectively. These complex network relationships and the strong connections (i.e., large nodes) of both *FASN* and FASN indicated the significant importance of both *FASN* and FASN in these networks. Therefore, we further investigated the expression changes of both *FASN* and FASN in cellular experiments (below).

### Verification of gene expression patterns based on RNA-Seq by qRT-PCR analysis

To validate the DEGs identified in the liver tissue between the control and the experimental groups based on the RNA-Seq, a total of 12 DEGs (*p* < 0.05; Supplementary Table 3) were randomly selected for qRT-PCR verification. The results of both qRT-PCR and RNA-Seq analyses were consistent, showing similar trends of upregulation and downregulation, thereby validating the RNA-Seq results (Supplementary Figure 5).

### Effects of FST, EPS, and Tyr on the viability and proliferation of LMH cells

**Figure 5:**
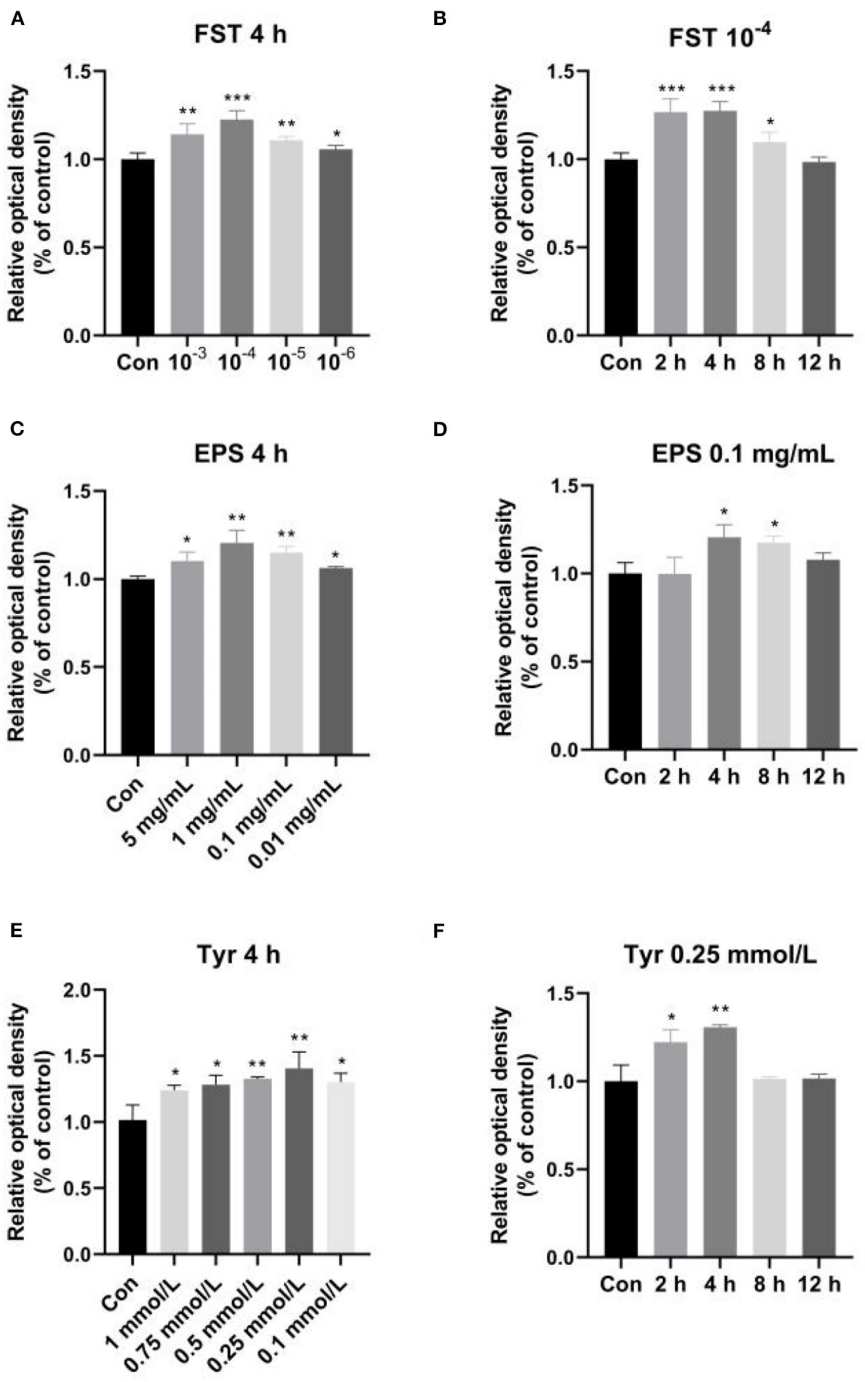
Effects of fermentation supernatant **(A,B)**, exopolysaccharides **(C,D)**, and L-tyrosine **(E,F)** of different concentrations with different lengths of treatment time on LMH cell proliferation. “Con” = control group; “‘FST” = fermentation supernatant group; “EPS” = exopolysaccharides group; “Tyr” = L-tyrosine group. The significance levels for all analyses are set at *p* < 0.05 (*), *p* < 0.01 (**), and *p* < 0.001 (***), respectively.

The effects of FST, EPS, and Tyr on LMH cell proliferation are shown in Figure 5. The results showed that LMH cell proliferation was significantly enhanced by the treatments of FST, EPS, and Tyr with various concentrations and lengths of treatment time. The optimum conditions were observed at 10^−4^ FST (*p* < 0.001), 0.1 mg/mL EPS (*p* < 0.01), and 0.25 mmol/L Tyr (*p* < 0.01) for 4 h, respectively.

### Effects of FST, EPS, and Tyr on the IGF-1 level of LMH cells

**Figure 6:**
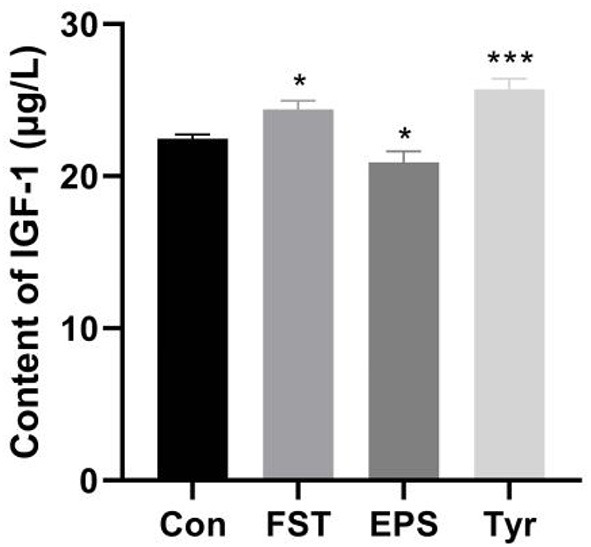
Effects of fermentation supernatant, exopolysaccharide, and L-tyrosine on the IGF-1 level of LMH cells. “Con” = control group; “FST” = fermentation supernatant group; “EPS” = exopolysaccharides group; “Tyr” = L-tyrosine group. The significance levels for all analyses are set at *p* < 0.05 (*), and *p* < 0.001 (***), respectively.

Treatments of LMH cells with 10^−4^ FST (*p* < 0.05) and 0.25 mmol/L Tyr (*p* < 0.001) for 4 h, respectively, significantly increased the IGF-1 secretion in LMH cells, while 0.1 mg/mL EPS (*p* < 0.05) significantly decreased the IGF-1 secretion in LMH cells, suggesting that some metabolites of B.A-TL regulated the IGF-1 secretion in liver cells, ultimately affecting the metabolic activities in the bodies of broilers (Figure 6).

### FST, EPS, and Tyr stimulate lipid metabolism in LMH cells

**Figure 7:**
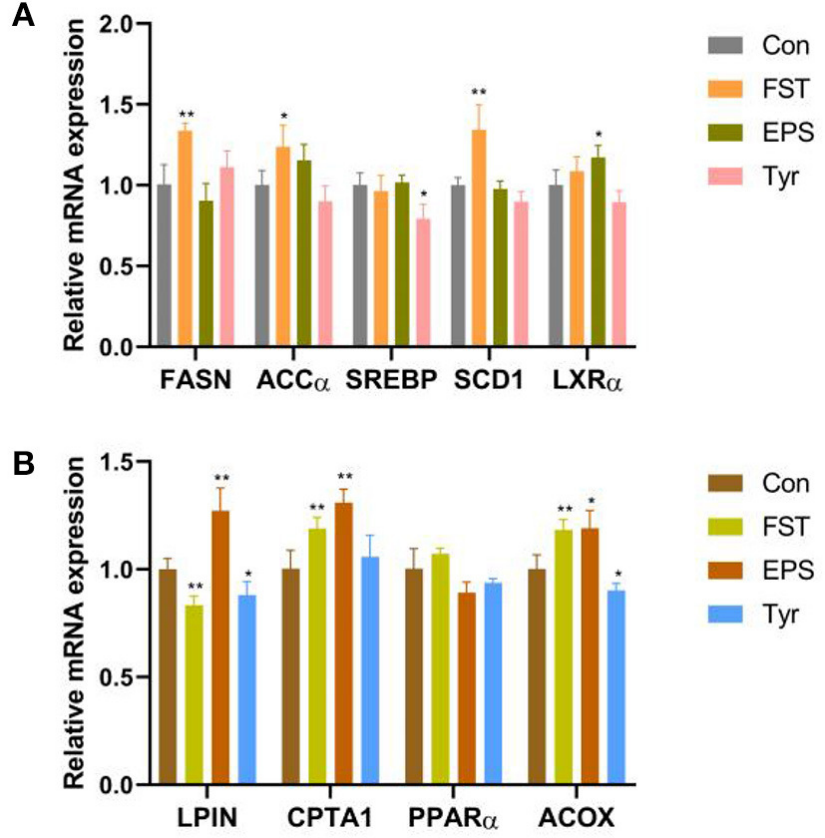
Relative mRNA levels of genes associated with fat synthesis (**A**) and fat oxidation (**B**) in LMH cells treated with fermentation supernatant, exopolysaccharide, and L-tyrosine, respectively. “Con” = control group; “FST” = fermentation supernatant group; “EPS” = exopolysaccharides group; “Tyr” = L-tyrosine group. The significance levels for all analyses are set at *p* < 0.05 (*), and *p* < 0.01 (***), respectively.

**Figure 8:**
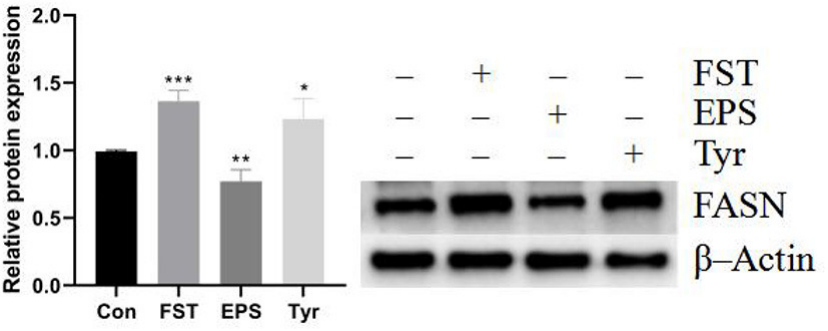
The FASN protein levels of LMH cells treated with fermentation supernatant, exopolysaccharide, and L-tyrosine, respectively. “Con” = control group; “FST” = fermentation supernatant group; “EPS” = exopolysaccharides group; “Tyr” = L-tyrosine group. FASN is used as a target protein; β-Actin is used as a loading standard. The significance levels for all analyses are set at *p* < 0.05 (*), *p* < 0.01 (**), and *p* < 0.001 (***), respectively.

Results of the relative expression levels of genes involved in fat metabolism showed that the B.A-TL metabolites of different components either upregulated or downregulated the genes involved in adipogenesis and fatty acid oxidation, indicating that B.A-TL affected the lipid metabolic activities, i.e., lipid synthesis and decomposition (Figures 7A,B). For example, FST significantly increased the expression of three adipogenesis genes (*p* < 0.05), i.e., *FASN, ACC*α, and *SCD1*, while the expressions of the fatty acid oxidation genes (e.g., *LPIN* and *ACOX*) were either upregulated or downregulated. It was noted that no significant changes were observed in the expression levels of *PPAR*α in both control and experimental groups. Results showed that both FST (*p* < 0.001) and Tyr (*p* < 0.05) of B.A-TL enhanced the expression of FASN in LMH cells, while EPS inhibited the expression of FASN (*p* < 0.01; Figure 8). Therefore, it was speculated that the promotion of fat synthesis by B.A-TL was likely due to the enhanced expression of FASN in hepatocytes stimulated by metabolites of both FST and Tyr.

**Figure 9:**
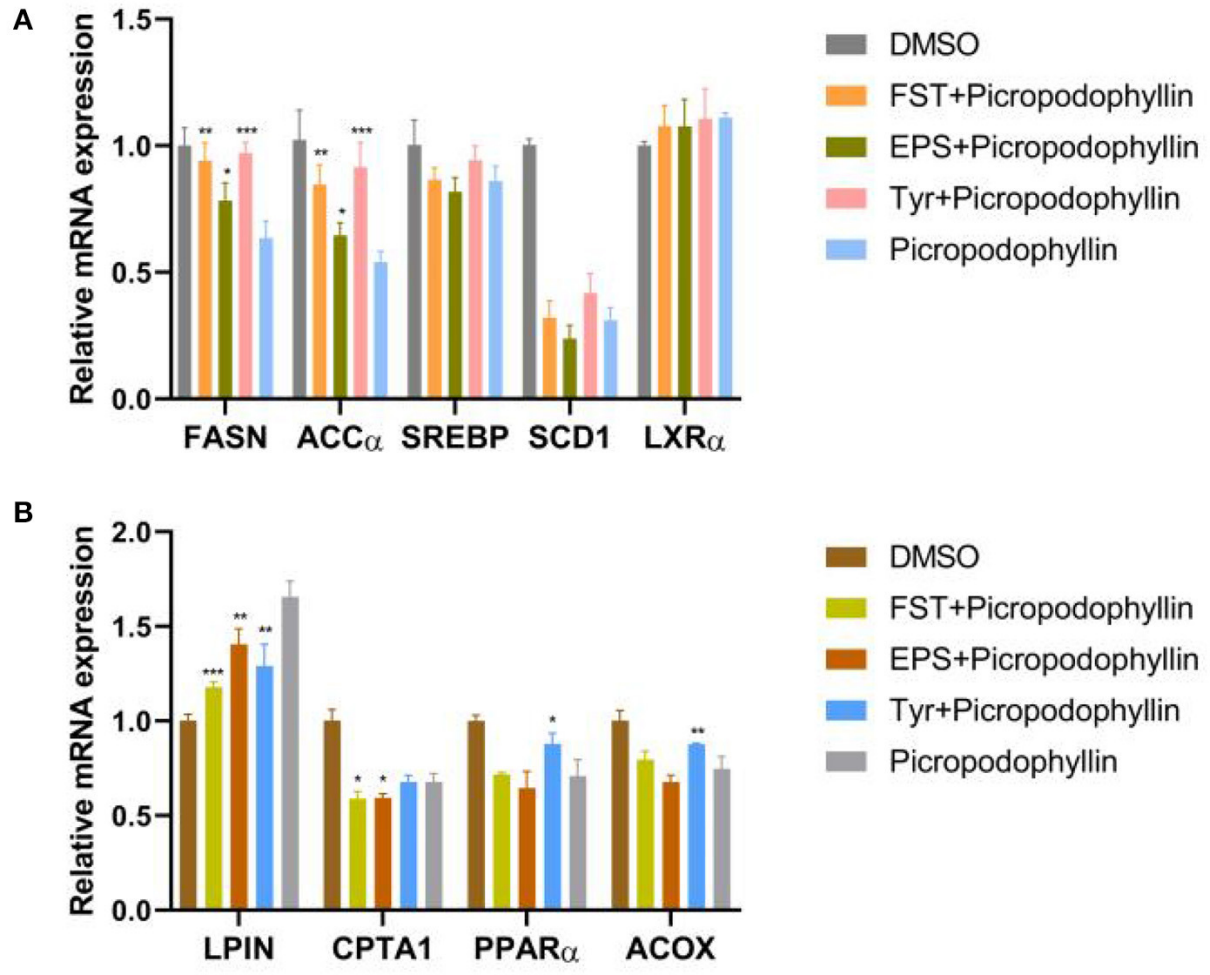
Relative mRNA levels of genes associated with fat synthesis **(A)** and fat oxidation **(B)** in LMH cells treated with fermentation supernatant and IGF-1R signaling inhibitor (i.e., picropodophyllin), exopolysaccharide and picropodophyllin, and L-tyrosine and picropodophyllin, respectively. DMSO is used as a toxicity control. “Con” = control group; “FST” = fermentation supernatant group; “EPS” = exopolysaccharides group; “Tyr” = L-tyrosine group. The significance levels for all analyses are set at *p* < 0.05 (*), *p* < 0.01 (**), and *p* < 0.001 (***), respectively.

To verify the regulation of lipid metabolism by B.A-TL with the involvements of RTKs and their downstream signaling pathways, the lipid metabolism was evaluated in LMH cells treated with the metabolites of B.A-TL and IGF-1R signaling inhibitor, i.e., picropodophyllin (Figure 9). The results showed that compared to the inhibitor group, the inhibitory effect of inhibitors on *FASN* and *ACC*α was alleviated in all three experimental groups (i.e., FST, EPS, and Tyr) with *LPIN* downregulated (*p* < 0.01). *CPTA1* (*p* < 0.05) was downregulated in both FST and EPS groups, while both *PPAR*α (*p* < 0.05) and *ACOX* (*p* < 0.01) were upregulated in the Tyr group. Furthermore, the expressions of *SREBP, SCD1*, and *LXR*α were not significantly altered between the control and experimental groups.

**Figure 10:**
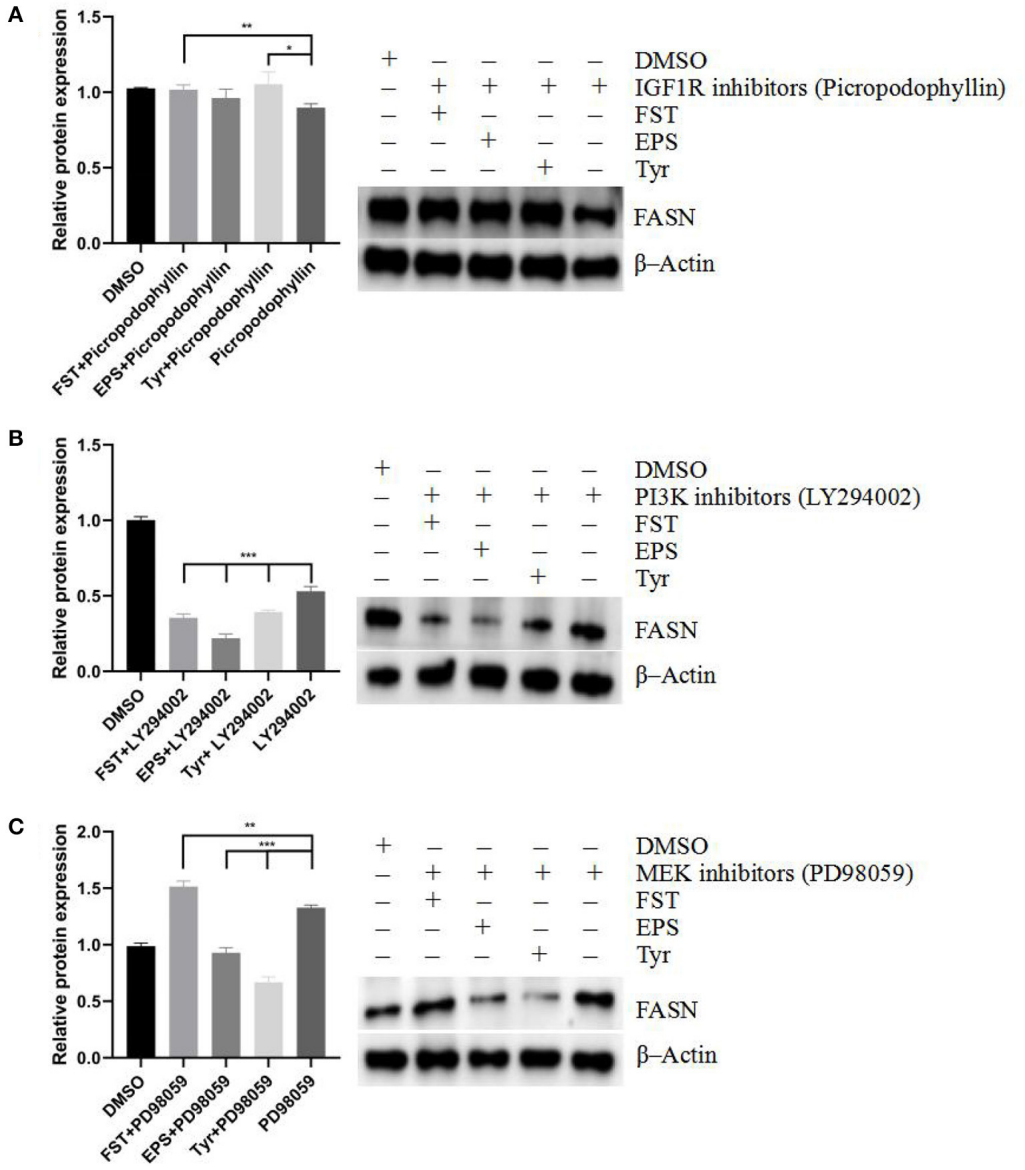
Effects of fermentation supernatant, exopolysaccharide, and L-tyrosine on LMH cells treated with different inhibitors including **(A)** IGF-1R inhibitor (picropodophyllin), **(B)** PI3K inhibitor (LY294002), and **(C)** MEK inhibitor (PD98059), respectively, at the FASN protein level. FASN is used as a target protein; β-Actin is used as a loading standard. “Con” = control group; “FST” = fermentation supernatant group; “EPS” = exopolysaccharides group; “Tyr” = L-tyrosine group. The signi?cance levels for all analyses are set at *p* < 0.05 (*), *p* < 0.01 (**), and *p* < 0.001 (***), respectively.

The effects of different stimulants on the protein expression level of FASN under the treatment of different inhibitors are shown in Figure 10. When the IGF-1R was blocked (Figure 10A), the inhibition of FASN by inhibitors was alleviated in both FST (*p* < 0.01) and Tyr (*p* < 0.05) groups. When the PI3K pathway was blocked (Figure 10B), the expression levels of FASN in all three groups (*p* < 0.001) of FST, EPS, and Tyr were decreased, indicating that the expression of FASN was mainly regulated by the PI3K pathway. Furthermore, when the MAPK pathway was blocked (Figure 10C), the expression of FASN in the FST group was not inhibited (*p* < 0.01) but was decreased in both EPS (*p* < 0.001) and Tyr (*p* < 0.001) groups, suggesting that there were other small molecules in the FST that played an important role in regulating the lipid metabolism. Further studies are needed to verify this speculation. These results suggested that the metabolite FST of B.A-TL mainly affected the PI3K pathway to regulate the expression of FASN, while Tyr played an important role in this regulation as well.

Overall, these results suggested that B.A-TL stimulated both the IGF-1 synthesis and the PI3K signaling pathway (i.e., the downstream of the IGF signaling pathway), promoted the FASN protein expression, and accelerated the lipid metabolism in liver tissue, ultimately promoting the fat deposition in broilers.

## Discussion

### Variations of serum biochemical indices

It is well-known that serum parameters are reliable indicators of health status, reflecting many physiological, nutritional, and pathological changes in the organisms (Koronowicz et al., 2016). In general, the antioxidant enzymes, e.g., CAT, SOD, and T-AOC, are frequently used to assess oxidative damage and to scavenge excessive reactive oxygen species (ROS) from the body (Cecerska-Heryć et al., 2021). Furthermore, fast-growing commercial chickens are susceptible to high environmental temperatures due to their high growth rate (Habibian et al., 2016; Wan et al., 2018), while both oxidative and heat stresses could increase the ROS production and impair muscle quality in broilers (Wan et al., 2018). Numerous studies have shown that probiotics could improve both the antioxidant (Inatomi and Otomaru, 2018; Zhang et al., 2021) and heat stress resistance (Abd El-Hack et al., 2020; Wang et al., 2022) of the body. GLU is the main energy source (Gallenberger et al., 2012), while triglyceride represents another type of energy source (Pillutla et al., 2005). Total cholesterol is a building component of cell membranes (Zhang et al., 2019), while the VLDL is the principal vehicle for the transport of endogenous TG (Mason, 1998). The health status of the liver can be detected from the activity of liver enzymes, i.e., AST and ALT (Mollahosseini et al., 2017). Studies have shown that GH, IGF-1, and INS have significant growth-promoting and anabolic effects (Clayton et al., 2011). In our study, B.A-TL significantly improved the serum physiological indices in broilers, i.e., increasing the contents of CAT, SOD, and T-AOC, while the content of MDA was decreased. Although the ALT content was increased in the serum, no pathological lesions were observed in the livers of broiler chickens. Furthermore, the contents of IGF-1, GLU, and TG in the B.A-TL group were significantly higher than those of the control group, whereas no significant differences were observed in the contents of GH, INS, CHO, and VLDL between the control and the experimental groups. The high GLU content was consistent with the results reported previously, showing that B.A-TL reduced the lipid oxidation and glycolysis activities, suggesting that GLU anabolism (i.e., gluconeogenesis) was active in the B.A-TL group, as reported previously (Hong et al., 2021). The high TG content revealed active lipid metabolism in the B.A-TL group. The results of our study evidently demonstrated that adding B.A-TL into the broiler diet could improve the ABW of the chickens, with the most significant effect observed on day 21, which was consistent with the results reported previously (Hong et al., 2019, 2021). Our results showed that B.A-TL enhanced both the synthesis of TG and the antioxidant and antistress capacities of the body, promoting the growth and improving the muscle quality of broilers.

Liver-derived IGF-1 was revealed with a negative feedback regulatory effect on the secretion of GH (Sjögren et al., 1999), suggesting that liver-derived IGF-1 would inhibit the secretion of GH. Interestingly, it was observed in our study that, without inhibiting the secretion of GH, the B.A-TL promoted the secretion of IGF-1 in the liver of broilers during the brooding period (i.e., younger than 21 days in age). Studies have shown that a large amount of IGF-1 was secreted into the circulation system to promote the rapid development of the muscle, fat, and other tissues, ultimately achieving a significant increase in body weight (Deng et al., 2017), while protein metabolism played a key role in the regulation of the mass of skeletal muscles (Deng et al., 2017; Yoshida and Delafontaine, 2020). Moreover, studies have shown that muscle growth is regulated by IGF-1 and GH *via* the GH/IGF-1 axis (Saleh et al., 2021). Our study showed that the content of IGF-1 in the B.A-TL group was increased by 15.5% compared with that in the control group (*p* < 0.05). These results were consistent with those reported previously, showing that the increased expressions of IGF-1 resulted in the enhanced growth performance of broilers (Deng et al., 2017).

### Functions of probiotic metabolites of B.A-TL

Microbiota-derived metabolites include small molecules, such as amino acids, short-chain fatty acids (FAs) (Dong et al., 2019; Lavelle and Sokol, 2020), and biomolecules, such as peptides and polysaccharides (Kunishima et al., 2019; Hejdysz et al., 2020). It is worth noting that different microorganisms showed varied effects on the body weight, intestinal barrier, and biomolecule metabolism (Wieërs et al., 2020). Therefore, it is necessary to consider the metabolic effects of some bacteria as strain-specific (Wieërs et al., 2020). In our study, we extracted B.A-TL metabolites (i.e., FST) as well as isolated and purified EPS to perform the compositional assays of small molecules in the FST and EPS, and finally selected Tyr and extracellular polysaccharides for subsequent cellular assays. Tyr is a neutral amino acid involved in building essential proteins to provide energy (Dollins et al., 1995) and constitutes the precursor of the catecholamine neurotransmitters, norepinephrine, and dopamine (Lieberman et al., 2005) to effectively respond to acute stress (Lieberman et al., 2005) and enhance cognitive performance (Kühn et al., 2019). Our results showed that Tyr accounted for the highest proportion (29.02%) of the metabolites identified in B.A-TL. Chen et al. (2020) extracted the EPS of *Bacillus amyloliquefaciens* strain AMY-1 and found that the EPS may be involved in the hypoglycemic function of probiotics based on its regulation of blood GLU in mice, while the main components of EPS in strain AMY-1 identified by high-performance liquid chromatography, i.e., mannose, GLU, and galactose, were polymers larger than 1,000 kDa in molecular weight. Our results indicated that EPS of B.A-TL were composed mainly of six sugars, with mannose, GLU, and galactose together accounting for 98.27%, which were consistent with the main components of EPS in strain AMY-1. Furthermore, studies showed that the EPS of strain AMY-1 significantly reduced the blood GLU content of mice (*p* < 0.001) (Chen et al., 2020). Further studies are necessary to verify these explicitly shared regulatory functions of Tyr and EPS in chickens.

### Functional analysis of the liver tissues based on enriched pathways in B.A-TL group

Starch is the most important nutrient in poultry diets (Svihus, 2014; Aderibigbe et al., 2020). On a dry matter basis, the poultry diets may contain up to 50% starch, which is the most important source of energy (Svihus, 2014). The enzymes involved in starch digestion include salivary amylase (AMY1), pancreatic amylase (AMY2), sucrase-isomaltase (SI), and maltase-glucoamylase (MGAM) (Brun et al., 2020; Wolever et al., 2021). Starch must be digested first to initiate a glycemic response, and then GLU is directly absorbed by the organisms. Therefore, differences in starch digestive enzymes may affect the rate and extent of starch digestion and change in the blood glucose levels in body, and consequently, affect the indicators of production performance (Svihus, 2014; Wolever et al., 2021). Studies have shown that the endogenous amylase secretion in broilers is not ideal for the complete digestion of dietary starch (Schramm et al., 2021). However, the prominent digestibility of native starch in chickens compared to other species, such as pigs may be due to the high secretion of amylase induced by the pancreatic juice of chickens (Svihus, 2014), thus increasing the level of amylase to enhance the starch digestion. In our study, it was clearly observed that the serum GLU content of B.A-TL group was significantly increased by 35.92% compared to the control group. Furthermore, the transcriptome results showed that the expressions of genes encoding AMY2A and SI were significantly upregulated, indicating that the increase in serum GLU was associated with the enhanced expression of these two enzymes, while the B.A-TL enhanced the digestion of the body and absorption of starch, ultimately significantly contributing to the fat synthesis in broilers.

Gluconeogenesis is the reverse process of glycolysis (Tang et al., 2018; Jiang et al., 2020), producing GLU from non-carbohydrates, such as pyruvate, lactic acid, and glycerol (Bhalla et al., 2014). Phosphoenolpyruvate carboxykinase 1 (PCK1) is one of the key enzymes of gluconeogenesis (Jiang et al., 2020). The overexpression of *PCK1* gene activated gluconeogenesis but inhibited the glycolysis pathway (Chong et al., 1994), revealing the key role of PCK1 protein kinase activity in SREBP-dependent lipid synthesis (Tang et al., 2018; Xu et al., 2020). As a complex, active, and dynamically changing multifunctional organelle, the lipid droplet is mainly composed of TG and sterol esters and is involved in lipid metabolism and storage as well as protein storage and degradation (Ploegh, 2007), membrane transport (Bartz et al., 2007), signal transduction, and other metabolic processes (Cohen et al., 2011). Generally, adipocytes grow and accumulate lipids with limits in order to avoid tissue damage lesions (Hirsch and Batchelor, 1976). Our results showed that serum IGF-1 content was increased and the expression of *PCK1* gene was upregulated in BA-TL group, and the results of liver Oil Red O staining showed that the B.A-TL promoted lipid droplet formation, indicating that B.A-TL enhanced gluconeogenesis to produce TG *via* IGF-1-regulated GLU internalization process.

Studies have shown that calcium regulates adipocyte metabolism by affecting intracellular calcium levels and fatty acid absorption in the gastrointestinal tract (Fleischman et al., 1966; Shi et al., 2001). He et al. (2012) found that an early low-calcium diet caused an increased fat mass and body weight, and ultimately became significant when rats were exposed to a late high-fat diet. Furthermore, Ca^2+^ in hepatocytes is an important regulator of GLU and lipid metabolism, bile secretion, mitochondrial activity, cell growth, and apoptosis (Berridge et al., 2000; Barritt et al., 2008; Amaya and Nathanson, 2013; Longo et al., 2019). The activity of hepatocytes is regulated by hormones and growth factors that utilize changes in Ca^2+^ concentration as intracellular signals (Amaya and Nathanson, 2013), while many of the cellular effects of Ca^2+^ are mediated by the Ca^2+^ binding protein calmodulin (CaM) (Zayzafoon, 2006). Although lipid metabolism in the liver is normally balanced without excessive lipid accumulation, when this homeostasis is disrupted, the lipid droplets are accumulated in hepatocytes, leading to cytotoxicity (Ali and Petrovsky, 2019). Excess lipid droplets may also impair the effects of IGF-1 (Petersen and Shulman, 2018), while the hepatic lipid accumulation may reflect increased hepatic lipid influx and hepatic lipogenesis (Maus et al., 2017). Recently, impaired calcium signaling was identified as a cause of increased endoplasmic reticulum (ER) stress, leading to hepatic lipid accumulation (Maus et al., 2017; Ali and Petrovsky, 2019). Studies have shown that the inhibition of store-operated calcium entry (SOCE) exacerbates the lipid accumulation, while the reduction of intracellular Ca^2+^ may cause lipid accumulation in fatty liver cells (Wilson et al., 2015) and fat storage cells (Baumbach et al., 2014). In turn, excessive lipid accumulation in hepatocytes also inhibits the SOCE (Wilson et al., 2015) and reduces the Ca^2+^ content in ER (Park et al., 2010; Baumbach et al., 2014; Wilson et al., 2015). The ER stress response initiated by low ER-Ca^2+^ stimulates the synthesis of both diacylglycerol and TG (Park et al., 2010; Arruda and Hotamisligil, 2015) utilizing these excess FAs, ultimately leading to the deposition of lipids in the cytoplasm (Ali and Petrovsky, 2019). Our study showed that the hepatic calcium pathway was downregulated in the B.A-TL group, which was consistent with the fact that low calcium caused hepatic lipid accumulation. However, our results of liver HE staining and serum biochemical indices showed that this downregulation of the hepatic calcium pathway was a reflection of the physiological regulation without causing liver lesions or affecting the healthy growth of broilers, strongly indicating that B.A-TL could indeed promote liver lipid metabolism in broilers.

### Regulations of IGF/PI3K/FASN pathway

In birds, lipogenesis occurs mainly in the liver, while the adipocytes function as a storage site for TG (Claire D'Andree et al., 2013). It is estimated that 80–85% of the FAs presented in TG stores of broiler adipose tissue are derived from hepatic lipogenesis or absorbed from the diet *via* the intestine (Richards et al., 2003), while the lipogenic activity in the liver is much greater than that in adipose tissue in chickens (Claire D'Andree et al., 2013). Studies showed that when the levels of saccharides exceeded the immediate energy requirements, GLU was converted into storage lipids (*via* lipogenesis) after the saturation of tissue glycogen deposition (Massari et al., 2016). Furthermore, fat is one of the factors affecting meat flavor, while the intermuscular fat affects the color, taste, and savory of meat (Claire D'Andree et al., 2013; Peña-Saldarriaga et al., 2020). Therefore, it is practically important to regulate the fat deposition in broiler production (Claire D'Andree et al., 2013). Hong et al. (2019) found an increase in the relative abundance of *Firmicutes* in the cecum microbiota (*p* < 0.01). It has been pointed out that a higher relative abundance of *Firmicutes* triggers energy storage, resulting in higher fat deposition (Wang et al., 2021), while fat deposition increases faster and earlier in fast-growing chickens compared to slow-growing chickens (Lilja, 1983; Carlborg et al., 2003). The liver is the major site for fatty acid biosynthesis regulated by a complex molecular network that controls the hepatic lipid composition and regulates systemic lipid metabolism (Liao et al., 2018). This molecular network contains various transcription factors, such as SREBP-1c, LXR, and PPAR, and pathways, such as PI3K/AKT (Wang et al., 2015; Titchenell et al., 2016). The positive effect of the PI3K/AKT signaling axis on lipid metabolism has been illustrated in hepatocytes and adipocytes, upregulating the key lipogenic enzymes, including FASN, SCD1, and ACCα (Wan et al., 2011; Liao et al., 2018). These results were consistent with those reported previously, showing that the interactions between the nutrients and the synthesis and activity of lipogenic enzymes were the possible causes of lipid deposition in adipose tissue (Peña-Saldarriaga et al., 2020).

FASN is a large multi-enzyme complex with a single protein of ~270 kDa (Maier et al., 2006). FASN catalyzes the synthesis of palmitic acid based on both acetyl-CoA and malonyl-CoA and plays a role as a central regulator of lipid metabolism. It is considered to be an important protein in the liver under normal physiological conditions, controlling the mechanism of liver TG synthesis. As the carbohydrates become abundant, GLU is converted to FAs catalyzed by FASN, and the excess FAs are then assembled into TG and stored as fat droplets or secreted as VLDL (Ventura et al., 2015; Fhu and Ali, 2020). Studies showed that the G4928024A of the *FASN* gene is significantly correlated with fat bandwidth and abdominal fat percentage (Claire D'Andree et al., 2013). Furthermore, studies have shown that FASN expression is regulated by SREBP-1c, NF-Y, AZGP1, NAC1, and PKD1 (Chang et al., 2019; Li et al., 2021), which are in turn modulated by PI3K/AKT/mTOR (Li et al., 2012; Chang et al., 2019; Zhu et al., 2019), ERK/MAPK (Chang et al., 2019), and Wnt/β-catenin (Zhang et al., 2020) pathways. Moreover, the expression of FASN is downregulated after the PI3K/AKT/mTOR pathway is inhibited (Li et al., 2012; Han et al., 2015). Han et al. (2015) showed that INS mediated the lipid deposition in goose hepatocytes *via* the PI3K/AKT/mTOR signaling pathway. The results of Oil Red O staining reported in the previous studies revealed that lipid accumulation occurred in a dose-dependent manner following the addition of INS, and that 50, 100, or 150 nmol/L INS dose-dependently increased mRNA expression levels of lipogenesis-related genes (e.g., *SREBP-1, FASN*, and *ACC*α) and increased both FASN and ACCα protein levels (Han et al., 2015). Furthermore, the PI3K inhibitor treatment reduced insulin-induced increases in TG content, lipid content, mRNA levels of genes involved in adipogenesis (e.g., SREBP-1, FASN, and ACCα) and protein levels of FASN and ACCα (Han et al., 2015). Our study showed that both FST and Tyr in B.A-TL stimulated the expression of FASN in LMH cells, suggesting that B.A-TL promoted the synthesis of body fat which could be induced by the metabolic product stimulation of FASN protein expression, probably with both FST and Tyr involved and playing an important role. When IGF-1R was blocked, the treatments of FST, EPS, and Tyr on the LMH cells alleviated the inhibition of FASN by inhibitors. When the PI3K pathway was blocked, the expression levels of FASN in LMH cells treated with FST, EPS, and Tyr were significantly decreased, indicating that the expression of FASN was mainly regulated by the PI3K pathway. Furthermore, when the MAPK pathway was blocked, the expression of FASN in the FST group was not inhibited, suggesting that besides Tyr, other substances in the supernatant could also regulate FASN expression. Future studies are necessary to verify the existence of these substances and their functions in the expression of FASN.

In summary, these results showed that the FASN protein expression in the experimental groups was significantly downregulated with the treatment of the PI3K/AKT pathway inhibitor LY294002, suggesting that B.A-TL metabolites (Tyr and other components in the supernatant) stimulated the IGF-1 synthesis, activated the IGF/PI3K signaling pathway, promoted FASN protein expression, accelerated lipid metabolism in the liver tissue, and ultimately promoted fat deposition in broilers.

## Conclusion

The results of our *in vivo* experiments indicated that B.A-TL changed the expression of relevant genes to stimulate the secretion of IGF-1 in the liver tissue of broilers without affecting the secretion of GH and to enhance the antioxidant and antistress capacities of the organism. With the treatment of B.A-TL, a large amount of IGF-1 was secreted into the circulatory system to promote the rapid growth of muscle tissue, thus promoting the rapid weight gain of broilers during the brooding period (i.e., younger than 21 days in age). Our *in vitro* experiments showed that B.A-TL metabolites (i.e., FST and Tyr) stimulated the secretion of IGF-1 in LMH cells, altered the expression of fat metabolism genes, improved the expression of FASN protein, and promoted the synthesis of lipid substances in the liver. Our study is the first report demonstrating that probiotic B.A-TL promotes IGF-1 secretion by the liver of birds and its role in lipogenesis *via* IGF/PI3K/FASN pathway. These novel findings provide strong experimental evidence to support the application of B.A-TL in the regulatory studies of hepatic lipid metabolism in birds (i.e., fast-growing broilers) and to promote the commercial fatty liver production of poultry, such as Landes geese.

## Data availability statement

The data presented in the study are deposited in the NCBI/Bioproject repository, accession number PRJNA835984 (https://www.ncbi.nlm.nih.gov/bioproject?term=PRJNA835984&cmd=DetailsSearch).

## Ethics statement

The animal study was reviewed and approved by Animal Experiments at the College of Veterinary Medicine, Huazhong Agricultural University.

## Author contributions

Conceptualization: PC, ZZ, DS, ZL, and YX. Methodology: PC, XW, XL, and YX. Software, formal analysis, data curation, and writing—original draft preparation: PC. Validation: PC and SL. Writing—review and editing: YX. All authors have read and agreed to the published version of the manuscript.

## Funding

This research was funded by the National Key Research and Development Program of China (Grant number 2017YFD05010001) and the Innovative Job Funds of Agricultural Science and Technology of Hubei Province (Grant number 2019-620-000-001-30).

## Conflicts of interest

The authors declare that the research was conducted in the absence of any commercial or financial relationships that could be construed as a potential conflict of interest.

## Publisher's note

All claims expressed in this article are solely those of the authors and do not necessarily represent those of their affiliated organizations, or those of the publisher, the editors and the reviewers. Any product that may be evaluated in this article, or claim that may be made by its manufacturer, is not guaranteed or endorsed by the publisher.

## Abbreviations

B.A-TL: *Bacillus amyloliquefaciens* TL
IGF-1: insulin-like factor 1
FST: fermentation supernatant
EPS: exopolysaccharides
L-Tyr: L-tyrosine
LMH: chicken hepatocellular carcinoma cell line
IGF-1R: insulin-like growth factor 1 receptor
PI3Kα: phosphoinositide 3-kinase α
MEK: MAP kinase
GLU: glucose
TG: triglycerides
PCK1: phosphoenolpyruvate carboxykinase
FASN: fatty acid synthase
ACCα: acetyl CoA carboxylase α
GH: growth hormone
INS: insulin
CHO: cholesterol
VLDL: very low-density lipoprotein
ALT: alanine aminotransferase
AST: aspartate aminotransferase
MDA: malondialdehyde
CAT: catalase
SOD: superoxide dismutase
T-AOC: total antioxidant capacity
GSH-Px: glutathione peroxidase
IR: insulin receptor
RTK: receptor tyrosine kinases
FA: fatty acid
SREBP1: sterol regulatory element binding protein 1
SCD: stearoyl-CoA desaturase
ABW: average body weight
ADG: average daily gain
FCR: feed conversion ratio
LXRα: liver X receptor α
LPIN1: lipid phosphate phosphohydrolase 1
CPT1A: carnitine palmitoyltransferase 1A
PPARα: peroxisome proliferators-activated receptors α
ACOX1: acyl-Coenzyme A oxidase 1
SDS-PAGE: sodium dodecyl sulfate-polyacrylamide gel electrophoresis
LC-MS: liquid chromatography and mass spectrometry
TPM: transcripts per million
DEGs: differentially expressed genes
GO: Gene Ontology
KEGG: Kyoto Encyclopedia of Genes and Genomes
AMY1: salivary amylase
AMY2: pancreatic amylase
SI: sucrase-isomaltase
MGAM: maltase-glucoamylase
LDs: lipid droplets
SOCE: store-operated calcium entry
DG: diacylglycerol.
