# Supplementary material for: Liver fat metabolism of broilers regulated by Bacillus amyloliquefaciens TL via stimulating IGF-1 secretion and regulating the IGF signaling pathway
Source: Front Microbiol. 2022 Jul 27;13:958112. doi: 10.3389/fmicb.2022.958112 (PMC9363834; doi:10.3389/fmicb.2022.958112)
Supplement: Supplementary file 1 [file Data_Sheet_1.docx]

Supplementary Material

1. **Supplementary Data**

**
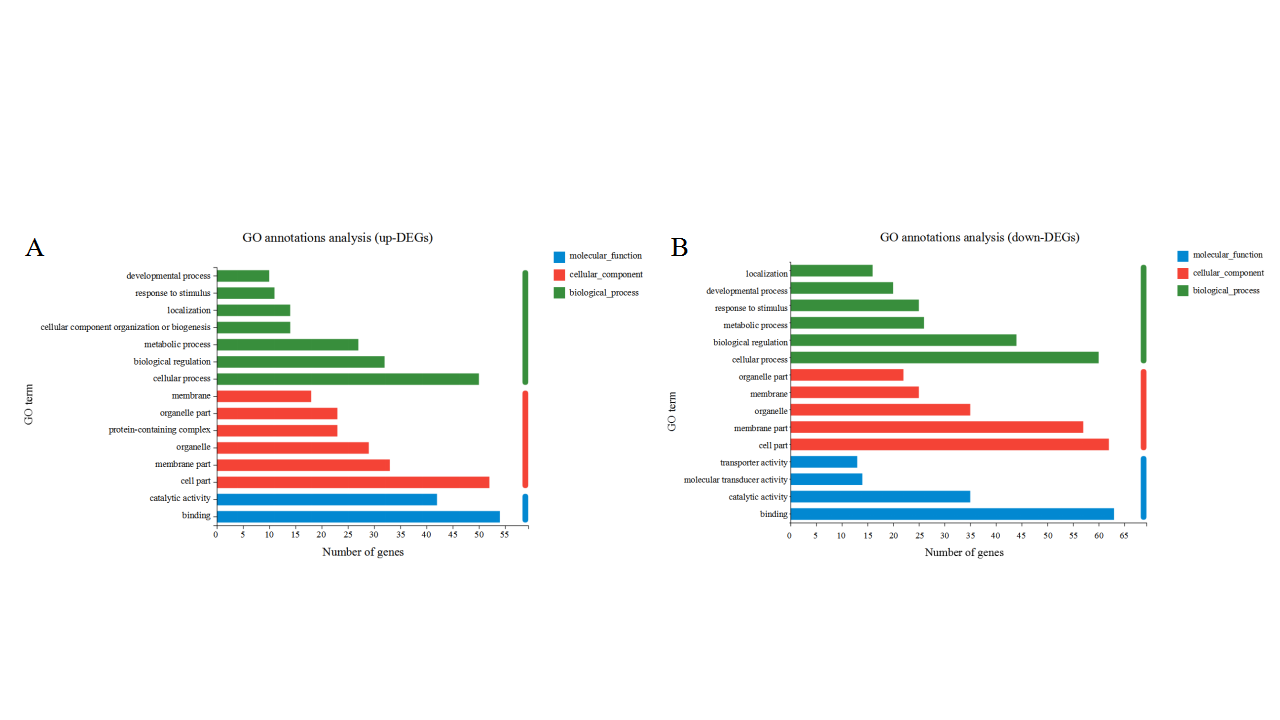
**

**Supplementary Data 1.** Functional annotation of differentially expressed genes (DEGs) identified in *Bacillus amyloliquefaciens* TL group of broilers in comparison to the control group. (**A**) Gene ontology (GO) enrichment analysis of the up-regulated DEGs between control and experimental groups. (**B**) GO enrichment analysis of the down-regulated DEGs between control and experimental groups. “BP” = biological process, “CC” = cellular component, and “MF” = molecular function.

The results of GO annotation analysis of these DEGs showed that the functions of DEGs were classiﬁed into three categories, including biological process (BP), cellular component (CC), and molecular function (MF). The enriched GO terms of the DEGs were shown in Data 1A, showing that a high proportion of the DEGs up-regulated in the B.A-TL group were involved in MFs and BPs, including “catalytic activity,” “protein binding,” “biosynthetic process,” and “organic substance biosynthetic process” categories (Supplementary Table S10A). The enriched GO terms of the down-regulated DEGs in the B.A-TL group were revealed in the CCs and BPs, i.e., “intrinsic/integral component of membrane” and “response to stimulus/stress” categories (Data 1B; Supplementary Table S10B).

1. **Supplementary Figures and Tables**

## Supplementary Figures


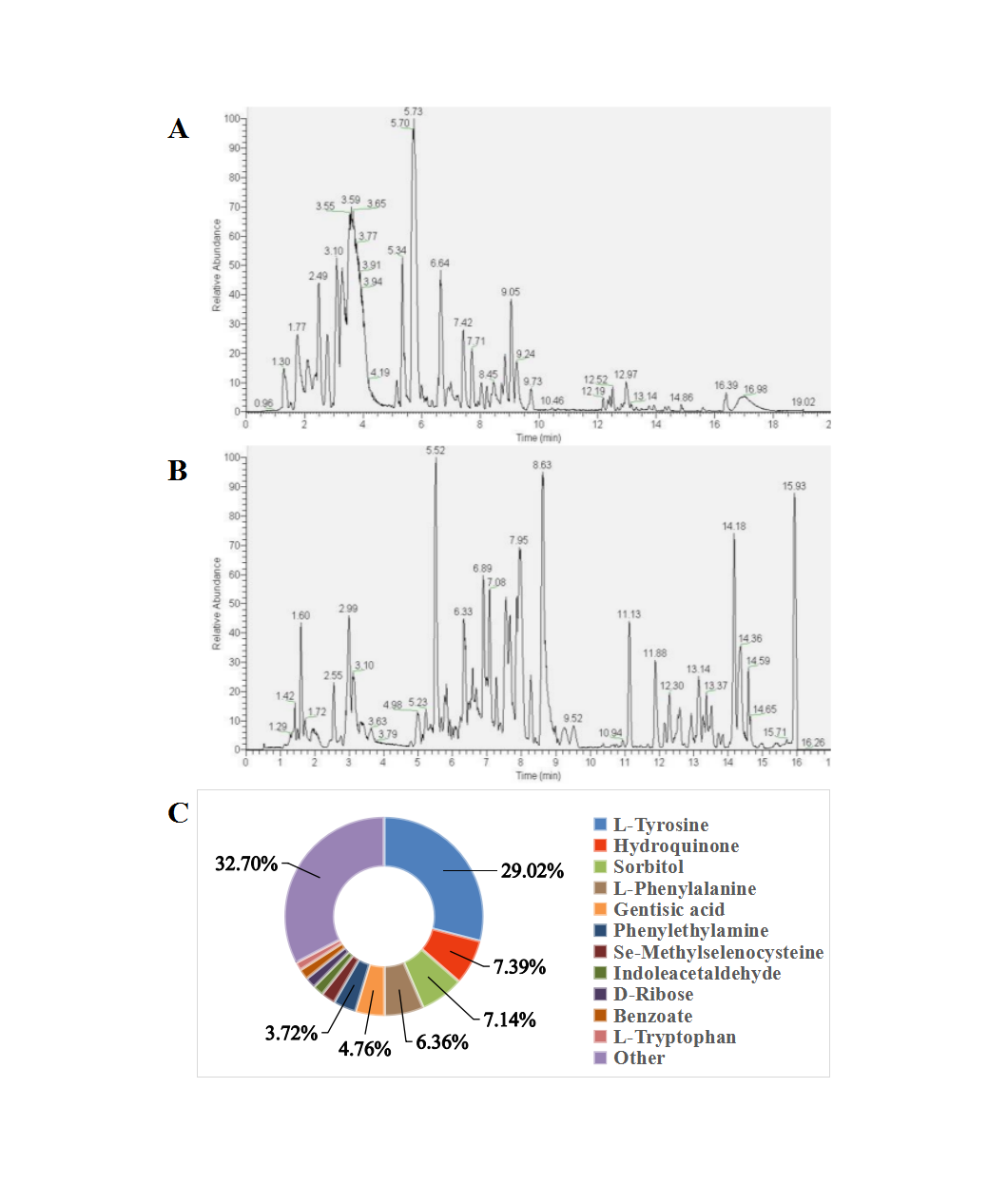


**Supplementary Figure 1.** Metabolic groups of fermentation supernatant (FST) of *Bacillus amyloliquefaciens* TL. (**A**) Sample positive ion spectrogram. (**B**) Sample negative ion spectrogram. (**C**) Components of FST in *B. amyloliquefaciens* TL.

**
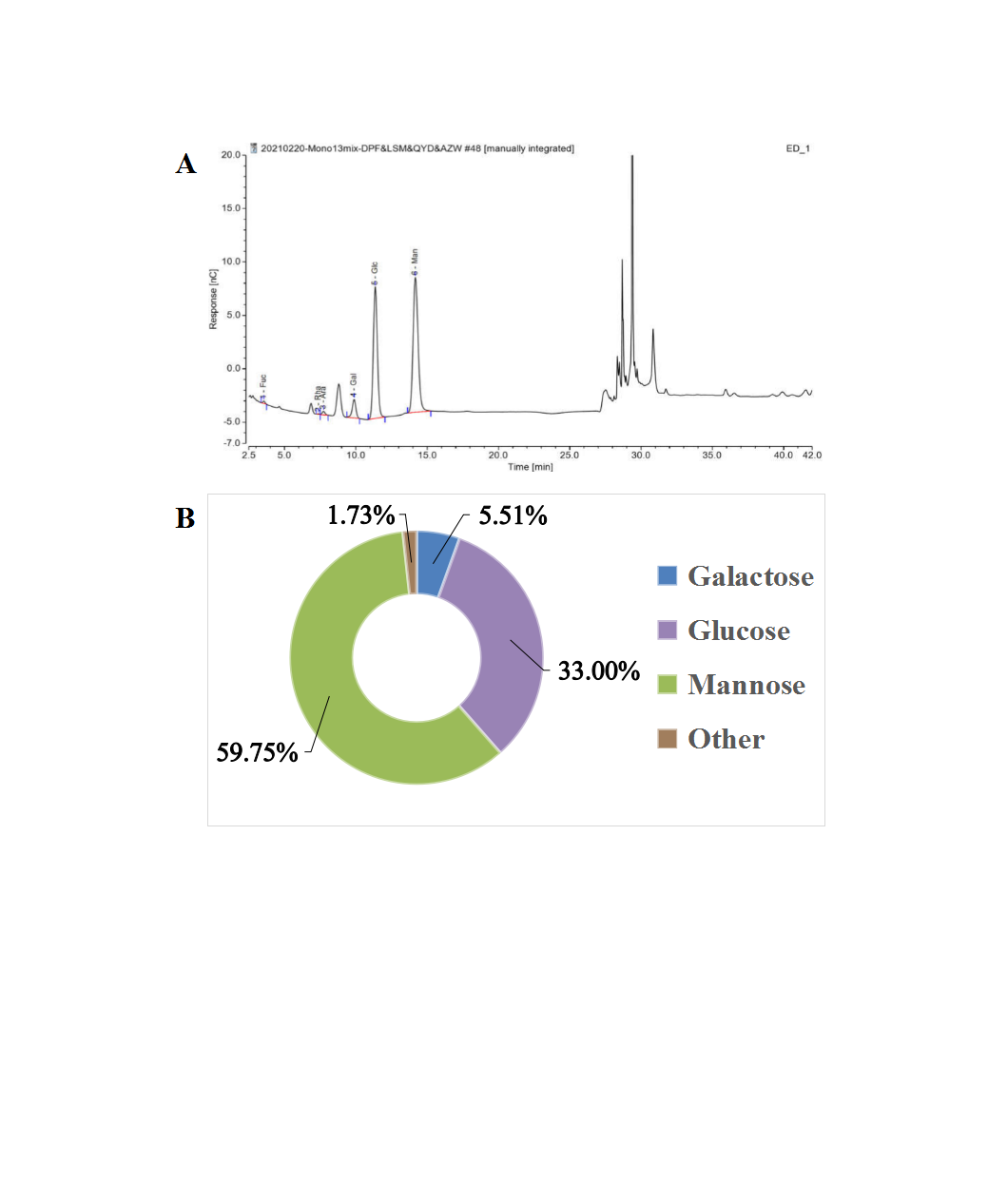
**

**Supplementary Figure 2.** Metabolism of exopolysaccharides (EPS) of *Bacillus amyloliquefaciens* TL. (**A**) Sample ion chromatography. (**B**) Components of EPS in *B. amyloliquefaciens* TL.

**
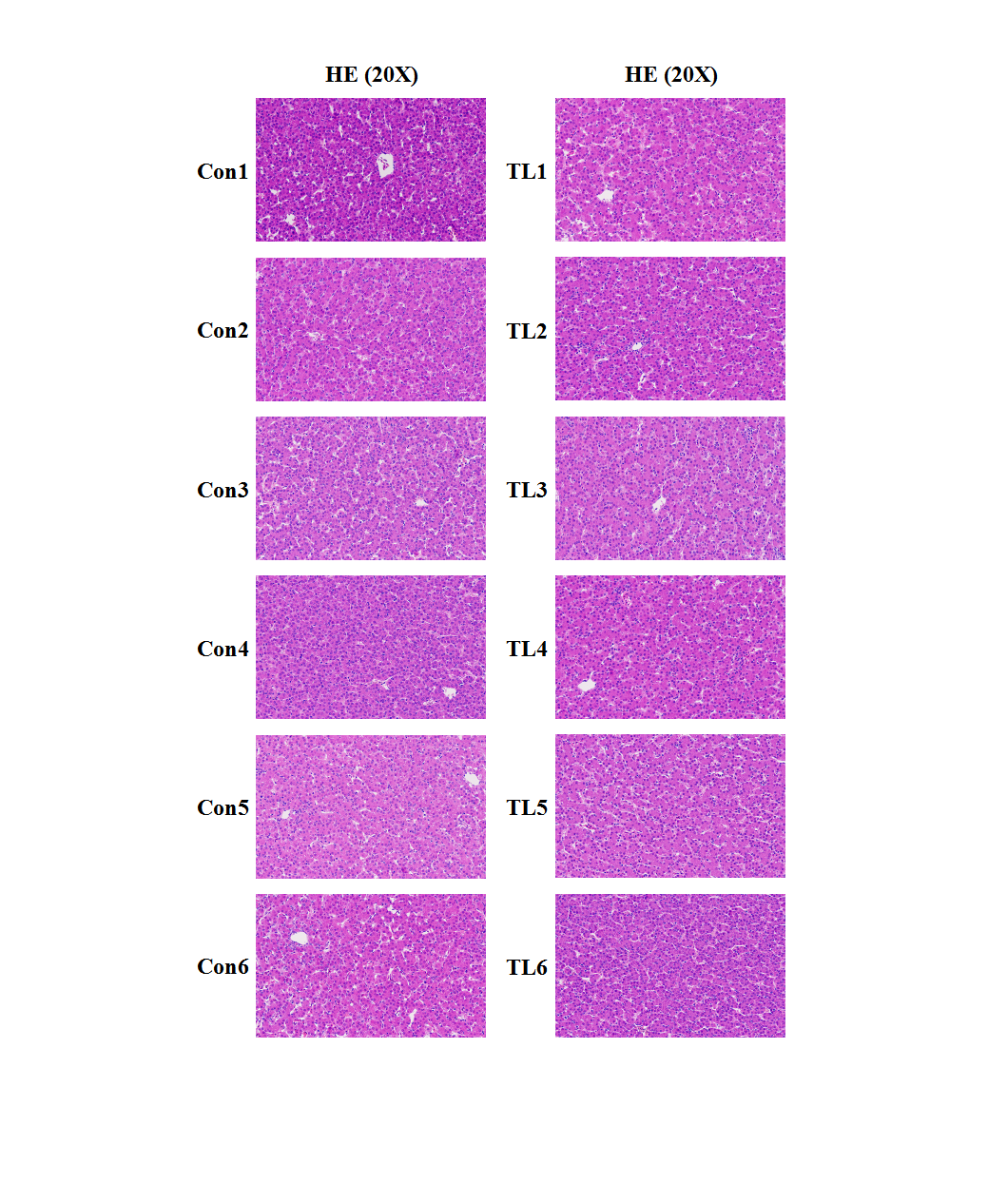
**

**Supplementary Figure 3.** The liver tissues stained with hematoxylin and eosin (HE) of broilers in the control and experimental groups at 21 days of age. “Con” = control group; “TL” = *Bacillus amyloliquefaciens* TL experimental group.

**
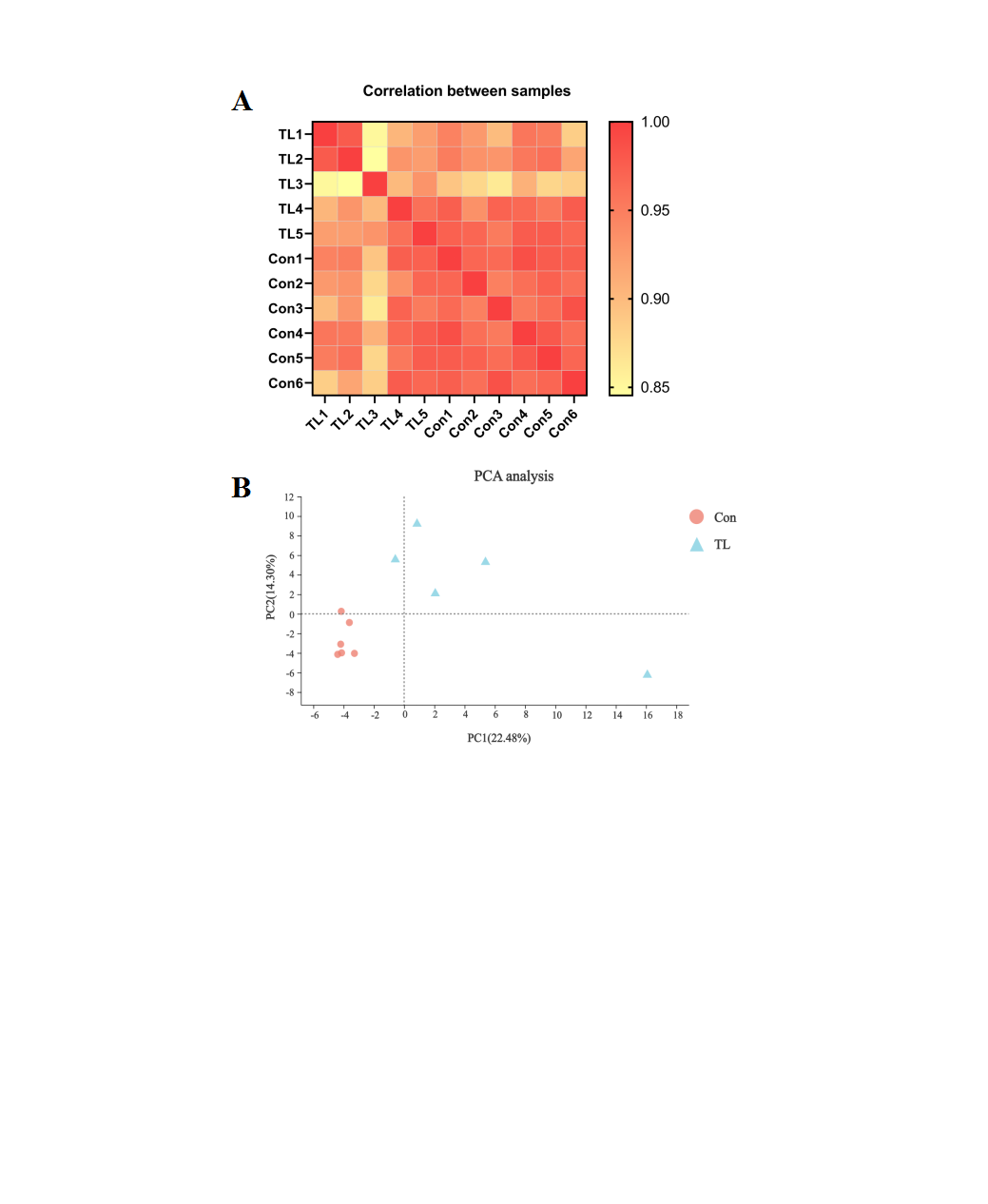
**

**Supplementary Figure 4.** Transcriptome proﬁles of liver tissues from 21-day-old broilers in both the control and experimental groups. (**A**) Correlation coefﬁcient heat map between individual samples. The value is presented as the square of the Pearson correlation coefﬁcient (R^2^). (**B**) Principal component analysis (PCA) of transcriptomes of the control and probiotics groups. “TL” = *Bacillus amyloliquefaciens* TL experimental group; “Con” = control group.

**
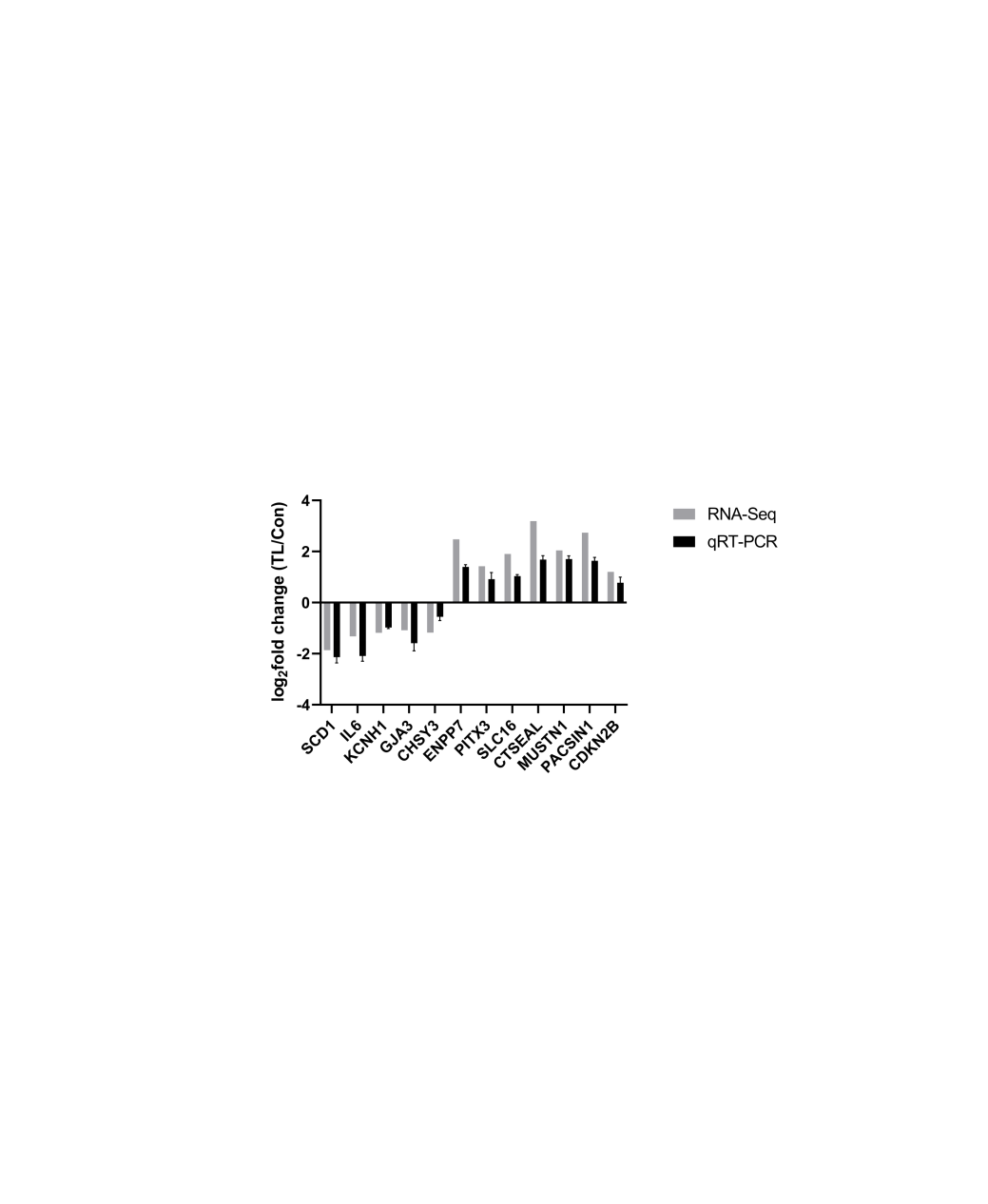
**

**Supplementary Figure 5.** Validation by quantitative real-time PCR (qRT-PCR) analysis of the differentially expressed genes (DEGs) identified in broilers treated with *Bacillus amyloliquefaciens* TL based on RNA-Seq analysis.

## Supplementary Tables

**Supplementary Table 1.** Composition of experimental diets used in this study. The components of diet include zinc 80 mg, iron 80 mg, copper 8 mg, iodine 0.45 mg, selenium 0.20 mg, manganese 100 mg, vitamin A 14,000 IU, vitamin D3 2,800 IU, vitamin E 23.80 IU, vitamin K3 1.96 mg, vitamin B12 0.025 mg, vitamin B2 8.4 mg, pantothenic acid 15 mg, niacin 1.12 mg, vitamin B6 4.75 mg, biotin 0.10 mg, vitamin B1 2.50 mg, choline 1100 mg, and folic acid 50 mg.

| **Diet composition** | **Content (%)** |
| --- | --- |
| **Ingredient** |  |
| Corn, yellow | 59.00 |
| Soybean meal, dehulled (CP, 48%) | 31.00 |
| Fish meal | 2.50 |
| Soybean oil | 2.50 |
| wheat bran | 1.45 |
| Limestone | 1.10 |
| Salt | 0.30 |
| Calcium hydrogen phosphate | 1.40 |
| DL-methionine | 0.15 |
| Mineral and vitamin premix (supplied per kilogram) | 0.60 |
| **Analyzed nutrient** |  |
| Crude protein | 19.80 |
| Calcium | 0.896 |
| Phosphorus | 0.60 |
| Lysine | 1.16 |
| Methionine | 0.36 |
| Met + Cys | 0.63 |
| ME (MJ/kg) | 12.40 |

**Supplementary Table 2.** Information of sampled individuals.

| **Group** | **Serial number** | **Number** | **Pen** | **Body weight (21 day) (g)** |
| --- | --- | --- | --- | --- |
| B.A-TL | TL1 | 5 | 1 | 813 |
|  | TL2 | 8 | 1 | 711 |
|  | TL3 | 1 | 3 | 797 |
|  | TL4 | 2 | 5 | 800 |
|  | TL5 | 5 | 6 | 770 |
|  | TL6 | 9 | 2 | 713 |
| Con | Con1 | 8 | 7 | 727 |
|  | Con2 | 7 | 7 | 714 |
|  | Con3 | 5 | 8 | 717 |
|  | Con4 | 5 | 9 | 700 |
|  | Con5 | 3 | 11 | 678 |
|  | Con6 | 2 | 12 | 702 |

**Supplementary Table 3.** Primers and their sequences used in the qRT-PCR validation experiments of the RNA-Seq data in this study. “F” and “R” in the names of the primers indicate “forward” and “reverse” primers, respectively.

| **Primer** | **Sequence (5’–3’)** |
| --- | --- |
| β-Actin-qF | GGGGAAAGTCATCCCTGAGC |
| β-Actin-qR | TTGGCTGGTTTCTCCAGACG |
| SCD1-qF | TGCTGAACATCTGAGGGTGC |
| SCD1-qR | CATTTCCTTGTTGCTGGGGC |
| IL6-qF | CGCCTTTCAGACCTACCTGG |
| IL6-qR | CTTCAGATTGGCGAGGAGGG |
| KCNH1-qF | CACCGAGGTCAAGCATTCCT |
| KCNH1-qR | TGGAGACTGGGGCCTTGATA |
| ENPP7-qF | GGGCGAGATGATGTTCCCTT |
| ENPP7-qR | ACATCCTGGTCGTAGTCCCA |
| PITX3-qF | AGAACCAGACAGACGACTCCA |
| PITX3-qR | GTTTTTGAACCAGACCCGCACTC |
| SLC16-qF | CTTGCATCGGTGTTTCCCC |
| SLC16-qR | GTGACCAGCGAACGTCTTTT |
| GJA3-qF | GAGGAAGTCTGGGGAGACGA |
| GJA3-qR | TGAGGGTTGGAGTGGAGACA |
| CHSY3-qF | ACATGCACGACCACTACCTG |
| CHSY3-qR | ACTGCTGTTCAGTGAGCGAA |
| CTSEAL-qF | ATCAGCTTCACCATTGGGCA |
| CTSEAL-qR | CAGAGCGGACCATTGTGAGT |
| MUSTN1-qF | AAAGAAGCGTCCTCCGGTGA |
| MUSTN1-qR | CGGCTGAATATGGAAGGTGC |
| PACSIN1-qF | GGAGGTGGGGAACTACAAGC |
| PACSIN1-qR | ACTGGGGGCCTTTCTCAATG |
| CDKN2B-qF | CGGATGAACTAGCCAACGCC |
| CDKN2B-qR | TCATCACCTGGATGGGGGTC |

**Supplementary Table 4.** Primers and their sequences used in the qRT-PCR experiments of LMH cells stimulated with FST, EPS, and L-Tyr, respectively, in this study. “F” and “R” in the names of the primers indicate “forward” and “reverse” primers, respectively.

| **Primer** | **Sequence (5’–3’)** |
| --- | --- |
| β-Actin-qF | GGGGAAAGTCATCCCTGAGC |
| β-Actin-qR | TTGGCTGGTTTCTCCAGACG |
| FASN-qF | ATCTCCCACCCCTTCTGACT |
| FASN-qR | TCCTCCCACCACCATACCTC |
| SCD1-qF | TGCTGAACATCTGAGGGTGC |
| SCD1-qR | CATTTCCTTGTTGCTGGGGC |
| SREBP1-qF | TCCCGAGGGAGACCATCTAC |
| SREBP1-qR | CTCCAACGCATCCGAAAAGC |
| LXRα-qF | AGTCCCTGGTGGAGAGACTAC |
| LXRα-qR | CCAGATTTCTGAGAGCAGGGG |
| ACCα-qF | ATGAATGGGTACTGCCTGCC |
| ACCα-qR | CCGTCCAGAAACACTGGTCA |
| LPIN1-qF | GGACAGTTAGCAGGGCAAGT |
| LPIN1-qR | CTGGAACGCAGAACTCCCAT |
| PPARα-qF | GGGATGCTGGTAGCCTATGG |
| PPARα-qR | AGACCAGGACGATCTCCACA |
| CPT1A-qF | GGGTTGCCCTTATCGTCACA |
| CPT1A-qR | TACAACATGGGCTTCCGTCC |
| ACOX1-qF | CCAGCACGAGGACTTGAACT |
| ACOX1-qR | TGGGTGAGAAGGGTAGGGAG |

**Supplementary Table 5.** Analysis of fermentation supernatant (FST).

| **Name** | **B.A-TL** | **mz** | **rt** | **exact_mass** | **ppm** | **identify.pvalue** |
| --- | --- | --- | --- | --- | --- | --- |
| 2-Aminoacrylic acid | 90165 | 88.03969121 | 1086.45 | 87.032 | 0.076210774 | 0.010705605 |
| 4-Aminobutyraldehyde | 6981652 | 88.07583674 | 1119.18 | 87.0684 | 1.792914574 | 0.013834426 |
| Butyric acid | 32825579 | 88.07588724 | 766.266 | 88.0524 | 0.546389841 | 1.41E-13 |
| L-lactic acid | 2018770 | 88.9868247 | 45.2652 | 90.0317 | 0.249742904 | 4.87E-42 |
| Hydroxypropionic acid | 78675774 | 89.02309958 | 453.2175 | 90.0317 | 14.81044814 | 0.000497177 |
| Beta-alanine | 3954573 | 89.02341725 | 694.89 | 89.0477 | 1.829355399 | 3.66E-23 |
| Pyruvic acid | 442656670 | 89.50592915 | 1014.095 | 88.016 | 11.83581245 | 0.02316638 |
| Alanine | 182546 | 90.05556435 | 239.12 | 89.0477 | 0.32266382 | 6.63E-17 |
| Dimethylsulfone | 89769371 | 92.92701976 | 86.06915 | 94.0089 | 1.833640705 | 1.46E-24 |
| Aniline | 334768602 | 94.04488036 | 981.8735 | 93.0578 | 6.480786138 | 0.013203988 |
| Phenol | 20689378 | 94.04578729 | 756.71 | 94.0419 | 3.536364941 | 0.040736159 |
| Methylsulfonate | 13507 | 94.97965743 | 89.1297 | 95.98811499 | 12.44011646 | 0.0397982 |
| Sulfate | 21702723 | 98.98441165 | 947.42 | 97.9674 | 0.272045742 | 6.07E-09 |
| Maleic acid | 11969627 | 99.08053588 | 1086.705 | 116.011 | 3.388173348 | 0.041582627 |
| 2-ketobutyric acid | 12750135 | 102.033928 | 614.494 | 102.0317 | 2.493632981 | 3.12E-09 |
| 1-Aminocyclopropanecarboxylic acid | 4960379 | 102.0545621 | 861.26 | 101.0477 | 4.055673666 | 4.08E-13 |
| Cardavillin | 49807171 | 102.0549318 | 126.5465 | 102.1157 | 2.091654644 | 0.015159939 |
| Semialdehyde succinate | 143021827 | 102.0916028 | 996.286 | 102.0317 | 0.032717059 | 3.72E-11 |
| Betaine aldehyde | 40840872 | 102.0919291 | 917.827 | 102.0919 | 0.285037223 | 4.54E-12 |
| Malonic acid esters | 59949721 | 103.0025373 | 78.8769 | 104.011 | 0.180065156 | 1.01E-18 |
| L-serine | 1094619 | 104.0344055 | 86.5501 | 105.0426 | 2.61056778 | 6.51E-15 |
| 2-Hydroxybutyric acid | 868655006 | 104.0531962 | 149.2035 | 104.0473 | 0.070570009 | 8.28E-06 |
| Gamma-aminobutyric acid | 768902434 | 104.1072411 | 89.58175 | 103.0633 | 1.37001063 | 6.60E-07 |
| Hydroxypyruvic acid | 19021820 | 105.0424788 | 1003.125 | 104.011 | 0.021740233 | 1.16E-13 |
| Benzoate | 1302233714 | 105.0430615 | 968.488 | 122.0368 | 3.375007611 | 3.39E-15 |
| 2-Phenylethanol | 2861751 | 105.0700026 | 836.628 | 122.0732 | 0.009004149 | 0.034508327 |
| Benzaldehyde | 116546244 | 107.0492819 | 345.05 | 106.0419 | 0.034009784 | 2.57E-59 |
| Hydroquinone | 5597214164 | 110.0210997 | 1013.485 | 110.0368 | 4.616970394 | 8.99E-13 |
| 2-Aminophenol | 4873038 | 110.0599153 | 1058.36 | 109.0528 | 1.460113789 | 0.008874826 |
| Catechol | 24795385 | 111.0203731 | 648.335 | 110.0368 | 1.419565433 | 1.13E-19 |
| Pyrrole-2-carboxylic acid | 43058735 | 111.5414446 | 984.361 | 111.032 | 6.034668346 | 4.06E-08 |
| Creatinine | 765834 | 112.9526388 | 89.39385 | 113.0589 | 0.521934738 | 0.007566396 |
| Dihydrouracil | 3798174 | 112.9844515 | 880.26 | 114.0429 | 0.001823747 | 0.000138294 |
| Oxalic acid | 32517430 | 112.9853635 | 341.251 | 132.0059 | 7.398410583 | 0.000822333 |
| uracil | 378543293 | 113.0342714 | 147.3655 | 112.0273 | 2.158468011 | 4.17E-23 |
| (S)-1-pyrroline-5-carboxylic acid ester | 11297255 | 114.0551308 | 1135.85 | 113.0477 | 0.092106825 | 0.006253409 |
| 1-pyrroline-2-carboxylic acid | 8060460 | 114.0663216 | 145.147 | 113.0477 | 0.412440222 | 0.001570543 |
| alpha-ketoisovaleric acid | 74323592 | 115.0385178 | 184.857 | 116.0473 | 13.09300597 | 0.034944482 |
| N-methylglycylamide | 19175907 | 115.0502896 | 97.7102 | 114.0429 | 0.559789169 | 0.017898738 |
| Proline | 449652 | 116.0367082 | 98.0404 | 115.0633 | 4.892918059 | 5.99E-16 |
| guanidinoacetic acid | 70072679 | 116.9272367 | 898.746 | 117.0538 | 0.170528575 | 2.34E-26 |
| Methylmalonic acid | 106149589 | 116.927241 | 947.572 | 118.0266 | 0.505337161 | 2.98E-24 |
| Indole | 143890310 | 116.9273897 | 926.805 | 117.0578 | 0.007096327 | 4.63E-72 |
| 5-Aminopentanoic acid | 8715906 | 116.9280076 | 32.1026 | 117.079 | 4.96360316 | 4.84E-11 |
| Succinic acid | 4366952 | 116.9847901 | 79.5695 | 118.0266 | 1.983117043 | 7.10E-24 |
| Valine | 63477886 | 118.0520657 | 105.322 | 117.079 | 0.364793684 | 3.60E-38 |
| Betaine | 2805993 | 118.0565617 | 92.5608 | 117.079 | 7.021828665 | 8.30E-13 |
| Bovinocidin | 1878763 | 119.0162014 | 101.357 | 119.0219 | 2.977629338 | 1.01E-11 |
| L-2,4-diaminobutyric acid | 615070996 | 119.090386 | 142.1255 | 118.0742 | 4.516236248 | 4.45E-06 |
| Threonine | 19925749 | 120.024 | 1137.85 | 119.0582 | 1.063324935 | 0.04092629 |
| Phenylacetaldehyde | 129386276 | 120.0253644 | 983.5175 | 120.0575 | 1.871181638 | 4.66E-05 |
| L-Allallreonine | 5993134 | 120.0655993 | 381.383 | 119.0582 | 0.827047885 | 8.60E-05 |
| 4-Methylbenzaldehyde | 897708971 | 121.0642331 | 193.543 | 120.0575 | 4.484396308 | 4.34E-14 |
| Phenylethylamine | 2820736161 | 121.0843921 | 344.0345 | 121.0891 | 0.03520111 | 4.96E-59 |
| Nicotinic acid | 2944117 | 121.9874403 | 158.553 | 123.032 | 1.952888194 | 1.86E-16 |
| 4-Hydroxybenzaldehyde | 3585040 | 123.0441544 | 631.364 | 122.0368 | 0.560573221 | 1.00E-05 |
| Nicotinamide | 232861352 | 123.0554428 | 157.385 | 122.048 | 1.355486569 | 1.41E-11 |
| 4-Methylbenzyl alcohol | 10121213 | 123.0802382 | 706.262 | 122.0732 | 1.932072959 | 2.23E-08 |
| Pyridinic acid | 23163835 | 124.0869143 | 918.43 | 123.032 | 2.02123794 | 0.000152592 |
| Taurine | 13950618 | 125.9862047 | 44.74115 | 125.0147 | 2.218583191 | 0.029894531 |
| 4-Aminocatechol | 16836313 | 126.0545086 | 1034.27 | 125.0477 | 3.707919734 | 6.06E-05 |
| 1,2,3-Trihydroxybenzene | 189814050 | 127.0397714 | 281.984 | 126.0317 | 3.144505698 | 1.16E-10 |
| Thymine | 988248312 | 127.049986 | 284.923 | 126.0429 | 1.495474387 | 5.76E-09 |
| Imidazoleacetic acid | 14634033 | 127.0500743 | 96.8019 | 126.0429 | 0.760317839 | 0.000562473 |
| D-1-piperidinyl-2-carboxylic acid | 90417953 | 128.069727 | 860.656 | 127.0633 | 6.629201294 | 0.043727026 |
| (R)-5,6-dihydrothymine | 32858985 | 128.0701062 | 286.954 | 128.0586 | 3.955591075 | 7.54E-14 |
| citric acid | 2342913 | 129.0177702 | 348.502 | 130.0266 | 11.85728135 | 0.01248777 |
| pyrrolidone carboxylic acid | 2804063 | 129.1273392 | 179.938 | 129.0426 | 0.508338748 | 2.00E-09 |
| ketoleucine | 188857890 | 130.0075726 | 1000.515 | 130.063 | 1.845277127 | 1.06E-08 |
| 4-oxoproline | 9326980 | 130.0491301 | 691.531 | 129.0426 | 5.735524716 | 3.20E-13 |
| Piperonylic acid | 485747729 | 130.0491632 | 157.098 | 129.079 | 13.84029474 | 2.94E-07 |
| Pyroglutamic acid | 322602162 | 130.0503283 | 179.335 | 129.0426 | 1.741045209 | 2.08E-15 |
| Pyrrolidine-hydroxy carboxylic acid | 1871394 | 130.0507172 | 772.159 | 129.0426 | 3.392367474 | 8.74E-06 |
| 4-Guanylbutyraldehyde | 16682307 | 130.0861782 | 1125.25 | 129.0902 | 0.71069841 | 5.52E-06 |
| L-isoleucine | 4595973 | 130.1259886 | 187.0535 | 131.0946 | 2.320477986 | 6.99E-19 |
| 5-Amino-2-oxovaleric acid | 26514369 | 131.0530366 | 186.085 | 131.0582 | 4.67980262 | 1.86E-09 |
| Guanidine butylamine | 200015905 | 131.1288902 | 88.1977 | 130.1218 | 1.169681996 | 1.35E-27 |
| L-Leucine | 36382608 | 131.9420244 | 213.6125 | 131.0946 | 1.559962491 | 7.32E-26 |
| Ureidopropionic acid | 2009597 | 132.0546536 | 157.385 | 132.0535 | 8.735776957 | 0.006805689 |
| cis-3-Hydroxy-L-proline | 11056272 | 132.0647049 | 1134.65 | 131.0582 | 4.159486719 | 0.006481048 |
| trans-3-hydroxy-L-proline | 76412333 | 132.065294 | 1128.26 | 131.0582 | 1.378106196 | 4.18E-05 |
| 4-Hydroxyproline | 4311076 | 132.0654216 | 63.3851 | 131.0582 | 0.239280348 | 1.88E-05 |
| 3-Methylindole | 303248291 | 132.0805527 | 399.7015 | 131.0735 | 1.296179055 | 6.05E-27 |
| N-carbamoyl putrescine | 487717113 | 132.1021781 | 721.7185 | 131.1059 | 0.159004774 | 1.48E-14 |
| L-Malic acid | 684069 | 132.9722474 | 76.3613 | 134.0215 | 2.548130686 | 1.13E-21 |
| L-ribose | 9663507 | 133.0493099 | 179.335 | 150.0528 | 1.978234379 | 0.000869721 |
| Lipitor | 13239559 | 133.0506908 | 90.558 | 152.0685 | 4.365253548 | 0.017074472 |
| L-asparagine | 4540442 | 133.060562 | 109.475 | 132.0535 | 1.608290216 | 4.71E-06 |
| (2R,4S)-2,4-diaminopentanoate | 4292550 | 133.1022599 | 1133.31 | 132.0899 | 7.045807169 | 0.037661023 |
| L-Aspartic acid | 1741499 | 134.0445164 | 91.8064 | 133.0375 | 1.936670048 | 9.73E-07 |
| Adenine | 1938951 | 136.020397 | 147.603 | 135.0545 | 4.682151928 | 2.47E-16 |
| 4-Hydroxyphenylacetaldehyde | 19115058 | 136.0613276 | 294.531 | 136.0524 | 0.766454126 | 2.85E-08 |
| 2-Phenylacetamide | 3004033 | 136.0754522 | 291.308 | 135.0684 | 1.644675776 | 0.011715922 |
| Hypoxanthine | 46095230 | 137.0029681 | 167.199 | 136.0385 | 3.176142985 | 3.06E-17 |
| 3-Hydroxybenzoic acid | 7186580 | 137.0235422 | 128.5015 | 138.0317 | 4.277795301 | 1.10E-13 |
| 2-Methylbenzoic acid | 41975112 | 137.0593872 | 203.423 | 136.0524 | 1.558667979 | 7.16E-08 |
| 1-Methylnicotinamide | 2547719 | 137.0706265 | 157.051 | 137.0715 | 1.048829174 | 4.84E-08 |
| Phenylacetic acid | 23331845 | 137.1326871 | 668.978 | 136.0524 | 3.302197876 | 3.40E-17 |
| p-Aminobenzoic acid | 131603626 | 138.0543864 | 391.678 | 137.0477 | 4.270780635 | 1.11E-13 |
| Uric acid | 3447168 | 139.0061822 | 146.632 | 138.0429 | 2.832730176 | 2.18E-15 |
| Salicylic acid | 1128339 | 139.1116255 | 659.093 | 138.0317 | 0.602048554 | 0.039337759 |
| Acetyl phosphate | 19252372 | 139.9866686 | 790.516 | 139.9875 | 5.939136979 | 5.78E-09 |
| Imidazole propionic acid | 117721601 | 140.0528579 | 146.632 | 140.0586 | 3.457833694 | 5.20E-19 |
| L-Histidine | 19972059 | 141.0909696 | 602.617 | 141.0902 | 0.04149722 | 0.042375663 |
| o-Phosphoric acid ethanolamine | 23780706 | 141.9575632 | 32.9667 | 141.0191 | 7.861498844 | 3.75E-07 |
| trans-trans-gluconic acid | 120580309 | 143.0331265 | 257.3 | 142.0266 | 4.667627292 | 1.41E-14 |
| Vanilloid | 61377248 | 143.0813275 | 99.5383 | 142.0742 | 0.552261049 | 2.18E-08 |
| 5-(2-hydroxyethyl)-4-methylthiazole | 158905 | 144.046781 | 330.641 | 143.0405 | 6.907478203 | 7.58E-06 |
| 4-oxoglutarate | 25107976 | 145.0497359 | 97.2165 | 145.0375 | 0.010402432 | 9.06E-07 |
| Spermidine | 155458 | 145.9781384 | 66.1878 | 145.1579 | 8.228626712 | 9.00E-09 |
| 2-dehydropantothenic acid ester | 817531403 | 146.0591095 | 399.455 | 146.0579 | 5.816806709 | 1.80E-09 |
| (S)-5-amino-3-oxohexanoate | 8782207 | 146.0805949 | 144.028 | 145.0739 | 3.97792526 | 0.000576382 |
| 2-keto-6-aminohexanoic acid | 16121909 | 146.081014 | 175.996 | 145.0739 | 1.108972452 | 4.79E-07 |
| 4-Guanidinobutyric acid | 254034534 | 146.0924285 | 102.0065 | 145.0851 | 0.359361539 | 1.93E-10 |
| Acetylcholine | 21772315 | 146.1173173 | 101.76 | 146.1181 | 0.316918206 | 1.30E-06 |
| trans-cinnamic | 22069017 | 146.964807 | 683.23 | 148.0524 | 0.250059866 | 1.89E-18 |
| 4-Hydroxycinnamic acid | 88699605 | 146.9804581 | 919.021 | 164.0473 | 3.041994002 | 2.06E-06 |
| L-lysine | 23381026 | 147.0644468 | 79.561 | 146.1055 | 0.372014836 | 8.29E-10 |
| Glutamine | 46655545 | 147.0772277 | 111.793 | 146.0691 | 4.792535616 | 1.58E-05 |
| L-Glutamic acid | 570459071 | 147.114458 | 93.3599 | 147.0532 | 12.97745919 | 2.79E-06 |
| L-2-Hydroxyglutaric acid | 5631031 | 148.0384031 | 40.65055 | 148.0372 | 8.126945271 | 0.031762466 |
| (S)-2-Methylmalic acid | 2255649 | 148.0386052 | 71.5043 | 148.0372 | 5.640116832 | 5.76E-14 |
| o-Acetyl serine | 8639562 | 148.0601232 | 137.782 | 147.0532 | 0.993469111 | 0.000274403 |
| 2-oxo-4-methylthiobutyric acid | 131537040 | 149.0233412 | 258.898 | 148.0194 | 0.014432679 | 7.82E-10 |
| D-xylose | 22138423 | 149.0446793 | 85.21645 | 150.0528 | 0.382918171 | 1.21E-08 |
| L-Methionine | 47104003 | 150.0096115 | 148.929 | 149.051 | 1.515534403 | 2.97E-26 |
| Guanine | 470629568 | 150.0410513 | 187.402 | 151.0494 | 7.149376725 | 9.78E-08 |
| pyridoxal | 86843392 | 150.0517708 | 87.72175 | 167.0582 | 6.696400337 | 0.361964927 |
| Xanthine | 150182535 | 151.0252513 | 188.085 | 152.0334 | 1.318232496 | 2.38E-08 |
| (S)-4-Hydroxymandelic acid ester | 18423205 | 151.0352225 | 73.28435 | 168.0423 | 2.360382138 | 0.105436637 |
| D-xylose | 55279731 | 151.0357747 | 982.129 | 150.0528 | 6.620416447 | 5.34E-09 |
| 3,4-Dihydroxyphenyl glycol | 30354796 | 151.0389036 | 194.583 | 170.0579 | 4.014859652 | 6.68E-06 |
| L-arabinose | 8626866 | 151.0602212 | 1133.45 | 150.0528 | 0.96120606 | 0.008102714 |
| D-ribose | 1338932313 | 151.0614475 | 149.504 | 150.0528 | 0.149156111 | 1.14E-07 |
| 2-Amino-3-phosphonic acid propionic acid | 10734823 | 152.006043 | 146.632 | 169.014 | 3.440652685 | 2.67E-05 |
| Norepinephrine | 22647870 | 152.0703333 | 305.203 | 169.0739 | 11.24294523 | 8.08E-09 |
| N-methyltyramine | 4063723 | 152.1066409 | 303.483 | 151.0997 | 2.20305963 | 4.82E-06 |
| 3-Hydroxyphenylacetic acid | 1015230714 | 152.9948478 | 1020.17 | 152.0473 | 0.333652216 | 3.26E-22 |
| L-arabinitol | 218077861 | 152.9948596 | 1014.55 | 152.0685 | 1.507852948 | 6.59E-18 |
| Diethyl phosphate | 38144609 | 153.0311766 | 177.8005 | 154.0395 | 6.814702881 | 1.19E-08 |
| p-Anisic acid | 7817532 | 153.0544423 | 1077.06 | 152.0473 | 0.038899256 | 3.65E-10 |
| p-Hydroxyphenylacetic acid | 19360884 | 153.06562 | 262.38 | 152.0473 | 1.261722561 | 1.94E-09 |
| Propionyl phosphate | 10349200 | 153.9930051 | 985.28 | 154.0031 | 1.415138102 | 8.04E-09 |
| L-Histidine | 890105727 | 154.0603018 | 95.4143 | 155.0695 | 4.16612761 | 9.72E-13 |
| Gentianic acid | 3605226069 | 154.9900004 | 1015.66 | 154.0266 | 0.119505284 | 1.78E-48 |
| Imidazole-5-acetone pyruvate | 684909 | 155.0421345 | 88.8645 | 154.0378 | 2.428283958 | 2.21E-20 |
| 3-Indoleacetonitrile | 21569994 | 156.1191411 | 772.756 | 156.0687 | 8.849685229 | 5.13E-06 |
| Phosphoglycolic acid | 384697895 | 156.9911347 | 1027.38 | 155.9824 | 9.291607471 | 9.43E-08 |
| L-lactic acid | 4923704 | 157.0281469 | 1012.95 | 156.0171 | 1.281777407 | 0.001395226 |
| Heptanedioic acid | 13839209 | 159.9702009 | 32.9667 | 160.0736 | 6.685184284 | 0.022333109 |
| Indoleacetaldehyde | 1340449961 | 160.0751916 | 332.4405 | 159.0684 | 2.120290194 | 3.18E-23 |
| levulose | 26329267 | 161.0456694 | 499.0285 | 180.0634 | 4.094490727 | 0.011326099 |
| α-D-glucose | 65846592 | 161.0457925 | 297.1 | 180.0634 | 4.858866462 | 0.007935194 |
| O-acetyl-L-homothreonine | 29004626 | 162.0758134 | 109.015 | 161.0688 | 0.819394518 | 3.72E-12 |
| Tryptophan | 21075277 | 162.0913015 | 611.736 | 161.0841 | 0.026027633 | 2.16E-14 |
| Rhamnose furanose | 21573171 | 164.0666364 | 982.129 | 164.0685 | 1.039820245 | 2.47E-07 |
| Phthalic acid | 3868421 | 165.040415 | 887.855 | 166.0266 | 1.80663429 | 6.76E-08 |
| Formaldehyde-benzoic acid | 6041001 | 166.0496067 | 364.643 | 165.0426 | 0.916660605 | 4.78E-08 |
| L-Methionine S-oxide | 237177708 | 166.0531598 | 138.376 | 165.046 | 0.001011224 | 2.88E-16 |
| D-Phenyl lactic acid | 37476205 | 166.0588871 | 190.017 | 166.063 | 5.717718254 | 1.23E-12 |
| Norepinephrine | 4189924 | 166.0846327 | 111.793 | 183.0895 | 2.557026939 | 0.035048696 |
| L-Phenylalanine | 4820059255 | 166.0857776 | 381.8785 | 165.079 | 3.000858997 | 1.59E-07 |
| Barley base | 68308160 | 166.1205694 | 171.391 | 165.1154 | 12.22365182 | 0.000377631 |
| Vanillic acid | 487039 | 169.0491062 | 1087.05 | 168.0423 | 2.779074143 | 0.016790721 |
| Uric acid | 9657392 | 169.0854607 | 645.336 | 168.0283 | 2.007688281 | 0.015410061 |
| Pyridoxamine (vitamin B6) | 120046487 | 169.0974217 | 364.374 | 168.0899 | 1.453008553 | 7.71E-12 |
| 1-Methylhistidine | 171853270 | 169.9771548 | 1126.05 | 169.0851 | 1.811823215 | 0.0045545 |
| Pyridoxine vitamin B7 | 216280908 | 170.0803473 | 171.919 | 169.0739 | 3.525664532 | 7.79E-19 |
| Rac-glycerol 3-phosphate | 174447634 | 171.0055584 | 81.26905 | 172.0136746 | 4.9135128 | 0.014080979 |
| Decanoic acid | 395877 | 171.1380375 | 808.406 | 172.1463 | 5.76435265 | 4.65E-06 |
| 1-Hydroxy-2-naphthoic acid ester | 3407125 | 171.1485929 | 38.5402 | 188.0473 | 1.67751822 | 0.043039047 |
| L-Arginine | 1461225 | 173.1038832 | 92.5137 | 174.1117 | 1.770843255 | 1.76E-11 |
| N-Acetyl-L-glutamic acid 5-semialdehyde | 1161650 | 174.0759977 | 906.962 | 173.0688 | 0.449803338 | 0.000196473 |
| 1H-indole-3-acetamide | 85237602 | 175.0863529 | 396.435 | 174.0793 | 1.274228381 | 0.014049579 |
| N-Formyl-L-methionine | 44293186 | 176.0379134 | 205.664 | 177.046 | 2.400365153 | 2.90E-14 |
| N-Formyl-L-glutamic acid | 24887156 | 176.0702664 | 545.984 | 175.0481 | 0.792925562 | 0.000432783 |
| Citrulline | 18498682 | 176.102339 | 92.8104 | 175.0957 | 2.262472834 | 0.006298282 |
| Pyrophosphate | 27108786 | 176.9353171 | 81.5697 | 177.9432 | 3.294424254 | 0.009027779 |
| Ascorbic acid | 17458700 | 176.9716507 | 1136.675 | 176.0321 | 2.578003834 | 0.013053526 |
| 2-Dehydro-3-Deoxy-L-Galactonate | 2160392 | 177.0393532 | 213.537 | 178.0477 | 6.04833617 | 0.055441134 |
| L-Galactose-1,4-lactone | 20530243 | 177.0394276 | 612.711 | 178.0477 | 5.628093164 | 0.1099365 |
| L-Gulonolactone | 91307605 | 177.0399938 | 861.8495 | 178.0477 | 2.429953512 | 0.002461693 |
| 2-Hydroxy-3-oxoadipate | 1020470 | 177.0545477 | 1132.54 | 176.0321 | 0.229408798 | 0.016087933 |
| D-Mannose | 14165864 | 179.0554789 | 957.977 | 180.0634 | 3.468757303 | 0.037544727 |
| D-Glucose | 217919614 | 179.0568074 | 84.93315 | 180.0634 | 3.816665839 | 0.00060114 |
| 4-Hydroxyphenylpyruvic acid | 10022732 | 180.0439965 | 1043.28 | 180.0423 | 1.661140937 | 3.25E-05 |
| L-fucose | 6657542 | 180.9718607 | 747.3715 | 164.0685 | 8.021567134 | 9.31E-08 |
| Galactitol | 43354786 | 180.9724311 | 89.87575 | 182.079 | 3.942209571 | 4.01E-05 |
| Inositol | 345645916 | 180.989257 | 1017.99 | 180.0634 | 0.594643156 | 4.64E-08 |
| D-Galactose | 54786493 | 181.013032 | 822.406 | 180.0634 | 4.799415967 | 4.20E-09 |
| D-fructose | 48204822 | 181.0149209 | 806.309 | 180.0634 | 4.846686365 | 3.01E-11 |
| 1D-Chiro-inositol | 3428971 | 181.0855881 | 762.693 | 180.0634 | 0.834057219 | 0.000928763 |
| Tyrosine | 21993236783 | 182.0799599 | 203.1675 | 181.0739 | 6.678933808 | 3.91E-12 |
| Sorbitol | 5412071818 | 182.9849359 | 1017.99 | 182.079 | 0.219940083 | 5.48E-12 |
| Selenomethoxycysteine | 1749610601 | 182.9849384 | 1125.745 | 182.9799 | 1.779124429 | 0.013272902 |
| 4-Pyruvic acid | 35472209 | 182.9871015 | 45.9017 | 183.0532 | 7.516096652 | 6.14E-12 |
| Homovanillic acid | 1667207 | 183.0649126 | 1006.87 | 182.0579 | 1.438833888 | 1.23E-19 |
| Choline sulfate | 41206698 | 184.0577856 | 111.011 | 183.0565 | 0.781612283 | 0.018806146 |
| Hydroxypyruvic acid phosphate | 24742506 | 184.9850229 | 842.598 | 183.9773 | 2.415871258 | 0.00061192 |
| Phosphoric acid serine | 1106680 | 185.0231145 | 48.9107 | 185.0089 | 0.995520162 | 0.038572156 |
| (Z)-but-1-ene-1,2,4-tricarboxylate | 89252654 | 188.0336611 | 137.224 | 188.0321 | 8.30230583 | 0.419728224 |
| Uric acid | 34489481 | 188.0339658 | 530.807 | 189.0426 | 2.846099399 | 3.32E-12 |
| N-Acetyl glutamic acid | 59578438 | 188.0559644 | 82.59035 | 189.0637 | 1.60760824 | 8.71E-26 |
| N6-acetyl-L-lysine | 286287598 | 189.1226773 | 147.6505 | 188.1161 | 2.481948599 | 6.52E-17 |
| Diaminoheptanedioic acid | 77557292 | 190.1058522 | 292.812 | 190.0954 | 7.057316818 | 0.017300245 |
| Citric acid | 3189051 | 190.9496471 | 73.5317 | 192.027 | 3.921371845 | 1.03E-14 |
| Isocitric acid | 15208606 | 191.0190542 | 73.6599 | 192.027 | 3.506444183 | 0.005002366 |
| 5-Hydroxyindoleacetic acid | 58361265 | 192.0649239 | 339.61 | 191.0582 | 0.319330322 | 0.000179374 |
| D-glucuronide | 1067241 | 193.0370267 | 626.267 | 194.0427 | 8.302621181 | 0.000589496 |
| Deoxyribose 5-phosphate | 442051 | 195.0062101 | 85.3636 | 214.0242392 | 1.850617987 | 0.041563644 |
| Glucuronic acid | 13230111 | 195.0505186 | 84.3829 | 196.0583 | 0.00418992 | 0.000159656 |
| Doba | 6407527 | 197.0943846 | 864.484 | 197.0688 | 10.56074965 | 0.02517415 |
| D-erythrose 4 phosphate | 23569492 | 199.0004895 | 76.84865 | 200.0086 | 4.072854303 | 0.010787872 |
| Vanillic acid | 24464576 | 199.0265495 | 1031.31 | 198.0528 | 12.96072472 | 6.64E-07 |
| γ-glutamine-β-aminopropionitrile | 2508179 | 199.106194 | 1061.82 | 199.0957 | 7.009936014 | 0.022610248 |
| N-Acetyl 5-hydroxytryptamine | 590305 | 199.1699756 | 882.8915 | 218.1055 | 4.043445929 | 0.000194643 |
| Cysteine sulfate | 5893133 | 199.9687074 | 84.3829 | 200.9766 | 2.963463672 | 0.009000596 |
| Dodecanoic acid | 100488233 | 199.9854319 | 1083.755 | 200.1776 | 9.251103595 | 3.27E-10 |
| L-tryptophan | 1023271560 | 203.0819938 | 379.378 | 204.0899 | 0.551538912 | 4.30E-35 |
| Spermine | 5126561 | 203.1439445 | 64.4749 | 202.2157 | 4.472746309 | 2.94E-15 |
| Indole pyruvate | 98167178 | 204.0624934 | 214.019 | 203.0582 | 4.82887576 | 0.012611022 |
| N6-acetyl-N6-hydroxy-L-lysine | 109045114 | 204.1232355 | 477.596 | 204.111 | 1.29019891 | 4.50E-14 |
| N-Acetyl-L-phenylalanine | 41760655 | 206.0814991 | 393.993 | 207.0895 | 0.068277395 | 2.38E-53 |
| Homocitric acid | 165353702 | 207.0502927 | 84.2521 | 206.0427 | 1.529582404 | 0.071512152 |
| L-3-hydroxy-adrenaline | 8423637 | 207.0737306 | 79.7384 | 224.0797 | 7.072893093 | 0.130047418 |
| L-uracil | 339914430 | 209.0920137 | 339.363 | 208.0848 | 0.297954948 | 2.12E-19 |
| Jasmonic acid | 26302764 | 211.1309017 | 627.381 | 210.1256 | 8.058431733 | 0.002577659 |
| Desulfobiotin | 482743637 | 213.9824196 | 1011.01 | 214.1317 | 0.153248936 | 3.76E-14 |
| (-)-su-iso-(homo)3-citrate | 362276 | 217.0683319 | 330.156 | 234.074 | 8.135292471 | 0.148888225 |
| β-Alanyl-L-lysine | 5560315 | 218.1574932 | 330.978 | 217.1426 | 3.067264719 | 2.33E-12 |
| 5-Hydroxy-L-tryptophan | 3620731 | 219.0767158 | 338.437 | 220.0848 | 0.814421566 | 2.48E-10 |
| Pantothenic acid | 64771014 | 219.1061051 | 190.008 | 219.1107 | 0.441331724 | 2.31E-19 |
| 1D-1-guanidino-3-amino-1,3-dideoxyhexylinositol | 91917695 | 221.1208288 | 367.308 | 220.1172 | 1.829945497 | 0.000911783 |
| L-cystine | 4073697 | 221.1541717 | 864.0575 | 222.0674 | 0.693713986 | 1.94E-07 |
| Pyrimidinediazepine | 151049732 | 222.095385 | 298.939 | 221.0913 | 14.36770062 | 1.22E-05 |
| Thymidine | 2659735 | 223.0280695 | 766.385 | 242.0903 | 0.794147344 | 7.36E-09 |
| Methyl jasmonate | 323641635 | 224.0113773 | 1017.735 | 224.1412 | 0.884545193 | 1.92E-30 |
| Myostatin | 1285560 | 225.099352 | 97.3817 | 226.1066 | 0.124389519 | 0.022882532 |
| Benzoate | 16512813 | 226.0670391 | 282.682 | 226.0477 | 6.449607841 | 0.006582018 |
| Cholestyramine | 21648639 | 226.1799362 | 700.032 | 226.0954 | 2.408511737 | 1.20E-11 |
| Myristic acid | 5897799 | 227.2014371 | 950.7575 | 228.2089 | 0.771752451 | 2.27E-07 |
| Deoxyuridine | 66103102 | 228.1955397 | 887.089 | 228.0746 | 0.721440464 | 7.81E-11 |
| Traumatic acid | 45789605 | 229.1420209 | 627.381 | 228.1361591 | 4.362926883 | 0.001668415 |
| N-Acetyl-L-2-amino-6-oxime acid ester | 20731142 | 231.0968646 | 152.55 | 231.0743 | 0.040632369 | 0.000242503 |
| N2-succinyl-L-glutamic acid 5-semialdehyde | 13599003 | 231.096895 | 230.597 | 231.0743 | 1.754684718 | 3.02E-16 |
| Deoxyinosine | 9094851 | 233.1548263 | 869.929 | 252.0859 | 2.336897853 | 2.24E-11 |
| Luminescent chromium | 8514489 | 241.0728296 | 573.755 | 242.0804 | 1.121652741 | 0.021936876 |
| Pseudouridine | 45561474 | 243.0621214 | 153.519 | 244.0695 | 0.422114311 | 2.09E-09 |
| Uridine | 24249103 | 243.9412884 | 968.7915 | 244.0695 | 3.735763786 | 2.38E-11 |
| γ-glutamyl-β-cyanoalanine | 52580660 | 244.0922924 | 102.0065 | 243.0855 | 1.687461869 | 0.011309901 |
| Cytidine | 1730671 | 244.0963398 | 458.715 | 243.0855 | 11.81080364 | 0.015727817 |
| β-alanyl-L-arginine | 4150631 | 246.1553007 | 162.78 | 245.1488 | 3.149637639 | 0.000552615 |
| D-Octopamine | 36441934 | 247.1398402 | 147.644 | 246.1328 | 0.024592304 | 8.13E-08 |
| S-ribosyl-L-homocysteine | 199889609 | 248.054985 | 175.971 | 267.0777 | 2.938855906 | 0.029776274 |
| Pyridamine 5-phosphate | 41354623 | 248.0758691 | 265.7105 | 248.0562 | 1.648880971 | 1.23E-15 |
| γ-Glutamylcysteine | 94818504 | 248.9605239 | 89.6353 | 250.0623 | 1.335869658 | 2.38E-25 |
| Xanthine | 12668025 | 251.1635957 | 695.699 | 250.1569 | 1.538740469 | 7.16E-05 |
| Palmitoleic acid | 34039428 | 254.2472838 | 920.2285 | 254.2246 | 0.219560273 | 1.18E-08 |
| Nicotinamide riboside | 397088295 | 255.097233 | 101.357 | 255.0981 | 0.096087714 | 8.48E-09 |
| Palmitic acid | 11756836 | 255.2312442 | 889.593 | 256.2402 | 5.451127472 | 4.05E-06 |
| Glycerophosphorylcholine | 27971878 | 258.1084795 | 90.8192 | 258.1106 | 4.928454577 | 2.59E-09 |
| Linolenic acid | 155767441 | 259.0914173 | 250.0155 | 259.1168 | 2.75187627 | 2.11E-18 |
| Stearic acid | 8448623 | 265.1475303 | 773.97 | 284.2715 | 1.582820279 | 1.12E-06 |
| Theophylline | 38704193 | 265.1535657 | 441.155 | 264.1474 | 4.18738476 | 2.65E-17 |
| Adenosine | 83460 | 266.0887689 | 348.999 | 267.0968 | 2.747579325 | 0.000327442 |
| Homocysteine | 20835190 | 267.0472892 | 100.7 | 268.0551 | 2.00264156 | 5.66E-07 |
| 2-Deoxyguanosine | 19202057 | 267.1716978 | 863.7825 | 267.097 | 2.383933924 | 5.42E-05 |
| Inosine | 61800965 | 268.1029993 | 294.8295 | 268.0808 | 5.129744925 | 9.64E-11 |
| (2R,3R)-3-Methylglutamyl-5-semialdehyde N6-lysine | 204615341 | 274.1861005 | 97.79355 | 273.1689 | 3.65726593 | 0.000351527 |
| (2R,3R)-3-Methylpyridinylmethyl-N6-lysine | 5680502 | 275.2070693 | 75.40915 | 274.2005 | 1.968641232 | 5.79E-24 |
| N-acetylmuramic acid | 104111997 | 276.1067698 | 350.21 | 293.1111 | 11.24130351 | 3.84E-06 |
| 4-Hydroxycinnamic acid | 266739512 | 276.1438461 | 280.7915 | 276.1586 | 0.540288509 | 2.59E-11 |
| 6-Phosphogluconic acid | 15963826 | 277.1792514 | 879.3225 | 276.0246 | 2.894556263 | 1.06E-09 |
| Oleic acid | 21097555 | 281.2490636 | 921.873 | 282.2559 | 0.98949549 | 1.97E-07 |
| Sphingosine | 10916521 | 282.2791771 | 884.181 | 299.2824 | 14.91820985 | 0.000568877 |
| Guanosine | 30074485 | 284.0982623 | 309.2425 | 283.0917 | 2.512158977 | 4.43E-06 |
| Phthalic acid esters | 2465162 | 290.1346865 | 115.329 | 289.1274 | 0.036190088 | 4.88E-09 |
| Deoxyxanthine Furtose | 2303414 | 296.0688176 | 90.60125 | 296.0896 | 8.584871867 | 4.79E-08 |
| 5-Methylthioadenosine | 990808 | 297.2441339 | 872.6775 | 297.0896 | 2.544085372 | 5.95E-07 |
| D-4-phosphopantothenate | 717585 | 299.1001222 | 391.292 | 299.077 | 0.73419186 | 4.86E-11 |
| 15-deoxy-D-12,14-Pgj2 | 11393341 | 299.1965347 | 718.353 | 316.2038 | 0.563843429 | 0.00314038 |
| Sphingosine | 551121899 | 302.3050965 | 768.2585 | 301.2981 | 0.924562646 | 9.31E-05 |
| 12(S)-Hete | 12176261 | 303.2317897 | 861.975 | 320.2351 | 0.499897086 | 5.96E-10 |
| Arachidonic acid | 5652587 | 303.233025 | 32.1026 | 304.2402 | 0.082444846 | 0.017069863 |
| Glutathione | 24381 | 308.0901891 | 132.614 | 307.0838 | 1.65127882 | 1.11E-07 |
| Ribose 1,5-bisphosphate | 4359411 | 309.1715062 | 797.86 | 309.9855 | 8.497228006 | 6.55E-08 |
| Dapdiamide B | 74146263 | 314.1579489 | 317.5395 | 314.159 | 3.34575804 | 0.214755248 |
| Union | 9450023 | 323.0279152 | 101.794 | 324.0359 | 2.119940624 | 0.000233395 |
| Sucrose | 1847526 | 323.1717241 | 899.826 | 342.1162 | 3.121461111 | 1.98E-07 |
| Glycerol phospholipids | 1118116 | 323.2205141 | 897.539 | 323.0519 | 0.207148634 | 1.73E-05 |
| 3ˊ,5ˊ-cyclic adenosine acid | 170829 | 330.0585354 | 220.907 | 329.0525 | 3.758727216 | 5.71E-10 |
| 12-keto-tetrahydro-leukotriene B4 | 10019839 | 336.3096994 | 951.964 | 336.2301 | 0.264447597 | 3.12E-17 |
| 8,9-diHETRE | 234928 | 338.249639 | 872.243 | 338.2457 | 11.64524524 | 1.96E-06 |
| Fructose1,6-diphosphate | 53370686 | 339.1996458 | 829.497 | 339.996 | 0.271277509 | 1.58E-05 |
| Alginose | 2512932 | 341.1078911 | 94.9388 | 342.1162 | 2.159763946 | 2.66E-21 |
| Maltose | 2006023 | 341.1091242 | 102.331 | 342.1162 | 0.586909301 | 0.128710794 |
| Guanosine 2,3-cyclic phosphate | 60891 | 346.0544611 | 248.415 | 345.0474 | 0.621000133 | 0.051820918 |
| 3-Adenosine monophosphate | 35249373 | 346.0561404 | 321.353 | 347.0631 | 0.914302517 | 4.19E-08 |
| Circulating GMP | 164235763 | 346.3306696 | 769.1655 | 345.0474 | 1.692831899 | 3.36E-06 |
| dGMP | 12950857 | 347.1702862 | 900.434 | 347.0631 | 1.054103169 | 3.94E-06 |
| Ampicillin | 232685717 | 348.0692857 | 299.9535 | 347.0631 | 0.081807609 | 0.000419359 |
| Rosmarinic acid | 6073112 | 360.1305936 | 558.4655 | 360.0845 | 1.803988505 | 5.76E-08 |
| Cholic acid | 47208387 | 376.3115414 | 802.3045 | 376.2977 | 2.081447885 | 4.30E-14 |
| Tocopherol delta | 18579619 | 385.2909647 | 951.964 | 402.3498 | 3.756119981 | 1.24E-09 |
| Deoxycholic acid | 83188962 | 393.2834566 | 804.0385 | 392.2927 | 4.17015442 | 3.21E-09 |
| S-adenosylmethionine | 227387128 | 398.2400777 | 804.3445 | 398.137 | 1.866812872 | 2.00E-06 |
| Lenpropionic acid | 7236915 | 409.1642452 | 805.398 | 408.2876 | 7.644999578 | 2.14E-07 |
| Glycolic acid | 5401936 | 465.3022368 | 890.544 | 465.309 | 4.969697906 | 6.98E-05 |
| Adenosine triphosphate | 3106419 | 507.1185885 | 938.486 | 506.9957 | 5.293852949 | 3.40E-14 |
| Biotin-5-AMP | 115411 | 574.1330912 | 327.344 | 573.1407 | 5.58822954 | 8.89E-15 |
| 3-Hydroxyethyl bacteriochlorophyllin A | 15305779 | 633.2537929 | 337.274 | 634.2642 | 4.944438633 | 0.118305822 |
| 3-Methylcrotonyl coenzyme A | 5769249 | 851.4027854 | 810.303 | 849.1571 | 5.680250679 | 1.40E-07 |

**Supplementary Table 6.** Analysis of exopolysaccharides (EPS).

| **Name** | **Content (%)** |
| --- | --- |
| Mannose | 59.75% |
| Glucose | 33.00% |
| Galactose | 5.51% |
| Arabinose | 0.97% |
| Fucose | 0.54% |
| Rhamnose | 0.22% |

**Supplementary Table 7.** Summary of sequencing data for each sample.

| **Sample name** | **Raw reads** | **Clean reads** | **Error rate(%)** | **Q30 (%)** | **Mapped reads** | **Multiple mapped** | **Uniquely mapped** | **CDS** | **Intergenic** | **Introns** | **3'UTR** | **5'UTR** |
| --- | --- | --- | --- | --- | --- | --- | --- | --- | --- | --- | --- | --- |
| Con1 | 52320542 | 51792170 | 0.0241 | 95.17 | 41162931 | 1760654 | 39402277 | 60005354 | 789860 | 1160162 | 7699730 | 2000930 |
| Con2 | 60585418 | 59985926 | 0.0238 | 95.4 | 47295904 | 1975528 | 45320376 | 70978511 | 797354 | 1112544 | 7989697 | 2157754 |
| Con3 | 48249016 | 47761320 | 0.0241 | 95.11 | 38799635 | 1545564 | 37254071 | 58321472 | 715060 | 926655 | 6680532 | 1657530 |
| Con4 | 51719664 | 51232208 | 0.0239 | 95.36 | 41400598 | 1749195 | 39651403 | 61642444 | 734239 | 1163255 | 7200611 | 1960533 |
| Con5 | 61752888 | 61188544 | 0.0239 | 95.39 | 49766753 | 2246780 | 47519973 | 74820920 | 872331 | 1228688 | 8408067 | 2352637 |
| Con6 | 53114876 | 52638910 | 0.0238 | 95.42 | 43281515 | 1843014 | 41438501 | 64914972 | 756791 | 980492 | 7518981 | 2008333 |
| TL1 | 59264500 | 58702080 | 0.0239 | 95.38 | 46153556 | 1999441 | 44154115 | 68932809 | 791647 | 1040026 | 8369463 | 2139173 |
| TL2 | 58655488 | 58134554 | 0.0236 | 95.59 | 47356211 | 2120528 | 45235683 | 70557521 | 882384 | 1106027 | 8452332 | 2123216 |
| TL3 | 51622024 | 51171222 | 0.024 | 95.24 | 41687604 | 1566104 | 40121500 | 63340829 | 731490 | 955414 | 7321623 | 1932999 |
| TL4 | 54028528 | 53545614 | 0.0238 | 95.43 | 44325483 | 1820473 | 42505010 | 66057580 | 819669 | 1112938 | 8027723 | 2116401 |
| TL5 | 58379288 | 57752746 | 0.0239 | 95.3 | 46981136 | 2077807 | 44903329 | 71893413 | 792844 | 940304 | 7895273 | 2367491 |

**Supplementary Table 8.** All DEGs between the control and experimental groups.

| **Gene name** | **Log_2_FC(TL/Con)** | **Pvalue** | **Regulate** | **Con** | **TL** |
| --- | --- | --- | --- | --- | --- |
| ANO8 | -1.520472575 | 5.18387E-10 | down | 9.75 | 3.032 |
| LOC121107598 | 2.876313655 | 1.05208E-09 | up | 0.616666667 | 4.668 |
| LYZ | -1.656240656 | 3.82411E-09 | down | 23.95833333 | 6.88 |
| FASN | 1.505907192 | 3.96927E-09 | up | 56.58833333 | 142.304 |
| FAM222A | -1.418779063 | 3.89118E-08 | down | 2.215 | 0.876 |
| MT3 | -1.198978476 | 2.26723E-07 | down | 918.7366667 | 389.884 |
| ABCC5 | -1.073809492 | 2.61785E-07 | down | 16.38666667 | 7.148 |
| ANO1 | -1.656844864 | 3.58396E-07 | down | 22.94666667 | 6.66 |
| CADM3 | -1.028959174 | 2.06099E-06 | down | 16.99833333 | 7.354 |
| TK1 | 1.646310951 | 2.30274E-06 | up | 2.428333333 | 6.44 |
| GBP1 | -1.013427714 | 2.56132E-06 | down | 63.565 | 28.158 |
| RIMBP2 | 1.275864847 | 2.87468E-06 | up | 0.283333333 | 0.61 |
| AP3B2 | -1.265414748 | 3.44403E-06 | down | 0.883333333 | 0.332 |
| P2RX7 | -1.010381156 | 5.43423E-06 | down | 6.726666667 | 2.774 |
| ACAT2 | 1.202842053 | 5.51323E-06 | up | 261.2516667 | 538.222 |
| RSFR | -1.126158898 | 5.92618E-06 | down | 216.5566667 | 88.092 |
| LOC107049924 | -1.725282546 | 6.28904E-06 | down | 2.523333333 | 0.688 |
| LOC100857706 | -1.013517706 | 8.5915E-06 | down | 6.203333333 | 2.724 |
| LOC101750889 | -1.148264448 | 9.56092E-06 | down | 53.705 | 21.346 |
| ADD2 | -1.14265667 | 1.16856E-05 | down | 1.408333333 | 0.564 |
| HSP90AA1 | 1.08038493 | 1.30448E-05 | up | 286.5233333 | 540.838 |
| BUB1 | -1.424098317 | 1.50673E-05 | down | 1.971666667 | 0.66 |
| PRAP1 | 1.442643245 | 1.51816E-05 | up | 14.48166667 | 35.208 |
| ELF3 | -1.396159114 | 2.06741E-05 | down | 4.316666667 | 1.468 |
| MXD3 | -1.04019265 | 2.32955E-05 | down | 7.838333333 | 3.372 |
| LOC107055472 | 2.633322905 | 2.91365E-05 | up | 0.411666667 | 2.372 |
| LOC121110596 | 1.182394426 | 3.12971E-05 | up | 0.425 | 0.852 |
| MSMB | -2.27048754 | 4.81357E-05 | down | 7.995 | 1.384 |
| HSPH1 | 1.006506532 | 5.36882E-05 | up | 4.89 | 8.808 |
| LOC107050473 | -2.619403005 | 6.41289E-05 | down | 2.103333333 | 0.46 |
| LOC121112262 | -1.75608213 | 6.54232E-05 | down | 7.445 | 1.814 |
| RIMS2 | -1.316692873 | 7.39952E-05 | down | 0.32 | 0.136 |
| IFIT5 | -1.362614599 | 8.18338E-05 | down | 29.66 | 10.43 |
| LOC107049652 | -1.731021961 | 8.78506E-05 | down | 3.798333333 | 1.002 |
| SAMD9L | -1.025926678 | 9.34054E-05 | down | 1.408333333 | 0.62 |
| LPIN1 | 2.171768002 | 9.90447E-05 | up | 8.636666667 | 35.824 |
| LOC112530214 | -1.212574544 | 0.000109398 | down | 0.323333333 | 0.122 |
| UBE2L6 | -1.026272775 | 0.000117011 | down | 59.93 | 26.258 |
| CCDC57 | 2.130281794 | 0.000117887 | up | 0.785 | 3.11 |
| LOC112531157 | -1.931410116 | 0.000123596 | down | 0.738333333 | 0.17 |
| CYP2W1 | -1.728488629 | 0.000131174 | down | 6.873333333 | 2.066 |
| LOC107054120 | -1.39514626 | 0.00013134 | down | 0.333333333 | 0.122 |
| LOC100858381 | -1.493502949 | 0.000143952 | down | 3.603333333 | 1.152 |
| GRIA1 | -2.591225183 | 0.000166354 | down | 0.32 | 0.048 |
| CKS1B | 1.188763579 | 0.000177953 | up | 14.71166667 | 29.788 |
| PMLL | -1.015599708 | 0.000178435 | down | 9.478333333 | 4.27 |
| USP41 | -1.41601782 | 0.000183957 | down | 23.895 | 8.156 |
| LOC422513 | -1.085937216 | 0.000203675 | down | 28.80166667 | 13.46 |
| GPR156 | 1.33355109 | 0.000210596 | up | 7.42 | 16.82 |
| BTBD11 | -1.225529745 | 0.000214788 | down | 0.263333333 | 0.098 |
| H2AFJ | 1.649235981 | 0.000230132 | up | 11.76666667 | 33.538 |
| GLOD5 | 1.25669341 | 0.00023301 | up | 0.853333333 | 1.822 |
| LOC121109112 | 2.878224374 | 0.00024451 | up | 0.151666667 | 1.002 |
| PLEKHH1 | -1.308679775 | 0.000245215 | down | 0.668333333 | 0.242 |
| LOC112532760 | 2.254161882 | 0.000261113 | up | 0.028333333 | 0.116 |
| TPPP | 1.144976935 | 0.000271123 | up | 1.075 | 2.116 |
| PPP1R3G | 1.464271483 | 0.000294957 | up | 4.876666667 | 12.112 |
| MID1IP1 | -1.205082176 | 0.000312605 | down | 58.99166667 | 22.9 |
| LOC107049798 | -1.102392849 | 0.000342353 | down | 2.946666667 | 1.23 |
| LOC121108679 | 2.007597047 | 0.000385718 | up | 1.803333333 | 6.348 |
| LOC107055361 | -1.039870893 | 0.000404883 | down | 608.5 | 265.62 |
| BHLHE41 | -1.046813315 | 0.000412206 | down | 6.581666667 | 2.802 |
| CEP128 | 2.29936809 | 0.000416144 | up | 0.496666667 | 4.074 |
| LOC107052215 | -3.227817645 | 0.000419796 | down | 0.208333333 | 0.02 |
| LOC121112833 | -2.274851864 | 0.000449928 | down | 5.436666667 | 0.99 |
| TGM4 | 1.385133621 | 0.000452106 | up | 2.365 | 5.526 |
| INSIG1 | 1.082065759 | 0.00047428 | up | 47.60833333 | 89.742 |
| DIO3 | 4.204322255 | 0.000486221 | up | 0.16 | 2.594 |
| LOC121107894 | -1.012615683 | 0.000499477 | down | 8.871666667 | 3.892 |
| LOC101748511 | 3.509330755 | 0.000545346 | up | 0.01 | 0.094 |
| FGF1 | -1.106154296 | 0.000547033 | down | 2.216666667 | 0.93 |
| LOC101751538 | -2.40654968 | 0.000557148 | down | 0.205 | 0.036 |
| LOC121109177 | -1.464008683 | 0.00060528 | down | 1.241666667 | 0.376 |
| MMP7 | -2.675561143 | 0.000630291 | down | 2.513333333 | 0.346 |
| OASL | -2.002187642 | 0.000631466 | down | 209.93 | 46.334 |
| LOC121109462 | -1.96178024 | 0.000695899 | down | 6.968333333 | 1.46 |
| C11orf74 | 1.024811657 | 0.000705533 | up | 5.543333333 | 10.234 |
| LOC121112306 | -1.125387999 | 0.000811145 | down | 4.368333333 | 1.858 |
| RRM2 | 1.28802986 | 0.000826383 | up | 1.321666667 | 2.844 |
| CDHR2 | 1.628196326 | 0.000830737 | up | 0.705 | 1.93 |
| FAM155B | -1.348632625 | 0.000887469 | down | 0.25 | 0.09 |
| LOC101748113 | -3.357248039 | 0.000915684 | down | 0.131666667 | 0.012 |
| CDKN1A | 1.323709941 | 0.000925732 | up | 2.578333333 | 5.12 |
| LOC121106538 | 1.461493191 | 0.000928258 | up | 14.57 | 35.886 |
| LOC107057173 | 1.706707866 | 0.000964651 | up | 4.003333333 | 11.792 |
| GDF7 | -2.853373846 | 0.001044411 | down | 0.09 | 0.01 |
| FBN3 | -1.949178959 | 0.00110405 | down | 0.056666667 | 0.012 |
| ST6GALNAC2 | -1.086505042 | 0.00114175 | down | 5.176666667 | 2.072 |
| MX1 | -1.736511352 | 0.001205119 | down | 10.30833333 | 2.692 |
| LOC121108794 | 4.320199758 | 0.001346912 | up | 0.008333333 | 0.14 |
| LOC107050230 | -1.075500397 | 0.001404617 | down | 8.311666667 | 3.498 |
| UCHL1 | 1.871980538 | 0.001437571 | up | 1.313333333 | 4.288 |
| LOC107053391 | -1.801774779 | 0.001446359 | down | 0.808333333 | 0.206 |
| LOC121110112 | 1.10626703 | 0.001460173 | up | 0.353333333 | 0.672 |
| STEAP4 | -1.039669926 | 0.001597432 | down | 6.11 | 2.682 |
| RSAD2 | -1.369180748 | 0.001624667 | down | 4.295 | 1.504 |
| LOC107052698 | -1.62802032 | 0.001689193 | down | 2.263333333 | 0.638 |
| SLC19A2 | -1.002431325 | 0.001723533 | down | 11.03166667 | 4.9 |
| ALPI | 1.90955948 | 0.001729289 | up | 2.273333333 | 7.864 |
| LOC107053912 | 3.133758068 | 0.001941935 | up | 0.01 | 0.076 |
| AVPI1 | 1.143350252 | 0.002135268 | up | 7.235 | 14.42 |
| MTHFD2 | -1.169295802 | 0.002218006 | down | 12.33166667 | 4.86 |
| LYGL | -1.474559212 | 0.002222397 | down | 3.628333333 | 1.17 |
| FAM150B | -1.426818629 | 0.002245749 | down | 3.916666667 | 1.27 |
| PCK1 | 1.130584855 | 0.002363294 | up | 320.5366667 | 626.38 |
| LOC777017 | -1.025712368 | 0.002372809 | down | 5.961666667 | 2.6 |
| GSTT1L | 1.035205427 | 0.002389266 | up | 41.94 | 76.946 |
| DMD | 1.034673871 | 0.002389549 | up | 7.111666667 | 13.638 |
| CYYR1 | -3.157942913 | 0.002716584 | down | 1.25 | 0.126 |
| CKAP2 | 1.382972874 | 0.002749325 | up | 0.273333333 | 0.626 |
| LOC420107 | -1.601343365 | 0.002824262 | down | 1.48 | 0.416 |
| DACH2 | -1.473246301 | 0.002875214 | down | 0.75 | 0.236 |
| MYO1A | 1.327736845 | 0.002941218 | up | 1.606666667 | 3.598 |
| RAD52 | 1.483791369 | 0.002960847 | up | 0.168333333 | 0.424 |
| LOC100857473 | 4.008844846 | 0.003029561 | up | 0.095 | 1.134 |
| LOC121112029 | -1.346027034 | 0.003320352 | down | 1.713333333 | 0.442 |
| LOC112530036 | -2.348137313 | 0.003366584 | down | 1.068333333 | 0.196 |
| GDF9 | -2.464415782 | 0.003443122 | down | 0.37 | 0.058 |
| CAMK1D | -1.425216305 | 0.003582439 | down | 17.74666667 | 5.666 |
| PRF1 | -1.386616848 | 0.00363912 | down | 1.578333333 | 0.476 |
| SLBP | -1.009939035 | 0.003716766 | down | 1.375 | 0.612 |
| PLCD4 | -1.125517291 | 0.003877054 | down | 1.885 | 0.768 |
| CACNA1D | 1.971361776 | 0.004112069 | up | 0.735 | 2.602 |
| LOC107050437 | -2.971959577 | 0.004227213 | down | 0.045 | 0.004 |
| ZP1 | -1.083666766 | 0.004257701 | down | 10.055 | 4.278 |
| LOC427873 | 3.885646852 | 0.004275511 | up | 0.003333333 | 0.026 |
| PBK | 1.267564656 | 0.004531429 | up | 2.02 | 4.236 |
| SHMT1 | 1.282394129 | 0.004691458 | up | 365.5783333 | 796.554 |
| MGAT5B | 1.392492201 | 0.005190367 | up | 2.173333333 | 4.966 |
| CDCA3 | 1.62173442 | 0.005209969 | up | 0.858333333 | 2.314 |
| LOC101748229 | -1.305212924 | 0.005390448 | down | 0.313333333 | 0.124 |
| ETV7 | -1.205829952 | 0.005499877 | down | 3.47 | 1.39 |
| LOC107054090 | -1.144041618 | 0.005514008 | down | 0.305 | 0.128 |
| LOC121108904 | 3.092244443 | 0.005610436 | up | 0.443333333 | 3.398 |
| LOC422895 | -1.061664252 | 0.005673082 | down | 1.253333333 | 0.542 |
| LOC121112410 | -4.705223145 | 0.005973111 | down | 0.448333333 | 0 |
| LOC121112850 | 2.939930974 | 0.006017151 | up | 0.193333333 | 1.342 |
| PCP4L1 | 1.216813775 | 0.006184566 | up | 9.738333333 | 35.296 |
| LOC121111418 | 3.249513267 | 0.006212766 | up | 0.008333333 | 0.074 |
| LOC121107862 | -1.241540808 | 0.006252023 | down | 0.626666667 | 0.236 |
| HIST1H2A4L2 | 1.9886084 | 0.006272586 | up | 3.841666667 | 13.424 |
| LOC427665 | 2.907288009 | 0.006364758 | up | 0.39 | 2.704 |
| TTC9 | 1.45556977 | 0.00646616 | up | 0.643333333 | 1.59 |
| LOC121112480 | 2.400313467 | 0.006496012 | up | 0.075 | 0.35 |
| CALCA | 1.476580097 | 0.006505473 | up | 1.44 | 3.788 |
| LOC107052897 | -1.39010507 | 0.006642036 | down | 0.126666667 | 0.044 |
| NTNG2 | -1.933261409 | 0.007080001 | down | 0.31 | 0.078 |
| MRGPRH | -1.030985217 | 0.007080614 | down | 0.638333333 | 0.28 |
| LOC121109435 | -1.893706463 | 0.00715961 | down | 0.421666667 | 0.094 |
| SYT1 | -3.93906074 | 0.007199213 | down | 0.083333333 | 0 |
| LOC121110105 | -3.021125393 | 0.007219842 | down | 0.558333333 | 0.06 |
| LOC121111345 | 1.115762075 | 0.007839917 | up | 0.653333333 | 1.282 |
| LOC101750420 | 1.071121969 | 0.007943336 | up | 3.456666667 | 9.656 |
| LOC101748203 | -1.636630911 | 0.008430883 | down | 0.521666667 | 0.146 |
| LOC121111035 | -1.193843192 | 0.008474465 | down | 0.438333333 | 0.136 |
| C20orf96 | 3.384365538 | 0.008619943 | up | 0.008333333 | 0.12 |
| GUCY1B4 | 1.607716653 | 0.008626415 | up | 0.046666667 | 0.144 |
| LOC112532338 | -1.637195915 | 0.008719828 | down | 1.716666667 | 0.486 |
| LOC121110893 | -1.709378508 | 0.008792308 | down | 3.81 | 1.138 |
| LOC121110583 | -1.130179663 | 0.008850912 | down | 0.376666667 | 0.154 |
| LOC107056264 | -4.10244636 | 0.008914205 | down | 0.453333333 | 0.018 |
| LOC107054976 | -1.287497645 | 0.009210725 | down | 4.67 | 1.668 |
| LOC121111647 | -1.989550128 | 0.009405373 | down | 0.313333333 | 0.072 |
| LOC121112357 | -3.543701216 | 0.00968908 | down | 4.368333333 | 0.338 |
| LOC107052037 | -2.568198465 | 0.009706616 | down | 0.141666667 | 0.02 |
| AvBD6 | -2.139293005 | 0.009958019 | down | 8.683333333 | 1.84 |
| GRIN2A | -2.310521654 | 0.009975838 | down | 0.083333333 | 0.016 |
| LOC107051670 | -7.775026266 | 0.009982362 | down | 1.446666667 | 0.004 |
| TMEM45L | -1.06971533 | 0.010048363 | down | 4.643333333 | 1.98 |
| PLD4 | -1.141934026 | 0.010321607 | down | 1.148333333 | 0.47 |
| LOC107056413 | 1.281566596 | 0.010330332 | up | 0.865 | 1.838 |
| LOC107055172 | 1.066196896 | 0.010347797 | up | 5 | 7.43 |
| G0S2 | 1.260300585 | 0.010447156 | up | 12.70166667 | 27.374 |
| LOC121109006 | 1.133879053 | 0.0104677 | up | 0.196666667 | 0.392 |
| ZC2HC1B | 2.220479474 | 0.01066422 | up | 0.096666667 | 0.418 |
| LOC101749934 | -1.321476385 | 0.010673222 | down | 0.531666667 | 0.19 |
| LOC112532705 | -1.145511294 | 0.010755033 | down | 0.193333333 | 0.08 |
| LOC417386 | -1.012712258 | 0.010860947 | down | 7.211666667 | 3.214 |
| TMEM156 | -1.035957483 | 0.010988868 | down | 0.941666667 | 0.418 |
| SHISA3 | -3.216348919 | 0.011137169 | down | 0.045 | 0.004 |
| LOC101750540 | -2.770419936 | 0.011457996 | down | 0.038333333 | 0.006 |
| GPC3 | -1.560419933 | 0.012275945 | down | 2.013333333 | 0.478 |
| LOC121110396 | -2.784966953 | 0.012372196 | down | 0.016666667 | 0 |
| LOC121111596 | -1.525491198 | 0.012460874 | down | 0.095 | 0.03 |
| ATP6V0D2 | 2.043114392 | 0.012586131 | up | 0.145 | 0.536 |
| LOC107052745 | -3.727816904 | 0.012593318 | down | 0.261666667 | 0 |
| DNAH12 | -2.752768331 | 0.012606126 | down | 0.02 | 0.002 |
| KNDC1 | -2.757977136 | 0.012841571 | down | 0.07 | 0.01 |
| LOC107054032 | 2.809199914 | 0.013023194 | up | 0.01 | 0.05 |
| LOC121110773 | -1.192630186 | 0.013070338 | down | 0.738333333 | 0.266 |
| LOC112532558 | 1.233367259 | 0.013138603 | up | 0.483333333 | 0.988 |
| CALHM2 | -1.028692907 | 0.013266428 | down | 0.326666667 | 0.142 |
| B4GALNT4 | -1.094451727 | 0.013307327 | down | 0.776666667 | 0.332 |
| LOC121112233 | -1.809961035 | 0.013331531 | down | 0.258333333 | 0.066 |
| RET | -1.064276571 | 0.013569978 | down | 0.428333333 | 0.182 |
| LOC121112225 | -2.083475008 | 0.013608742 | down | 0.281666667 | 0.06 |
| CMPK2 | -1.246652937 | 0.013831526 | down | 47.41333333 | 18.01 |
| ID4 | 1.118916648 | 0.01388552 | up | 1.453333333 | 2.748 |
| MCU | 3.398020353 | 0.013976139 | up | 0.081666667 | 0.754 |
| LOC121111911 | -1.16873812 | 0.014183138 | down | 0.403333333 | 0.174 |
| OC3 | 1.487092692 | 0.014421883 | up | 2.15 | 5.202 |
| LOC112532663 | -1.05406934 | 0.014486929 | down | 0.666666667 | 0.292 |
| RHAG | -1.078062906 | 0.014692013 | down | 1.81 | 0.764 |
| LOC101749901 | 4.467854357 | 0.014867512 | up | 0 | 0.51 |
| EMP1 | 1.179108879 | 0.014913409 | up | 0.243333333 | 0.498 |
| LTC4S | 1.380089694 | 0.01512061 | up | 12.34 | 29.026 |
| PRPH2L | -1.492725659 | 0.015168666 | down | 0.44 | 0.14 |
| SPTBN2 | 1.928806857 | 0.015218443 | up | 0.02 | 0.076 |
| RGS1 | 1.060365329 | 0.01566862 | up | 0.705 | 1.318 |
| DRD5 | -1.605234543 | 0.015897038 | down | 0.35 | 0.104 |
| LOC121107120 | -1.152083584 | 0.016349815 | down | 0.408333333 | 0.164 |
| LOC121109428 | -2.999194136 | 0.016561359 | down | 0.446666667 | 0.048 |
| HRH4 | -2.794111424 | 0.016761384 | down | 0.066666667 | 0.008 |
| LOC121112710 | 3.923635415 | 0.016884323 | up | 0.01 | 0.198 |
| LOC772158 | 3.230985223 | 0.016990742 | up | 0.033333333 | 0.302 |
| LOC121110561 | 2.040329436 | 0.017112055 | up | 0.273333333 | 1.03 |
| LOC121110497 | 2.891822626 | 0.017280126 | up | 0.026666667 | 0.164 |
| LOC107054403 | 1.67622734 | 0.017453342 | up | 0.051666667 | 0.15 |
| SI | 3.27187799 | 0.017619902 | up | 0.001666667 | 0.024 |
| LOC121112289 | -1.078751483 | 0.017712271 | down | 2.203333333 | 0.904 |
| DDAH1 | 1.277446099 | 0.017714866 | up | 0.521666667 | 1.118 |
| CFTR | -1.208989486 | 0.017857605 | down | 0.228333333 | 0.086 |
| CA4 | 1.535989352 | 0.017941271 | up | 9.136666667 | 24.096 |
| LOC101749699 | -2.899114008 | 0.018240714 | down | 0.041666667 | 0.004 |
| LOC112530297 | -1.43582121 | 0.018372824 | down | 15.11166667 | 5.496 |
| LOC121112383 | -3.005107392 | 0.018389648 | down | 0.593333333 | 0.066 |
| CACNB2 | 1.233626129 | 0.018418044 | up | 0.068333333 | 0.138 |
| TGM3 | -1.973271319 | 0.018612854 | down | 0.561666667 | 0.132 |
| LOC121111218 | -2.073496966 | 0.018655619 | down | 0.111666667 | 0.024 |
| LOC121107252 | -1.265044765 | 0.018848252 | down | 2.655 | 0.998 |
| CCR8L | -1.300399359 | 0.01888928 | down | 0.683333333 | 0.25 |
| GIMAP6 | 1.566612929 | 0.019107824 | up | 10.35 | 28.138 |
| LOC121110054 | -1.859749001 | 0.019423885 | down | 4.235 | 0.992 |
| LOC121109706 | 3.483695642 | 0.019446472 | up | 0 | 0.654 |
| ANKS4B | 1.235425437 | 0.019544259 | up | 0.606666667 | 1.288 |
| UNC5A | 2.535486479 | 0.019926264 | up | 0.295 | 1.512 |
| C1QTNF9B | -1.401766175 | 0.020107389 | down | 1.02 | 0.358 |
| CENPE | 1.286645923 | 0.020318626 | up | 0.046666667 | 0.096 |
| DUOXA1L | -4.577389579 | 0.020460113 | down | 0.151666667 | 0 |
| LOC107053686 | -1.116714984 | 0.020841656 | down | 0.3 | 0.114 |
| SLC13A1 | -2.843460492 | 0.020927115 | down | 0.031666667 | 0.004 |
| LOC101751659 | -1.111710466 | 0.021186527 | down | 1.221666667 | 0.504 |
| LOC121110777 | -1.312608162 | 0.021384674 | down | 0.443333333 | 0.202 |
| LOC121111752 | -1.461917118 | 0.021926853 | down | 0.301666667 | 0.094 |
| MADCAM1 | -1.058037899 | 0.022364325 | down | 7.126666667 | 3.068 |
| LOC101750892 | -1.77682908 | 0.022584329 | down | 39.32333333 | 11.354 |
| LOC101752332 | 2.430790655 | 0.022662335 | up | 0.01 | 0.052 |
| LOC121110474 | -2.508031569 | 0.022699232 | down | 0.056666667 | 0.008 |
| TNFSF11 | -1.535246246 | 0.023462407 | down | 0.615 | 0.192 |
| LOC121112197 | -1.594121744 | 0.023655267 | down | 0.075 | 0.022 |
| TMC2 | 2.910042595 | 0.023704138 | up | 0.016666667 | 0.104 |
| KITLG | -1.417146957 | 0.023753947 | down | 0.495 | 0.16 |
| CA14 | -1.620227318 | 0.02406048 | down | 0.535 | 0.156 |
| MUC4 | 1.749466098 | 0.024265821 | up | 0.065 | 0.2 |
| SCN5A | -1.209617635 | 0.024463381 | down | 0.171666667 | 0.068 |
| LOC121107650 | -1.991369919 | 0.024573105 | down | 0.158333333 | 0.034 |
| IFI6 | -1.705989494 | 0.025049378 | down | 671.955 | 187.174 |
| LOC121109556 | -1.648837244 | 0.025304635 | down | 0.128333333 | 0.036 |
| IL1R2 | 1.172117762 | 0.025496733 | up | 1.493333333 | 3.128 |
| ARHGAP22 | -1.051306418 | 0.025566914 | down | 3.688333333 | 2.006 |
| PLA2G10L | -2.183154176 | 0.025919495 | down | 0.63 | 0.13 |
| LOC121110774 | -1.577075766 | 0.026124955 | down | 1.02 | 0.316 |
| SCNN1D | -1.86303777 | 0.026980126 | down | 0.128333333 | 0.036 |
| SNTG1 | 2.977926171 | 0.027302902 | up | 0.005 | 0.032 |
| GLUL | 1.462553138 | 0.027321475 | up | 710.7383333 | 1760.928 |
| LOC101749095 | 1.382429211 | 0.027728822 | up | 0.038333333 | 0.086 |
| LOC112532411 | -1.825625561 | 0.027989647 | down | 0.043333333 | 0.012 |
| LOC121112485 | 1.948563615 | 0.028050261 | up | 0.23 | 0.856 |
| LOC101747703 | -1.520479698 | 0.028155248 | down | 0.091666667 | 0.026 |
| CLCA1 | -3.043523409 | 0.028262756 | down | 0.155 | 0.016 |
| LOC107051724 | -1.813366831 | 0.028456664 | down | 0.121666667 | 0.03 |
| GPT2 | 1.475120015 | 0.028557646 | up | 53.51333333 | 132.748 |
| LOC107049904 | 2.3790147 | 0.028751653 | up | 5.555 | 27.57 |
| NEU2 | 1.664919326 | 0.02879145 | up | 0.465 | 1.336 |
| PGBD5 | -1.52454128 | 0.028820217 | down | 0.06 | 0.018 |
| PAICS | 1.081901699 | 0.028880249 | up | 424.8633333 | 796.622 |
| LOC107054697 | -1.45314129 | 0.029107537 | down | 0.595 | 0.188 |
| NEFL | -1.922136456 | 0.029481275 | down | 0.193333333 | 0.048 |
| HIST1H2A4L1 | 1.069789409 | 0.029770946 | up | 6.4 | 11.8 |
| ACAN | -2.786508868 | 0.030174973 | down | 0.031666667 | 0.004 |
| VAMP2 | 1.61607493 | 0.030204191 | up | 0.018333333 | 0.046 |
| LOC121112495 | 2.074235736 | 0.030325668 | up | 0.048333333 | 0.19 |
| LOC121109452 | 1.704361226 | 0.030330898 | up | 0.031666667 | 0.096 |
| AASDH | 1.255764883 | 0.030357457 | up | 5.678333333 | 11.956 |
| MYPN | 3.084082355 | 0.030448365 | up | 0.006666667 | 0.048 |
| LOC101749682 | -3.368208802 | 0.030910678 | down | 0.061666667 | 0 |
| LOC416655 | -1.211421812 | 0.031283176 | down | 23.87333333 | 9.246 |
| TP63 | -1.466075165 | 0.031347652 | down | 0.271666667 | 0.09 |
| CELSR2 | -2.343397322 | 0.031529449 | down | 0.028333333 | 0.006 |
| AvBD9 | 1.269850371 | 0.031774998 | up | 6264.91 | 13578.208 |
| DGKI | -2.285483888 | 0.032155962 | down | 0.038333333 | 0.008 |
| LAMA1 | -1.505019634 | 0.03227728 | down | 0.051666667 | 0.016 |
| LOC107055678 | -1.13430532 | 0.033037229 | down | 0.23 | 0.094 |
| LOC121111000 | -1.293267359 | 0.033518297 | down | 0.621666667 | 0.226 |
| SIX1 | 1.795163849 | 0.03367791 | up | 0.095 | 0.288 |
| LOC121111727 | 3.273966319 | 0.034037116 | up | 0.008333333 | 0.104 |
| LOC121108763 | 1.798229546 | 0.034152283 | up | 0.98 | 2.886 |
| TUBA8A | -2.800914451 | 0.034188004 | down | 0.093333333 | 0.012 |
| KIF18A | 1.095340833 | 0.034230708 | up | 0.086666667 | 0.166 |
| LOC121112155 | -1.981992704 | 0.034941883 | down | 0.466666667 | 0.102 |
| LOC121112500 | 2.66927034 | 0.035136457 | up | 0.308333333 | 2.054 |
| LOC107055390 | -1.494204054 | 0.035144159 | down | 2.008333333 | 0.64 |
| OAT | 1.042293741 | 0.035529829 | up | 53.32 | 98.544 |
| DUOX1 | -3.517715533 | 0.035711142 | down | 0.065 | 0.004 |
| LRRTM4 | 1.027489898 | 0.035964503 | up | 0.881666667 | 2.644 |
| LAMP3 | -1.471654375 | 0.036774133 | down | 0.91 | 0.298 |
| LOC101749898 | 1.936894635 | 0.036981905 | up | 0.008333333 | 0.026 |
| SSPO | -2.120996244 | 0.037040529 | down | 0.018333333 | 0.004 |
| TMPRSS9 | 1.556297762 | 0.037414357 | up | 0.035 | 0.086 |
| AMY2A | 3.266877784 | 0.03753851 | up | 0.023333333 | 0.2 |
| FBXO47 | -2.401291553 | 0.037614991 | down | 0.106666667 | 0.018 |
| LOC121109571 | -2.661415433 | 0.037622472 | down | 0.031666667 | 0.004 |
| ACMSD | 1.183911729 | 0.037949225 | up | 6.081666667 | 14.746 |
| CCDC170 | -2.902225998 | 0.038758237 | down | 0.041666667 | 0.004 |
| KNTC1 | 1.078263633 | 0.038812361 | up | 0.07 | 0.13 |
| GIMAP7L2 | 2.753368989 | 0.039003272 | up | 0.083333333 | 0.488 |
| LOC121111720 | 3.699594233 | 0.039129334 | up | 0.013333333 | 0.154 |
| EPX | -1.38263033 | 0.039201339 | down | 0.36 | 0.118 |
| LOC107051860 | -1.509438672 | 0.039449845 | down | 0.125 | 0.038 |
| CYP26C1 | -1.594886638 | 0.039719386 | down | 0.108333333 | 0.034 |
| LOC121109903 | -1.384971044 | 0.040093488 | down | 0.31 | 0.108 |
| LOC121112091 | -1.451986884 | 0.040280164 | down | 0.223333333 | 0.076 |
| LOC428702 | 1.244675869 | 0.041490573 | up | 7.29 | 15.33 |
| FAM162B | 1.058709249 | 0.041667798 | up | 0.363333333 | 0.67 |
| SOBP | -1.047307786 | 0.042374669 | down | 0.39 | 0.166 |
| LOC112530000 | 2.892135814 | 0.042501144 | up | 0.008333333 | 0.052 |
| LOC121112254 | 4.051101074 | 0.042847918 | up | 0 | 0.102 |
| LOC425940 | 1.458998927 | 0.042865425 | up | 0.196666667 | 0.482 |
| LOC121109444 | -3.282511427 | 0.042977568 | down | 0.031666667 | 0 |
| METTL21C | -4.083261125 | 0.043070159 | down | 0.121666667 | 0 |
| LOC121110156 | -1.414563111 | 0.043504694 | down | 0.213333333 | 0.07 |
| PYROXD2 | 1.166859078 | 0.044012105 | up | 4.396666667 | 8.892 |
| LOC107052788 | -2.098006487 | 0.04422578 | down | 3.908333333 | 0.67 |
| LOC121106433 | -1.122109158 | 0.044289083 | down | 3.355 | 1.37 |
| LOC121107349 | -1.21850013 | 0.044368436 | down | 0.036666667 | 0.016 |
| SOWAHA | -1.192976394 | 0.044713688 | down | 0.188333333 | 0.074 |
| TRAIP | 1.16484455 | 0.045219991 | up | 0.168333333 | 0.33 |
| SOX17 | 1.144479968 | 0.045815169 | up | 0.3 | 0.58 |
| LOC121110489 | 1.477037069 | 0.045956589 | up | 0.021666667 | 0.05 |
| LOC101750607 | 1.732203997 | 0.046134099 | up | 0.048333333 | 0.14 |
| STAP1 | -1.000300861 | 0.046190022 | down | 0.37 | 0.162 |
| LOC112533251 | 3.65373094 | 0.046204286 | up | 0.001666667 | 0.024 |
| MAB21L3 | 1.118225808 | 0.046361096 | up | 0.186666667 | 0.334 |
| LOC121107324 | -2.339393697 | 0.046733372 | down | 0.041666667 | 0.008 |
| BPIFB3 | 1.886372644 | 0.046809567 | up | 0.063333333 | 0.212 |
| LOC112531715 | -2.819635564 | 0.046867008 | down | 0.04 | 0.002 |
| C26H6ORF222 | -2.477982613 | 0.047274004 | down | 0.098333333 | 0.016 |
| LOC121109411 | 1.65942896 | 0.047435546 | up | 0.018333333 | 0.054 |
| LOC101752184 | -1.343309926 | 0.04765894 | down | 0.751666667 | 0.274 |
| PGR2/3 | -2.020735709 | 0.048148096 | down | 1.216666667 | 0.272 |
| LOC112533195 | 1.647934507 | 0.048305594 | up | 0.398333333 | 1.098 |
| LOC107053924 | 1.828073093 | 0.048598344 | up | 0.065 | 0.186 |
| LOC121110747 | -2.584922099 | 0.048703664 | down | 0.188333333 | 0.028 |
| LOC107053537 | -1.147021615 | 0.048762814 | down | 0.113333333 | 0.046 |
| ADRA2A | 1.054998429 | 0.049259423 | up | 0.255 | 0.472 |
| IL22RA2 | -1.467470121 | 0.049604244 | down | 4.653333333 | 1.518 |
| LOC121112795 | 1.255826997 | 0.049794616 | up | 0.048333333 | 0.098 |
| LOC427816 | -2.459849805 | 0.049868845 | down | 0.126666667 | 0.02 |

**Supplementary Table 9A.** Correlation analysis of FASN with other genes.

| **Gene id** | **Connected gene id** | **Connected gene name** | **Cor** | **Pvalue** | **Qvalue** |
| --- | --- | --- | --- | --- | --- |
| gene-FASN | gene-HSP90AA1 | HSP90AA1 | 0.927273 | 0.000112 | 0.017683 |
| gene-FASN | gene-KIF18A | KIF18A | 0.829284 | 0.003008 | 0.071486 |
| gene-FASN | gene-LAMA1 | LAMA1 | -0.950992 | 0.000024 | 0.006072 |
| gene-FASN | gene-LOC100857706 | LOC100857706 | -0.844989 | 0.002086 | 0.064754 |
| gene-FASN | gene-LOC100858381 | LOC100858381 | -0.830303 | 0.00294 | 0.07086 |
| gene-FASN | gene-LOC101748113 | LOC101748113 | -0.852825 | 0.001712 | 0.060642 |
| gene-FASN | gene-LOC107049798 | LOC107049798 | -0.954412 | 0.000018 | 0.005162 |
| gene-FASN | gene-LOC107050473 | LOC107050473 | -0.878788 | 0.000814 | 0.046072 |
| gene-FASN | gene-LOC107052215 | LOC107052215 | -0.804893 | 0.004973 | 0.084081 |
| gene-FASN | gene-LOC107052745 | LOC107052745 | -0.846926 | 0.001988 | 0.064754 |
| gene-FASN | gene-LOC107052897 | LOC107052897 | -0.914651 | 0.000209 | 0.024243 |
| gene-FASN | gene-LOC107053391 | LOC107053391 | -0.890909 | 0.000542 | 0.039481 |
| gene-FASN | gene-LOC107053924 | LOC107053924 | 0.837022 | 0.002523 | 0.068901 |
| gene-FASN | gene-LOC112530214 | LOC112530214 | -0.842424 | 0.00222 | 0.064754 |
| gene-FASN | gene-LOC112532705 | LOC112532705 | -0.817088 | 0.003902 | 0.077487 |
| gene-FASN | gene-LOC121107650 | LOC121107650 | -0.817088 | 0.003902 | 0.077487 |
| gene-FASN | gene-LOC121109428 | LOC121109428 | -0.80149 | 0.005305 | 0.086324 |
| gene-FASN | gene-LOC121109444 | LOC121109444 | -0.863642 | 0.001279 | 0.055907 |
| gene-FASN | gene-LOC121109571 | LOC121109571 | -0.881131 | 0.000755 | 0.046072 |
| gene-FASN | gene-LOC121110105 | LOC121110105 | -0.86164 | 0.001352 | 0.05701 |
| gene-FASN | gene-LOC121110777 | LOC121110777 | -0.851068 | 0.001791 | 0.061281 |
| gene-FASN | gene-LOC121111218 | LOC121111218 | -0.905237 | 0.000314 | 0.032145 |
| gene-FASN | gene-LOC121112029 | LOC121112029 | -0.963636 | 0.000007 | 0.002213 |
| gene-FASN | gene-LOC121112500 | LOC121112500 | 0.816012 | 0.003989 | 0.078439 |
| gene-FASN | gene-LOC420107 | LOC420107 | -0.830303 | 0.00294 | 0.07086 |
| gene-FASN | gene-LYGL | LYGL | -0.806061 | 0.004862 | 0.082865 |
| gene-FASN | gene-LYZ | LYZ | -0.842424 | 0.00222 | 0.064754 |
| gene-FASN | gene-MID1IP1 | MID1IP1 | -0.818182 | 0.003815 | 0.076684 |
| gene-FASN | gene-MT3 | MT3 | -0.90303 | 0.000344 | 0.032145 |
| gene-FASN | gene-MXD3 | MXD3 | -0.806061 | 0.004862 | 0.082865 |
| gene-FASN | gene-PCP4L1 | PCP4L1 | 0.830303 | 0.00294 | 0.07086 |
| gene-FASN | gene-PRPH2L | PRPH2L | -0.818182 | 0.003815 | 0.076684 |
| gene-FASN | gene-RAD52 | RAD52 | 0.830303 | 0.00294 | 0.07086 |
| gene-FASN | gene-SAMD9L | SAMD9L | -0.842424 | 0.00222 | 0.064754 |
| gene-FASN | gene-TMEM156 | TMEM156 | -0.814593 | 0.004106 | 0.079375 |
| gene-FASN | gene-TP63 | TP63 | -0.887542 | 0.00061 | 0.041299 |

**Supplementary Table 9B.** Interaction analysis of FASN with other proteins.

| **Node1** | **Node2** | **Neighborhood_on_chromosome** | **Gene_fusion** | **Phylogenetic_cooccurrence** | **Homology** | **Coexpression** | **Experimentally_determined_interaction** | **Database_annotated** | **Automated_textmining** | **Combined_score** |
| --- | --- | --- | --- | --- | --- | --- | --- | --- | --- | --- |
| FASN | AASDH | 0.438 | 0 | 0 | 0 | 0.059 | 0 | 0.352 | 0.468 | 0.793 |
| FASN | THRSP | 0 | 0 | 0 | 0 | 0.154 | 0 | 0 | 0.219 | 0.765 |
| FASN | LPIN1 | 0 | 0 | 0 | 0 | 0.048 | 0 | 0 | 0.342 | 0.702 |
| FASN | PCK1 | 0.047 | 0 | 0 | 0 | 0.058 | 0.042 | 0 | 0.162 | 0.672 |
| FASN | PLIN1 | 0 | 0 | 0 | 0 | 0.059 | 0 | 0 | 0.43 | 0.587 |
| FASN | INSIG2 | 0 | 0 | 0 | 0 | 0.059 | 0 | 0 | 0.118 | 0.506 |
| FASN | ACAT2 | 0 | 0 | 0 | 0 | 0.059 | 0.045 | 0.216 | 0.224 | 0.479 |

**Supplementary Table S10A.** GO terms enriched with up-DEGs.

| **Number** | **GO ID** | **Term Type** | **Description** | **Ratio_in_pop** | **Pvalue** |
| --- | --- | --- | --- | --- | --- |
| 42 | GO:0003824 | MF | catalytic activity | 4705/13763 | 0.009212133 |
| 29 | GO:0005515 | MF | protein binding | 3260/13763 | 0.044345061 |
| 19 | GO:0016787 | MF | hydrolase activity | 1858/13763 | 0.03976229 |
| 18 | GO:0036094 | MF | small molecule binding | 1773/13763 | 0.038312009 |
| 15 | GO:0009058 | BP | biosynthetic process | 1080/13763 | 0.004052469 |
| 14 | GO:0009892 | BP | negative regulation of metabolic process | 1038/13763 | 0.00691197 |
| 13 | GO:0044281 | BP | small molecule metabolic process | 751/13763 | 0.000927142 |
| 13 | GO:1901576 | BP | organic substance biosynthetic process | 1037/13763 | 0.022403629 |
| 12 | GO:0044249 | BP | cellular biosynthetic process | 957/13763 | 0.020119011 |
| 11 | GO:0046983 | MF | protein dimerization activity | 541/13763 | 0.000645038 |
| 11 | GO:0031324 | BP | negative regulation of cellular metabolic process | 889/13763 | 0.028641914 |
| 10 | GO:0008092 | MF | cytoskeletal protein binding | 498/13763 | 0.001264036 |
| 10 | GO:0051172 | BP | negative regulation of nitrogen compound metabolic process | 816/13763 | 0.039613809 |
| 9 | GO:0019752 | BP | carboxylic acid metabolic process | 379/13763 | 0.000695001 |
| 9 | GO:0043436 | BP | oxoacid metabolic process | 394/13763 | 0.000915169 |
| 9 | GO:0006082 | BP | organic acid metabolic process | 404/13763 | 0.001091288 |
| 9 | GO:1901362 | BP | organic cyclic compound biosynthetic process | 477/13763 | 0.003379429 |
| 8 | GO:0034654 | BP | nucleobase-containing compound biosynthetic process | 378/13763 | 0.002852912 |
| 8 | GO:0018130 | BP | heterocycle biosynthetic process | 416/13763 | 0.005082159 |
| 8 | GO:0019438 | BP | aromatic compound biosynthetic process | 417/13763 | 0.005154956 |
| 7 | GO:0032993 | CC | protein-DNA complex | 99/13763 | 3.17227E-06 |
| 7 | GO:0046982 | MF | protein heterodimerization activity | 168/13763 | 9.86211E-05 |
| 7 | GO:0003008 | BP | system process | 450/13763 | 0.025003732 |
| 6 | GO:0000786 | CC | nucleosome | 67/13763 | 4.19831E-06 |
| 6 | GO:0044815 | CC | DNA packaging complex | 68/13763 | 4.58114E-06 |
| 6 | GO:0016829 | MF | lyase activity | 125/13763 | 0.000146806 |
| 6 | GO:0050877 | BP | nervous system process | 297/13763 | 0.011875285 |
| 6 | GO:0044255 | BP | cellular lipid metabolic process | 401/13763 | 0.043338331 |
| 5 | GO:1901605 | BP | alpha-amino acid metabolic process | 96/13763 | 0.000368776 |
| 5 | GO:0016054 | BP | organic acid catabolic process | 104/13763 | 0.000533091 |
| 5 | GO:0046395 | BP | carboxylic acid catabolic process | 104/13763 | 0.000533091 |
| 5 | GO:0044282 | BP | small molecule catabolic process | 141/13763 | 0.002083868 |
| 5 | GO:0007600 | BP | sensory perception | 160/13763 | 0.003598325 |
| 5 | GO:0006520 | BP | cellular amino acid metabolic process | 161/13763 | 0.003695268 |
| 5 | GO:0045859 | BP | regulation of protein kinase activity | 255/13763 | 0.023705578 |
| 5 | GO:0007154 | BP | cell communication | 261/13763 | 0.025880536 |
| 5 | GO:0043549 | BP | regulation of kinase activity | 296/13763 | 0.041107008 |
| 5 | GO:0098797 | CC | plasma membrane protein complex | 259/13763 | 0.02514182 |
| 4 | GO:0009064 | BP | glutamine family amino acid metabolic process | 42/13763 | 0.000145085 |
| 4 | GO:0006352 | BP | DNA-templated transcription, initiation | 58/13763 | 0.000508781 |
| 4 | GO:0044242 | BP | cellular lipid catabolic process | 88/13763 | 0.002418128 |
| 4 | GO:0006631 | BP | fatty acid metabolic process | 110/13763 | 0.005383984 |
| 4 | GO:0001664 | MF | G protein-coupled receptor binding | 123/13763 | 0.007953562 |
| 4 | GO:0016042 | BP | lipid catabolic process | 139/13763 | 0.012082587 |
| 4 | GO:1901615 | BP | organic hydroxy compound metabolic process | 162/13763 | 0.020097278 |
| 4 | GO:0045860 | BP | positive regulation of protein kinase activity | 162/13763 | 0.020097278 |
| 4 | GO:0032774 | BP | RNA biosynthetic process | 184/13763 | 0.030258393 |
| 4 | GO:0032787 | BP | monocarboxylic acid metabolic process | 187/13763 | 0.031841139 |
| 4 | GO:0033674 | BP | positive regulation of kinase activity | 198/13763 | 0.038056602 |
| 4 | GO:0005874 | CC | microtubule | 147/13763 | 0.014580845 |
| 4 | GO:0033648 | CC | host intracellular membrane-bounded organelle | 177/13763 | 0.026751039 |
| 4 | GO:0033647 | CC | host intracellular organelle | 177/13763 | 0.026751039 |
| 4 | GO:0042025 | CC | host cell nucleus | 177/13763 | 0.026751039 |
| 4 | GO:0033646 | CC | host intracellular part | 182/13763 | 0.029229827 |
| 4 | GO:0033643 | CC | host cell part | 183/13763 | 0.029741455 |
| 4 | GO:0018995 | CC | host cellular component | 184/13763 | 0.030258393 |
| 4 | GO:0015631 | MF | tubulin binding | 166/13763 | 0.021758248 |
| 3 | GO:0006342 | BP | chromatin silencing | 19/13763 | 0.000227286 |
| 3 | GO:0045814 | BP | negative regulation of gene expression, epigenetic | 26/13763 | 0.000590438 |
| 3 | GO:0016831 | MF | carboxy-lyase activity | 27/13763 | 0.000661183 |
| 3 | GO:0097549 | BP | chromatin organization involved in negative regulation of transcription | 33/13763 | 0.001199644 |
| 3 | GO:0016458 | BP | gene silencing | 34/13763 | 0.00130969 |
| 3 | GO:0043648 | BP | dicarboxylic acid metabolic process | 34/13763 | 0.00130969 |
| 3 | GO:0034401 | BP | chromatin organization involved in regulation of transcription | 35/13763 | 0.00142589 |
| 3 | GO:0016830 | MF | carbon-carbon lyase activity | 37/13763 | 0.00167725 |
| 3 | GO:0009062 | BP | fatty acid catabolic process | 38/13763 | 0.001812652 |
| 3 | GO:0007605 | BP | sensory perception of sound | 46/13763 | 0.003143963 |
| 3 | GO:0050954 | BP | sensory perception of mechanical stimulus | 47/13763 | 0.003342939 |
| 3 | GO:0072329 | BP | monocarboxylic acid catabolic process | 49/13763 | 0.003763647 |
| 3 | GO:0040029 | BP | regulation of gene expression, epigenetic | 60/13763 | 0.006646887 |
| 3 | GO:0051224 | BP | negative regulation of protein transport | 70/13763 | 0.010157911 |
| 3 | GO:1904950 | BP | negative regulation of establishment of protein localization | 71/13763 | 0.010557791 |
| 3 | GO:0004553 | MF | hydrolase activity, hydrolyzing O-glycosyl compounds | 76/13763 | 0.012693423 |
| 3 | GO:0062012 | BP | regulation of small molecule metabolic process | 100/13763 | 0.026183979 |
| 3 | GO:0051656 | BP | establishment of organelle localization | 115/13763 | 0.037365097 |
| 3 | GO:0007018 | BP | microtubule-based movement | 122/13763 | 0.043295481 |
| 3 | GO:0000790 | CC | nuclear chromatin | 115/13763 | 0.037365097 |
| 3 | GO:0019842 | MF | vitamin binding | 84/13763 | 0.016589774 |
| 3 | GO:0016798 | MF | hydrolase activity, acting on glycosyl bonds | 100/13763 | 0.026183979 |
| 3 | GO:0003774 | MF | motor activity | 101/13763 | 0.026863803 |
| 3 | GO:0051015 | MF | actin filament binding | 116/13763 | 0.038184855 |
| 2 | GO:0006522 | BP | alanine metabolic process | 2/13763 | 4.0421E-05 |
| 2 | GO:0009078 | BP | pyruvate family amino acid metabolic process | 2/13763 | 4.0421E-05 |
| 2 | GO:0086007 | MF | voltage-gated calcium channel activity involved in cardiac muscle cell action potential | 2/13763 | 4.0421E-05 |
| 2 | GO:0099635 | MF | voltage-gated calcium channel activity involved in positive regulation of presynaptic cytosolic calcium levels | 3/13763 | 0.000120758 |
| 2 | GO:0006527 | BP | arginine catabolic process | 4/13763 | 0.00024051 |
| 2 | GO:0099626 | MF | voltage-gated calcium channel activity involved in regulation of presynaptic cytosolic calcium levels | 4/13763 | 0.00024051 |
| 2 | GO:1904407 | BP | positive regulation of nitric oxide metabolic process | 5/13763 | 0.000399181 |
| 2 | GO:0045429 | BP | positive regulation of nitric oxide biosynthetic process | 5/13763 | 0.000399181 |
| 2 | GO:1990454 | CC | L-type voltage-gated calcium channel complex | 5/13763 | 0.000399181 |
| 2 | GO:0051379 | MF | epinephrine binding | 5/13763 | 0.000399181 |
| 2 | GO:0008331 | MF | high voltage-gated calcium channel activity | 5/13763 | 0.000399181 |
| 2 | GO:0099511 | MF | voltage-gated calcium channel activity involved in regulation of cytosolic calcium levels | 5/13763 | 0.000399181 |
| 2 | GO:1903428 | BP | positive regulation of reactive oxygen species biosynthetic process | 7/13763 | 0.000831322 |
| 2 | GO:0031690 | MF | adrenergic receptor binding | 7/13763 | 0.000831322 |
| 2 | GO:0006560 | BP | proline metabolic process | 8/13763 | 0.001103821 |
| 2 | GO:0097470 | CC | ribbon synapse | 8/13763 | 0.001103821 |
| 2 | GO:0006089 | BP | lactate metabolic process | 9/13763 | 0.001413303 |
| 2 | GO:1901338 | MF | catecholamine binding | 9/13763 | 0.001413303 |
| 2 | GO:0009065 | BP | glutamine family amino acid catabolic process | 10/13763 | 0.001759292 |
| 2 | GO:0006525 | BP | arginine metabolic process | 10/13763 | 0.001759292 |
| 2 | GO:0042474 | BP | middle ear morphogenesis | 11/13763 | 0.002141321 |
| 2 | GO:0045428 | BP | regulation of nitric oxide biosynthetic process | 11/13763 | 0.002141321 |
| 2 | GO:0006536 | BP | glutamate metabolic process | 14/13763 | 0.003499021 |
| 2 | GO:0006541 | BP | glutamine metabolic process | 14/13763 | 0.003499021 |
| 2 | GO:0001960 | BP | negative regulation of cytokine-mediated signaling pathway | 16/13763 | 0.004575952 |
| 2 | GO:0060348 | BP | bone development | 16/13763 | 0.004575952 |
| 2 | GO:1903426 | BP | regulation of reactive oxygen species biosynthetic process | 16/13763 | 0.004575952 |
| 2 | GO:0060761 | BP | negative regulation of response to cytokine stimulus | 17/13763 | 0.005164614 |
| 2 | GO:0086091 | BP | regulation of heart rate by cardiac conduction | 17/13763 | 0.005164614 |
| 2 | GO:0005891 | CC | voltage-gated calcium channel complex | 18/13763 | 0.005786155 |
| 2 | GO:0008483 | MF | transaminase activity | 16/13763 | 0.004575952 |
| 2 | GO:0016769 | MF | transferase activity, transferring nitrogenous groups | 18/13763 | 0.005786155 |
| 2 | GO:0005903 | CC | brush border | 20/13763 | 0.007126138 |
| 2 | GO:0098862 | CC | cluster of actin-based cell projections | 20/13763 | 0.007126138 |
| 2 | GO:0005245 | MF | voltage-gated calcium channel activity | 20/13763 | 0.007126138 |
| 2 | GO:0070509 | BP | calcium ion import | 21/13763 | 0.007843722 |
| 2 | GO:0061337 | BP | cardiac conduction | 21/13763 | 0.007843722 |
| 2 | GO:0006641 | BP | triglyceride metabolic process | 22/13763 | 0.00859247 |
| 2 | GO:0045833 | BP | negative regulation of lipid metabolic process | 22/13763 | 0.00859247 |
| 2 | GO:2000379 | BP | positive regulation of reactive oxygen species metabolic process | 24/13763 | 0.010181787 |
| 2 | GO:0035637 | BP | multicellular organismal signaling | 26/13763 | 0.011890789 |
| 2 | GO:0005902 | CC | microvillus | 23/13763 | 0.009371963 |
| 2 | GO:0048747 | BP | muscle fiber development | 28/13763 | 0.013716238 |
| 2 | GO:0006639 | BP | acylglycerol metabolic process | 30/13763 | 0.015654956 |
| 2 | GO:0051928 | BP | positive regulation of calcium ion transport | 30/13763 | 0.015654956 |
| 2 | GO:0006638 | BP | neutral lipid metabolic process | 31/13763 | 0.016665815 |
| 2 | GO:0062014 | BP | negative regulation of small molecule metabolic process | 32/13763 | 0.017703828 |
| 2 | GO:0002027 | BP | regulation of heart rate | 33/13763 | 0.018768614 |
| 2 | GO:0071897 | BP | DNA biosynthetic process | 35/13763 | 0.020977003 |
| 2 | GO:0055002 | BP | striated muscle cell development | 37/13763 | 0.023288014 |
| 2 | GO:0007631 | BP | feeding behavior | 38/13763 | 0.024481089 |
| 2 | GO:0055001 | BP | muscle cell development | 39/13763 | 0.025698732 |
| 2 | GO:0016052 | BP | carbohydrate catabolic process | 42/13763 | 0.029495531 |
| 2 | GO:0001959 | BP | regulation of cytokine-mediated signaling pathway | 42/13763 | 0.029495531 |
| 2 | GO:0060759 | BP | regulation of response to cytokine stimulus | 44/13763 | 0.032143145 |
| 2 | GO:0045598 | BP | regulation of fat cell differentiation | 45/13763 | 0.033500852 |
| 2 | GO:0050709 | BP | negative regulation of protein secretion | 45/13763 | 0.033500852 |
| 2 | GO:1901606 | BP | alpha-amino acid catabolic process | 46/13763 | 0.034880711 |
| 2 | GO:1901654 | BP | response to ketone | 46/13763 | 0.034880711 |
| 2 | GO:0002792 | BP | negative regulation of peptide secretion | 48/13763 | 0.03770556 |
| 2 | GO:2000377 | BP | regulation of reactive oxygen species metabolic process | 49/13763 | 0.039149897 |
| 2 | GO:0048545 | BP | response to steroid hormone | 50/13763 | 0.040615078 |
| 2 | GO:0050728 | BP | negative regulation of inflammatory response | 50/13763 | 0.040615078 |
| 2 | GO:0009063 | BP | cellular amino acid catabolic process | 52/13763 | 0.0436067 |
| 2 | GO:0046165 | BP | alcohol biosynthetic process | 52/13763 | 0.0436067 |
| 2 | GO:0043409 | BP | negative regulation of MAPK cascade | 53/13763 | 0.045132513 |
| 2 | GO:0048562 | BP | embryonic organ morphogenesis | 56/13763 | 0.049826253 |
| 2 | GO:0042593 | BP | glucose homeostasis | 56/13763 | 0.049826253 |
| 2 | GO:0034704 | CC | calcium channel complex | 29/13763 | 0.014671634 |
| 2 | GO:0043130 | MF | ubiquitin binding | 31/13763 | 0.016665815 |
| 2 | GO:0016879 | MF | ligase activity, forming carbon-nitrogen bonds | 34/13763 | 0.019859797 |
| 2 | GO:0019205 | MF | nucleobase-containing compound kinase activity | 34/13763 | 0.019859797 |
| 2 | GO:0016538 | MF | cyclin-dependent protein serine/threonine kinase regulator activity | 35/13763 | 0.020977003 |
| 2 | GO:0016836 | MF | hydro-lyase activity | 39/13763 | 0.025698732 |
| 2 | GO:0030170 | MF | pyridoxal phosphate binding | 40/13763 | 0.026940588 |
| 2 | GO:0070279 | MF | vitamin B6 binding | 40/13763 | 0.026940588 |
| 2 | GO:0032182 | MF | ubiquitin-like protein binding | 42/13763 | 0.029495531 |
| 2 | GO:0003777 | MF | microtubule motor activity | 44/13763 | 0.032143145 |
| 2 | GO:0016835 | MF | carbon-oxygen lyase activity | 50/13763 | 0.040615078 |
| 1 | GO:2000819 | BP | regulation of nucleotide-excision repair | 1/13763 | 0.006393955 |
| 1 | GO:0086012 | BP | membrane depolarization during cardiac muscle cell action potential | 1/13763 | 0.006393955 |
| 1 | GO:0072074 | BP | kidney mesenchyme development | 1/13763 | 0.006393955 |
| 1 | GO:0072075 | BP | metanephric mesenchyme development | 1/13763 | 0.006393955 |
| 1 | GO:0010121 | BP | arginine catabolic process to proline via ornithine | 1/13763 | 0.006393955 |
| 1 | GO:1904970 | BP | brush border assembly | 1/13763 | 0.006393955 |
| 1 | GO:0099606 | BP | microtubule plus-end directed mitotic chromosome migration | 1/13763 | 0.006393955 |
| 1 | GO:0099607 | BP | lateral attachment of mitotic spindle microtubules to kinetochore | 1/13763 | 0.006393955 |
| 1 | GO:0071377 | BP | cellular response to glucagon stimulus | 1/13763 | 0.006393955 |
| 1 | GO:0032690 | BP | negative regulation of interleukin-1 alpha production | 1/13763 | 0.006393955 |
| 1 | GO:2000729 | BP | positive regulation of mesenchymal cell proliferation involved in ureter development | 1/13763 | 0.006393955 |
| 1 | GO:0046015 | BP | regulation of transcription by glucose | 1/13763 | 0.006393955 |
| 1 | GO:0019544 | BP | arginine catabolic process to glutamate | 1/13763 | 0.006393955 |
| 1 | GO:0042851 | BP | L-alanine metabolic process | 1/13763 | 0.006393955 |
| 1 | GO:0061055 | BP | myotome development | 1/13763 | 0.006393955 |
| 1 | GO:0072106 | BP | regulation of ureteric bud formation | 1/13763 | 0.006393955 |
| 1 | GO:0072107 | BP | positive regulation of ureteric bud formation | 1/13763 | 0.006393955 |
| 1 | GO:0050712 | BP | negative regulation of interleukin-1 alpha secretion | 1/13763 | 0.006393955 |
| 1 | GO:0036316 | BP | SREBP-SCAP complex retention in endoplasmic reticulum | 1/13763 | 0.006393955 |
| 1 | GO:0019493 | BP | arginine catabolic process to proline | 1/13763 | 0.006393955 |
| 1 | GO:1902949 | BP | positive regulation of tau-protein kinase activity | 1/13763 | 0.006393955 |
| 1 | GO:2000639 | BP | negative regulation of SREBP signaling pathway | 1/13763 | 0.006393955 |
| 1 | GO:2000638 | BP | regulation of SREBP signaling pathway | 1/13763 | 0.006393955 |
| 1 | GO:1905278 | BP | positive regulation of epithelial tube formation | 1/13763 | 0.006393955 |
| 1 | GO:0045002 | BP | double-strand break repair via single-strand annealing | 1/13763 | 0.006393955 |
| 1 | GO:0021610 | BP | facial nerve morphogenesis | 1/13763 | 0.006393955 |
| 1 | GO:0015743 | BP | malate transport | 1/13763 | 0.006393955 |
| 1 | GO:0072199 | BP | regulation of mesenchymal cell proliferation involved in ureter development | 1/13763 | 0.006393955 |
| 1 | GO:0072193 | BP | ureter smooth muscle cell differentiation | 1/13763 | 0.006393955 |
| 1 | GO:0098912 | BP | membrane depolarization during atrial cardiac muscle cell action potential | 1/13763 | 0.006393955 |
| 1 | GO:0030910 | BP | olfactory placode formation | 1/13763 | 0.006393955 |
| 1 | GO:0072095 | BP | regulation of branch elongation involved in ureteric bud branching | 1/13763 | 0.006393955 |
| 1 | GO:0061551 | BP | trigeminal ganglion development | 1/13763 | 0.006393955 |
| 1 | GO:1904106 | BP | protein localization to microvillus | 1/13763 | 0.006393955 |
| 1 | GO:1904640 | BP | response to methionine | 1/13763 | 0.006393955 |
| 1 | GO:0071882 | BP | phospholipase C-activating adrenergic receptor signaling pathway | 1/13763 | 0.006393955 |
| 1 | GO:0071881 | BP | adenylate cyclase-inhibiting adrenergic receptor signaling pathway | 1/13763 | 0.006393955 |
| 1 | GO:0061197 | BP | fungiform papilla morphogenesis | 1/13763 | 0.006393955 |
| 1 | GO:1901301 | BP | regulation of cargo loading into COPII-coated vesicle | 1/13763 | 0.006393955 |
| 1 | GO:1901303 | BP | negative regulation of cargo loading into COPII-coated vesicle | 1/13763 | 0.006393955 |
| 1 | GO:0098683 | CC | cochlear hair cell ribbon synapse | 1/13763 | 0.006393955 |
| 1 | GO:0005971 | CC | ribonucleoside-diphosphate reductase complex | 1/13763 | 0.006393955 |
| 1 | GO:0086056 | MF | voltage-gated calcium channel activity involved in AV node cell action potential | 1/13763 | 0.006393955 |
| 1 | GO:0086059 | MF | voltage-gated calcium channel activity involved SA node cell action potential | 1/13763 | 0.006393955 |
| 1 | GO:0004021 | MF | L-alanine:2-oxoglutarate aminotransferase activity | 1/13763 | 0.006393955 |
| 1 | GO:0031716 | MF | calcitonin receptor binding | 1/13763 | 0.006393955 |
| 1 | GO:0033798 | MF | thyroxine 5-deiodinase activity | 1/13763 | 0.006393955 |
| 1 | GO:0004587 | MF | ornithine-oxo-acid transaminase activity | 1/13763 | 0.006393955 |
| 1 | GO:0047635 | MF | alanine-oxo-acid transaminase activity | 1/13763 | 0.006393955 |
| 1 | GO:0004313 | MF | [acyl-carrier-protein] S-acetyltransferase activity | 1/13763 | 0.006393955 |
| 1 | GO:0004316 | MF | 3-oxoacyl-[acyl-carrier-protein] reductase (NADPH) activity | 1/13763 | 0.006393955 |
| 1 | GO:0004317 | MF | 3-hydroxypalmitoyl-[acyl-carrier-protein] dehydratase activity | 1/13763 | 0.006393955 |
| 1 | GO:0031177 | MF | phosphopantetheine binding | 1/13763 | 0.006393955 |
| 1 | GO:0047451 | MF | 3-hydroxyoctanoyl-[acyl-carrier-protein] dehydratase activity | 1/13763 | 0.006393955 |
| 1 | GO:0004639 | MF | phosphoribosylaminoimidazolesuccinocarboxamide synthase activity | 1/13763 | 0.006393955 |
| 1 | GO:0004638 | MF | phosphoribosylaminoimidazole carboxylase activity | 1/13763 | 0.006393955 |
| 1 | GO:0043727 | MF | 5-amino-4-imidazole carboxylate lyase activity | 1/13763 | 0.006393955 |
| 1 | GO:0047117 | MF | enoyl-[acyl-carrier-protein] reductase (NADPH, A-specific) activity | 1/13763 | 0.006393955 |
| 1 | GO:0102132 | MF | 3-oxo-pimeloyl-[acp] methyl ester reductase activity | 1/13763 | 0.006393955 |
| 1 | GO:0102131 | MF | 3-oxo-glutaryl-[acp] methyl ester reductase activity | 1/13763 | 0.006393955 |
| 1 | GO:0008659 | MF | (3R)-hydroxymyristoyl-[acyl-carrier-protein] dehydratase activity | 1/13763 | 0.006393955 |
| 1 | GO:0016295 | MF | myristoyl-[acyl-carrier-protein] hydrolase activity | 1/13763 | 0.006393955 |
| 1 | GO:0016296 | MF | palmitoyl-[acyl-carrier-protein] hydrolase activity | 1/13763 | 0.006393955 |
| 1 | GO:0016297 | MF | acyl-[acyl-carrier-protein] hydrolase activity | 1/13763 | 0.006393955 |
| 1 | GO:0004797 | MF | thymidine kinase activity | 1/13763 | 0.006393955 |
| 1 | GO:0004320 | MF | oleoyl-[acyl-carrier-protein] hydrolase activity | 1/13763 | 0.006393955 |
| 1 | GO:0004910 | MF | interleukin-1, type II, blocking receptor activity | 1/13763 | 0.006393955 |
| 1 | GO:0008693 | MF | 3-hydroxydecanoyl-[acyl-carrier-protein] dehydratase activity | 1/13763 | 0.006393955 |
| 1 | GO:0019157 | MF | malate oxidase activity | 1/13763 | 0.006393955 |
| 1 | GO:0016403 | MF | dimethylargininase activity | 1/13763 | 0.006393955 |
| 1 | GO:0019171 | MF | 3-hydroxyacyl-[acyl-carrier-protein] dehydratase activity | 1/13763 | 0.006393955 |
| 1 | GO:0031696 | MF | alpha-2C adrenergic receptor binding | 1/13763 | 0.006393955 |
| 1 | GO:0032650 | BP | regulation of interleukin-1 alpha production | 2/13763 | 0.012747489 |
| 1 | GO:0071107 | BP | response to parathyroid hormone | 2/13763 | 0.012747489 |
| 1 | GO:0072071 | BP | kidney interstitial fibroblast differentiation | 2/13763 | 0.012747489 |
| 1 | GO:0071332 | BP | cellular response to fructose stimulus | 2/13763 | 0.012747489 |
| 1 | GO:0000052 | BP | citrulline metabolic process | 2/13763 | 0.012747489 |
| 1 | GO:0008582 | BP | regulation of synaptic growth at neuromuscular junction | 2/13763 | 0.012747489 |
| 1 | GO:0019541 | BP | propionate metabolic process | 2/13763 | 0.012747489 |
| 1 | GO:0019543 | BP | propionate catabolic process | 2/13763 | 0.012747489 |
| 1 | GO:1904428 | BP | negative regulation of tubulin deacetylation | 2/13763 | 0.012747489 |
| 1 | GO:0071599 | BP | otic vesicle development | 2/13763 | 0.012747489 |
| 1 | GO:0046104 | BP | thymidine metabolic process | 2/13763 | 0.012747489 |
| 1 | GO:0045762 | BP | positive regulation of adenylate cyclase activity | 2/13763 | 0.012747489 |
| 1 | GO:0046125 | BP | pyrimidine deoxyribonucleoside metabolic process | 2/13763 | 0.012747489 |
| 1 | GO:0046327 | BP | glycerol biosynthetic process from pyruvate | 2/13763 | 0.012747489 |
| 1 | GO:0007079 | BP | mitotic chromosome movement towards spindle pole | 2/13763 | 0.012747489 |
| 1 | GO:0033762 | BP | response to glucagon | 2/13763 | 0.012747489 |
| 1 | GO:0072513 | BP | positive regulation of secondary heart field cardioblast proliferation | 2/13763 | 0.012747489 |
| 1 | GO:1902947 | BP | regulation of tau-protein kinase activity | 2/13763 | 0.012747489 |
| 1 | GO:0071374 | BP | cellular response to parathyroid hormone stimulus | 2/13763 | 0.012747489 |
| 1 | GO:1905276 | BP | regulation of epithelial tube formation | 2/13763 | 0.012747489 |
| 1 | GO:0060372 | BP | regulation of atrial cardiac muscle cell membrane repolarization | 2/13763 | 0.012747489 |
| 1 | GO:0018991 | BP | oviposition | 2/13763 | 0.012747489 |
| 1 | GO:1905323 | BP | telomerase holoenzyme complex assembly | 2/13763 | 0.012747489 |
| 1 | GO:0050995 | BP | negative regulation of lipid catabolic process | 2/13763 | 0.012747489 |
| 1 | GO:1904985 | BP | negative regulation of quinolinate biosynthetic process | 2/13763 | 0.012747489 |
| 1 | GO:1904984 | BP | regulation of quinolinate biosynthetic process | 2/13763 | 0.012747489 |
| 1 | GO:0009750 | BP | response to fructose | 2/13763 | 0.012747489 |
| 1 | GO:0090735 | BP | DNA repair complex assembly | 2/13763 | 0.012747489 |
| 1 | GO:0050705 | BP | regulation of interleukin-1 alpha secretion | 2/13763 | 0.012747489 |
| 1 | GO:0061550 | BP | cranial ganglion development | 2/13763 | 0.012747489 |
| 1 | GO:0009120 | BP | deoxyribonucleoside metabolic process | 2/13763 | 0.012747489 |
| 1 | GO:0000730 | BP | DNA recombinase assembly | 2/13763 | 0.012747489 |
| 1 | GO:0099059 | CC | integral component of presynaptic active zone membrane | 2/13763 | 0.012747489 |
| 1 | GO:0042587 | CC | glycogen granule | 2/13763 | 0.012747489 |
| 1 | GO:0033181 | CC | plasma membrane proton-transporting V-type ATPase complex | 2/13763 | 0.012747489 |
| 1 | GO:0097427 | CC | microtubule bundle | 2/13763 | 0.012747489 |
| 1 | GO:0046848 | MF | hydroxyapatite binding | 2/13763 | 0.012747489 |
| 1 | GO:0004314 | MF | [acyl-carrier-protein] S-malonyltransferase activity | 2/13763 | 0.012747489 |
| 1 | GO:0004315 | MF | 3-oxoacyl-[acyl-carrier-protein] synthase activity | 2/13763 | 0.012747489 |
| 1 | GO:0016453 | MF | C-acetyltransferase activity | 2/13763 | 0.012747489 |
| 1 | GO:0016418 | MF | S-acetyltransferase activity | 2/13763 | 0.012747489 |
| 1 | GO:0016419 | MF | S-malonyltransferase activity | 2/13763 | 0.012747489 |
| 1 | GO:0004611 | MF | phosphoenolpyruvate carboxykinase activity | 2/13763 | 0.012747489 |
| 1 | GO:0001760 | MF | aminocarboxymuconate-semialdehyde decarboxylase activity | 2/13763 | 0.012747489 |
| 1 | GO:0008906 | MF | inosine kinase activity | 2/13763 | 0.012747489 |
| 1 | GO:0004965 | MF | G protein-coupled GABA receptor activity | 2/13763 | 0.012747489 |
| 1 | GO:0003985 | MF | acetyl-CoA C-acetyltransferase activity | 2/13763 | 0.012747489 |
| 1 | GO:0008147 | MF | structural constituent of bone | 2/13763 | 0.012747489 |
| 1 | GO:0004613 | MF | phosphoenolpyruvate carboxykinase (GTP) activity | 2/13763 | 0.012747489 |
| 1 | GO:0031694 | MF | alpha-2A adrenergic receptor binding | 2/13763 | 0.012747489 |
| 1 | GO:0016420 | MF | malonyltransferase activity | 2/13763 | 0.012747489 |
| 1 | GO:0003266 | BP | regulation of secondary heart field cardioblast proliferation | 3/13763 | 0.019060854 |
| 1 | GO:0061179 | BP | negative regulation of insulin secretion involved in cellular response to glucose stimulus | 3/13763 | 0.019060854 |
| 1 | GO:0030703 | BP | eggshell formation | 3/13763 | 0.019060854 |
| 1 | GO:0030224 | BP | monocyte differentiation | 3/13763 | 0.019060854 |
| 1 | GO:0030223 | BP | neutrophil differentiation | 3/13763 | 0.019060854 |
| 1 | GO:1903131 | BP | mononuclear cell differentiation | 3/13763 | 0.019060854 |
| 1 | GO:0045040 | BP | protein insertion into mitochondrial outer membrane | 3/13763 | 0.019060854 |
| 1 | GO:0035624 | BP | receptor transactivation | 3/13763 | 0.019060854 |
| 1 | GO:0060788 | BP | ectodermal placode formation | 3/13763 | 0.019060854 |
| 1 | GO:0019626 | BP | short-chain fatty acid catabolic process | 3/13763 | 0.019060854 |
| 1 | GO:0006735 | BP | NADH regeneration | 3/13763 | 0.019060854 |
| 1 | GO:0007412 | BP | axon target recognition | 3/13763 | 0.019060854 |
| 1 | GO:0006542 | BP | glutamine biosynthetic process | 3/13763 | 0.019060854 |
| 1 | GO:0051305 | BP | chromosome movement towards spindle pole | 3/13763 | 0.019060854 |
| 1 | GO:0046898 | BP | response to cycloheximide | 3/13763 | 0.019060854 |
| 1 | GO:0007008 | BP | outer mitochondrial membrane organization | 3/13763 | 0.019060854 |
| 1 | GO:0009313 | BP | oligosaccharide catabolic process | 3/13763 | 0.019060854 |
| 1 | GO:0032571 | BP | response to vitamin K | 3/13763 | 0.019060854 |
| 1 | GO:0003264 | BP | regulation of cardioblast proliferation | 4/13763 | 0.025334302 |
| 1 | GO:0072079 | BP | nephron tubule formation | 4/13763 | 0.025334302 |
| 1 | GO:0051044 | BP | positive regulation of membrane protein ectodomain proteolysis | 4/13763 | 0.025334302 |
| 1 | GO:0070741 | BP | response to interleukin-6 | 4/13763 | 0.025334302 |
| 1 | GO:1904396 | BP | regulation of neuromuscular junction development | 4/13763 | 0.025334302 |
| 1 | GO:1901503 | BP | ether biosynthetic process | 4/13763 | 0.025334302 |
| 1 | GO:0006689 | BP | ganglioside catabolic process | 4/13763 | 0.025334302 |
| 1 | GO:0051145 | BP | smooth muscle cell differentiation | 4/13763 | 0.025334302 |
| 1 | GO:0060363 | BP | cranial suture morphogenesis | 4/13763 | 0.025334302 |
| 1 | GO:0006531 | BP | aspartate metabolic process | 4/13763 | 0.025334302 |
| 1 | GO:0010792 | BP | DNA double-strand break processing involved in repair via single-strand annealing | 4/13763 | 0.025334302 |
| 1 | GO:0046504 | BP | glycerol ether biosynthetic process | 4/13763 | 0.025334302 |
| 1 | GO:0060628 | BP | regulation of ER to Golgi vesicle-mediated transport | 4/13763 | 0.025334302 |
| 1 | GO:0010804 | BP | negative regulation of tumor necrosis factor-mediated signaling pathway | 4/13763 | 0.025334302 |
| 1 | GO:0006213 | BP | pyrimidine nucleoside metabolic process | 4/13763 | 0.025334302 |
| 1 | GO:0090557 | BP | establishment of endothelial intestinal barrier | 4/13763 | 0.025334302 |
| 1 | GO:0006114 | BP | glycerol biosynthetic process | 4/13763 | 0.025334302 |
| 1 | GO:0035437 | BP | maintenance of protein localization in endoplasmic reticulum | 4/13763 | 0.025334302 |
| 1 | GO:0007296 | BP | vitellogenesis | 4/13763 | 0.025334302 |
| 1 | GO:0006544 | BP | glycine metabolic process | 4/13763 | 0.025334302 |
| 1 | GO:0008611 | BP | ether lipid biosynthetic process | 4/13763 | 0.025334302 |
| 1 | GO:0045741 | BP | positive regulation of epidermal growth factor-activated receptor activity | 4/13763 | 0.025334302 |
| 1 | GO:0072172 | BP | mesonephric tubule formation | 4/13763 | 0.025334302 |
| 1 | GO:0006103 | BP | 2-oxoglutarate metabolic process | 5/13763 | 0.031568082 |
| 1 | GO:0019401 | BP | alditol biosynthetic process | 5/13763 | 0.031568082 |
| 1 | GO:0019896 | BP | axonal transport of mitochondrion | 5/13763 | 0.031568082 |
| 1 | GO:2000660 | BP | negative regulation of interleukin-1-mediated signaling pathway | 5/13763 | 0.031568082 |
| 1 | GO:0031643 | BP | positive regulation of myelination | 5/13763 | 0.031568082 |
| 1 | GO:0032288 | BP | myelin assembly | 5/13763 | 0.031568082 |
| 1 | GO:0019563 | BP | glycerol catabolic process | 5/13763 | 0.031568082 |
| 1 | GO:0019953 | BP | sexual reproduction | 5/13763 | 0.031568082 |
| 1 | GO:0090103 | BP | cochlea morphogenesis | 5/13763 | 0.031568082 |
| 1 | GO:0032933 | BP | SREBP signaling pathway | 5/13763 | 0.031568082 |
| 1 | GO:0043950 | BP | positive regulation of cAMP-mediated signaling | 5/13763 | 0.031568082 |
| 1 | GO:0046459 | BP | short-chain fatty acid metabolic process | 5/13763 | 0.031568082 |
| 1 | GO:0032688 | BP | negative regulation of interferon-beta production | 5/13763 | 0.031568082 |
| 1 | GO:0006691 | BP | leukotriene metabolic process | 5/13763 | 0.031568082 |
| 1 | GO:0090043 | BP | regulation of tubulin deacetylation | 5/13763 | 0.031568082 |
| 1 | GO:0051451 | BP | myoblast migration | 5/13763 | 0.031568082 |
| 1 | GO:0046485 | BP | ether lipid metabolic process | 5/13763 | 0.031568082 |
| 1 | GO:0006991 | BP | response to sterol depletion | 5/13763 | 0.031568082 |
| 1 | GO:0046785 | BP | microtubule polymerization | 5/13763 | 0.031568082 |
| 1 | GO:0071501 | BP | cellular response to sterol depletion | 5/13763 | 0.031568082 |
| 1 | GO:0086010 | BP | membrane depolarization during action potential | 6/13763 | 0.037762442 |
| 1 | GO:0019405 | BP | alditol catabolic process | 6/13763 | 0.037762442 |
| 1 | GO:0006662 | BP | glycerol ether metabolic process | 6/13763 | 0.037762442 |
| 1 | GO:0019674 | BP | NAD metabolic process | 6/13763 | 0.037762442 |
| 1 | GO:0021602 | BP | cranial nerve morphogenesis | 6/13763 | 0.037762442 |
| 1 | GO:0051418 | BP | microtubule nucleation by microtubule organizing center | 6/13763 | 0.037762442 |
| 1 | GO:0051349 | BP | positive regulation of lyase activity | 6/13763 | 0.037762442 |
| 1 | GO:0006189 | BP | 'de novo' IMP biosynthetic process | 6/13763 | 0.037762442 |
| 1 | GO:0051290 | BP | protein heterotetramerization | 6/13763 | 0.037762442 |
| 1 | GO:0045722 | BP | positive regulation of gluconeogenesis | 6/13763 | 0.037762442 |
| 1 | GO:0061548 | BP | ganglion development | 6/13763 | 0.037762442 |
| 1 | GO:0050711 | BP | negative regulation of interleukin-1 secretion | 6/13763 | 0.037762442 |
| 1 | GO:0015701 | BP | bicarbonate transport | 6/13763 | 0.037762442 |
| 1 | GO:2000659 | BP | regulation of interleukin-1-mediated signaling pathway | 6/13763 | 0.037762442 |
| 1 | GO:0071361 | BP | cellular response to ethanol | 6/13763 | 0.037762442 |
| 1 | GO:1900016 | BP | negative regulation of cytokine production involved in inflammatory response | 6/13763 | 0.037762442 |
| 1 | GO:0015740 | BP | C4-dicarboxylate transport | 6/13763 | 0.037762442 |
| 1 | GO:0045717 | BP | negative regulation of fatty acid biosynthetic process | 6/13763 | 0.037762442 |
| 1 | GO:0007028 | BP | cytoplasm organization | 6/13763 | 0.037762442 |
| 1 | GO:0070862 | BP | negative regulation of protein exit from endoplasmic reticulum | 6/13763 | 0.037762442 |
| 1 | GO:0071353 | BP | cellular response to interleukin-4 | 7/13763 | 0.043917629 |
| 1 | GO:0051315 | BP | attachment of mitotic spindle microtubules to kinetochore | 7/13763 | 0.043917629 |
| 1 | GO:0070365 | BP | hepatocyte differentiation | 7/13763 | 0.043917629 |
| 1 | GO:0060259 | BP | regulation of feeding behavior | 7/13763 | 0.043917629 |
| 1 | GO:0097384 | BP | cellular lipid biosynthetic process | 7/13763 | 0.043917629 |
| 1 | GO:0032097 | BP | positive regulation of response to food | 7/13763 | 0.043917629 |
| 1 | GO:0045922 | BP | negative regulation of fatty acid metabolic process | 7/13763 | 0.043917629 |
| 1 | GO:0006734 | BP | NADH metabolic process | 7/13763 | 0.043917629 |
| 1 | GO:0000003 | BP | reproduction | 7/13763 | 0.043917629 |
| 1 | GO:0032106 | BP | positive regulation of response to extracellular stimulus | 7/13763 | 0.043917629 |
| 1 | GO:0032100 | BP | positive regulation of appetite | 7/13763 | 0.043917629 |
| 1 | GO:0032109 | BP | positive regulation of response to nutrient levels | 7/13763 | 0.043917629 |
| 1 | GO:0070670 | BP | response to interleukin-4 | 7/13763 | 0.043917629 |
| 1 | GO:0008343 | BP | adult feeding behavior | 7/13763 | 0.043917629 |
| 1 | GO:0031281 | BP | positive regulation of cyclase activity | 7/13763 | 0.043917629 |
| 1 | GO:0032060 | BP | bleb assembly | 7/13763 | 0.043917629 |
| 1 | GO:0060037 | BP | pharyngeal system development | 7/13763 | 0.043917629 |
| 1 | GO:0071549 | BP | cellular response to dexamethasone stimulus | 7/13763 | 0.043917629 |
| 1 | GO:0030953 | BP | astral microtubule organization | 7/13763 | 0.043917629 |
| 1 | GO:0005828 | CC | kinetochore microtubule | 3/13763 | 0.019060854 |
| 1 | GO:0032937 | CC | SREBP-SCAP-Insig complex | 3/13763 | 0.019060854 |
| 1 | GO:0044294 | CC | dendritic growth cone | 3/13763 | 0.019060854 |
| 1 | GO:0098945 | CC | intrinsic component of presynaptic active zone membrane | 4/13763 | 0.025334302 |
| 1 | GO:1990023 | CC | mitotic spindle midzone | 4/13763 | 0.025334302 |
| 1 | GO:0000779 | CC | condensed chromosome, centromeric region | 5/13763 | 0.031568082 |
| 1 | GO:0016010 | CC | dystrophin-associated glycoprotein complex | 6/13763 | 0.037762442 |
| 1 | GO:0098684 | CC | photoreceptor ribbon synapse | 6/13763 | 0.037762442 |
| 1 | GO:0031233 | CC | intrinsic component of external side of plasma membrane | 6/13763 | 0.037762442 |
| 1 | GO:0090665 | CC | glycoprotein complex | 6/13763 | 0.037762442 |
| 1 | GO:0120103 | CC | centriolar subdistal appendage | 6/13763 | 0.037762442 |
| 1 | GO:0031362 | CC | anchored component of external side of plasma membrane | 6/13763 | 0.037762442 |
| 1 | GO:0030314 | CC | junctional membrane complex | 7/13763 | 0.043917629 |
| 1 | GO:0016997 | MF | alpha-sialidase activity | 3/13763 | 0.019060854 |
| 1 | GO:0004938 | MF | alpha2-adrenergic receptor activity | 3/13763 | 0.019060854 |
| 1 | GO:0004748 | MF | ribonucleoside-diphosphate reductase activity, thioredoxin disulfide as acceptor | 3/13763 | 0.019060854 |
| 1 | GO:0033142 | MF | progesterone receptor binding | 3/13763 | 0.019060854 |
| 1 | GO:0032795 | MF | heterotrimeric G-protein binding | 3/13763 | 0.019060854 |
| 1 | GO:0061731 | MF | ribonucleoside-diphosphate reductase activity | 3/13763 | 0.019060854 |
| 1 | GO:0016160 | MF | amylase activity | 3/13763 | 0.019060854 |
| 1 | GO:0004356 | MF | glutamate-ammonia ligase activity | 3/13763 | 0.019060854 |
| 1 | GO:0004800 | MF | thyroxine 5'-deiodinase activity | 3/13763 | 0.019060854 |
| 1 | GO:0047134 | MF | protein-disulfide reductase activity | 3/13763 | 0.019060854 |
| 1 | GO:0016211 | MF | ammonia ligase activity | 3/13763 | 0.019060854 |
| 1 | GO:0004035 | MF | alkaline phosphatase activity | 3/13763 | 0.019060854 |
| 1 | GO:0004556 | MF | alpha-amylase activity | 3/13763 | 0.019060854 |
| 1 | GO:0016880 | MF | acid-ammonia (or amide) ligase activity | 3/13763 | 0.019060854 |
| 1 | GO:0103025 | MF | alpha-amylase activity (releasing maltohexaose) | 3/13763 | 0.019060854 |
| 1 | GO:0004308 | MF | exo-alpha-sialidase activity | 3/13763 | 0.019060854 |
| 1 | GO:0030235 | MF | nitric-oxide synthase regulator activity | 3/13763 | 0.019060854 |
| 1 | GO:0016728 | MF | oxidoreductase activity, acting on CH or CH2 groups, disulfide as acceptor | 3/13763 | 0.019060854 |
| 1 | GO:0030911 | MF | TPR domain binding | 3/13763 | 0.019060854 |
| 1 | GO:0019136 | MF | deoxynucleoside kinase activity | 3/13763 | 0.019060854 |
| 1 | GO:0051380 | MF | norepinephrine binding | 3/13763 | 0.019060854 |
| 1 | GO:0016899 | MF | oxidoreductase activity, acting on the CH-OH group of donors, oxygen as acceptor | 4/13763 | 0.025334302 |
| 1 | GO:0019966 | MF | interleukin-1 binding | 4/13763 | 0.025334302 |
| 1 | GO:0050692 | MF | DNA binding domain binding | 4/13763 | 0.025334302 |
| 1 | GO:0043515 | MF | kinetochore binding | 4/13763 | 0.025334302 |
| 1 | GO:0061575 | MF | cyclin-dependent protein serine/threonine kinase activator activity | 4/13763 | 0.025334302 |
| 1 | GO:0008199 | MF | ferric iron binding | 5/13763 | 0.031568082 |
| 1 | GO:0005042 | MF | netrin receptor activity | 5/13763 | 0.031568082 |
| 1 | GO:0048156 | MF | tau protein binding | 5/13763 | 0.031568082 |
| 1 | GO:0003988 | MF | acetyl-CoA C-acyltransferase activity | 5/13763 | 0.031568082 |
| 1 | GO:0000774 | MF | adenyl-nucleotide exchange factor activity | 6/13763 | 0.037762442 |
| 1 | GO:0016813 | MF | hydrolase activity, acting on carbon-nitrogen (but not peptide) bonds, in linear amidines | 6/13763 | 0.037762442 |
| 1 | GO:0004383 | MF | guanylate cyclase activity | 6/13763 | 0.037762442 |
| 1 | GO:0070182 | MF | DNA polymerase binding | 6/13763 | 0.037762442 |
| 1 | GO:0016725 | MF | oxidoreductase activity, acting on CH or CH2 groups | 6/13763 | 0.037762442 |
| 1 | GO:0031996 | MF | thioesterase binding | 7/13763 | 0.043917629 |
| 1 | GO:0004936 | MF | alpha-adrenergic receptor activity | 7/13763 | 0.043917629 |
| 1 | GO:0046790 | MF | virion binding | 7/13763 | 0.043917629 |
| 1 | GO:0004908 | MF | interleukin-1 receptor activity | 7/13763 | 0.043917629 |
| 1 | GO:0097718 | MF | disordered domain specific binding | 7/13763 | 0.043917629 |
| 1 | GO:0005243 | MF | gap junction channel activity | 7/13763 | 0.043917629 |

**Supplementary Table S10B.** GO terms enriched with down-DEGs.

| **Number** | **GO ID** | **Term Type** | **Description** | **Ratio_in_pop** | **Pvalue** |
| --- | --- | --- | --- | --- | --- |
| 53 | GO:0031224 | CC | intrinsic component of membrane | 4275/13763 | 0.001951483 |
| 52 | GO:0016021 | CC | integral component of membrane | 4225/13763 | 0.002662431 |
| 25 | GO:0050896 | BP | response to stimulus | 1828/13763 | 0.019944127 |
| 16 | GO:0006950 | BP | response to stress | 1062/13763 | 0.024586359 |
| 15 | GO:0048869 | BP | cellular developmental process | 979/13763 | 0.029200091 |
| 14 | GO:0004888 | MF | transmembrane signaling receptor activity | 851/13763 | 0.019392403 |
| 14 | GO:0060089 | MF | molecular transducer activity | 965/13763 | 0.047339296 |
| 14 | GO:0038023 | MF | signaling receptor activity | 965/13763 | 0.047339296 |
| 13 | GO:0022857 | MF | transmembrane transporter activity | 755/13763 | 0.014632071 |
| 12 | GO:0005576 | CC | extracellular region | 452/13763 | 0.000536956 |
| 12 | GO:0031226 | CC | intrinsic component of plasma membrane | 471/13763 | 0.000771512 |
| 12 | GO:0005102 | MF | signaling receptor binding | 678/13763 | 0.016649867 |
| 11 | GO:0010647 | BP | positive regulation of cell communication | 628/13763 | 0.023789386 |
| 11 | GO:0023056 | BP | positive regulation of signaling | 634/13763 | 0.024877207 |
| 11 | GO:0005887 | CC | integral component of plasma membrane | 440/13763 | 0.00149318 |
| 11 | GO:0015318 | MF | inorganic molecular entity transmembrane transporter activity | 532/13763 | 0.006342076 |
| 11 | GO:0015075 | MF | ion transmembrane transporter activity | 570/13763 | 0.016747407 |
| 10 | GO:0006952 | BP | defense response | 271/13763 | 0.000119781 |
| 10 | GO:0015267 | MF | channel activity | 316/13763 | 0.000412089 |
| 10 | GO:0022803 | MF | passive transmembrane transporter activity | 316/13763 | 0.000412089 |
| 10 | GO:0005509 | MF | calcium ion binding | 470/13763 | 0.007555603 |
| 9 | GO:0006811 | BP | ion transport | 409/13763 | 0.009035554 |
| 9 | GO:0044093 | BP | positive regulation of molecular function | 519/13763 | 0.046056119 |
| 9 | GO:0031410 | CC | cytoplasmic vesicle | 488/13763 | 0.038977048 |
| 9 | GO:0097708 | CC | intracellular vesicle | 491/13763 | 0.039515292 |
| 9 | GO:0005216 | MF | ion channel activity | 294/13763 | 0.00101804 |
| 8 | GO:0022836 | MF | gated channel activity | 223/13763 | 0.000703455 |
| 8 | GO:0007155 | BP | cell adhesion | 341/13763 | 0.009500554 |
| 8 | GO:0001934 | BP | positive regulation of protein phosphorylation | 344/13763 | 0.009983351 |
| 8 | GO:0022610 | BP | biological adhesion | 344/13763 | 0.009983351 |
| 8 | GO:0042327 | BP | positive regulation of phosphorylation | 381/13763 | 0.017545359 |
| 8 | GO:0043085 | BP | positive regulation of catalytic activity | 402/13763 | 0.023334251 |
| 8 | GO:0045937 | BP | positive regulation of phosphate metabolic process | 406/13763 | 0.024573607 |
| 8 | GO:0010562 | BP | positive regulation of phosphorus metabolic process | 406/13763 | 0.024573607 |
| 8 | GO:0031401 | BP | positive regulation of protein modification process | 433/13763 | 0.034170552 |
| 8 | GO:0022890 | MF | inorganic cation transmembrane transporter activity | 380/13763 | 0.017298544 |
| 8 | GO:0008324 | MF | cation transmembrane transporter activity | 411/13763 | 0.026187113 |
| 7 | GO:0034220 | BP | ion transmembrane transport | 195/13763 | 0.001515367 |
| 7 | GO:0055085 | BP | transmembrane transport | 264/13763 | 0.008000283 |
| 7 | GO:0043207 | BP | response to external biotic stimulus | 284/13763 | 0.011660949 |
| 7 | GO:0009607 | BP | response to biotic stimulus | 294/13763 | 0.013889093 |
| 7 | GO:0048018 | MF | receptor ligand activity | 269/13763 | 0.008822124 |
| 7 | GO:0030546 | MF | signaling receptor activator activity | 273/13763 | 0.009523086 |
| 7 | GO:0030545 | MF | receptor regulator activity | 285/13763 | 0.011871216 |
| 6 | GO:0020037 | MF | heme binding | 114/13763 | 0.000459342 |
| 6 | GO:0046906 | MF | tetrapyrrole binding | 120/13763 | 0.0006033 |
| 6 | GO:0098660 | BP | inorganic ion transmembrane transport | 138/13763 | 0.001251563 |
| 6 | GO:0098542 | BP | defense response to other organism | 154/13763 | 0.002187448 |
| 6 | GO:0006873 | BP | cellular ion homeostasis | 209/13763 | 0.009582072 |
| 6 | GO:0055082 | BP | cellular chemical homeostasis | 232/13763 | 0.015409154 |
| 6 | GO:0055080 | BP | cation homeostasis | 241/13763 | 0.018244521 |
| 6 | GO:0051707 | BP | response to other organism | 244/13763 | 0.019264289 |
| 6 | GO:0098771 | BP | inorganic ion homeostasis | 245/13763 | 0.019612707 |
| 6 | GO:1903530 | BP | regulation of secretion by cell | 260/13763 | 0.025365302 |
| 6 | GO:0050801 | BP | ion homeostasis | 271/13763 | 0.030236906 |
| 6 | GO:0051046 | BP | regulation of secretion | 273/13763 | 0.031184241 |
| 6 | GO:0019725 | BP | cellular homeostasis | 302/13763 | 0.047141908 |
| 6 | GO:0005856 | CC | cytoskeleton | 276/13763 | 0.032641428 |
| 6 | GO:0005261 | MF | cation channel activity | 214/13763 | 0.010685952 |
| 5 | GO:0045860 | BP | positive regulation of protein kinase activity | 162/13763 | 0.013247155 |
| 5 | GO:0043410 | BP | positive regulation of MAPK cascade | 187/13763 | 0.023136615 |
| 5 | GO:0033674 | BP | positive regulation of kinase activity | 198/13763 | 0.028686952 |
| 5 | GO:0030003 | BP | cellular cation homeostasis | 205/13763 | 0.032623919 |
| 5 | GO:0055065 | BP | metal ion homeostasis | 210/13763 | 0.035633397 |
| 5 | GO:0022834 | MF | ligand-gated channel activity | 95/13763 | 0.001384981 |
| 5 | GO:0015276 | MF | ligand-gated ion channel activity | 95/13763 | 0.001384981 |
| 5 | GO:0005126 | MF | cytokine receptor binding | 111/13763 | 0.002746656 |
| 4 | GO:0071902 | BP | positive regulation of protein serine/threonine kinase activity | 106/13763 | 0.01342555 |
| 4 | GO:0098662 | BP | inorganic cation transmembrane transport | 129/13763 | 0.025610184 |
| 4 | GO:0098655 | BP | cation transmembrane transport | 136/13763 | 0.030306287 |
| 4 | GO:0050708 | BP | regulation of protein secretion | 147/13763 | 0.038652312 |
| 4 | GO:0072503 | BP | cellular divalent inorganic cation homeostasis | 148/13763 | 0.039470089 |
| 4 | GO:0072507 | BP | divalent inorganic cation homeostasis | 155/13763 | 0.045470917 |
| 4 | GO:0030594 | MF | neurotransmitter receptor activity | 65/13763 | 0.002396175 |
| 4 | GO:0015103 | MF | inorganic anion transmembrane transporter activity | 68/13763 | 0.002825666 |
| 4 | GO:0008083 | MF | growth factor activity | 95/13763 | 0.009246689 |
| 3 | GO:0050829 | BP | defense response to Gram-negative bacterium | 29/13763 | 0.001954576 |
| 3 | GO:0015698 | BP | inorganic anion transport | 31/13763 | 0.002374431 |
| 3 | GO:0051607 | BP | defense response to virus | 39/13763 | 0.004591495 |
| 3 | GO:0048871 | BP | multicellular organismal homeostasis | 49/13763 | 0.008695988 |
| 3 | GO:0098656 | BP | anion transmembrane transport | 52/13763 | 0.010237939 |
| 3 | GO:0002685 | BP | regulation of leukocyte migration | 62/13763 | 0.016465121 |
| 3 | GO:0042742 | BP | defense response to bacterium | 69/13763 | 0.021842791 |
| 3 | GO:0050714 | BP | positive regulation of protein secretion | 72/13763 | 0.024407142 |
| 3 | GO:0009615 | BP | response to virus | 79/13763 | 0.030995865 |
| 3 | GO:0043406 | BP | positive regulation of MAP kinase activity | 81/13763 | 0.03303319 |
| 3 | GO:0002793 | BP | positive regulation of peptide secretion | 83/13763 | 0.035138816 |
| 3 | GO:0045211 | CC | postsynaptic membrane | 63/13763 | 0.017181565 |
| 3 | GO:0097060 | CC | synaptic membrane | 85/13763 | 0.037312374 |
| 3 | GO:0005254 | MF | chloride channel activity | 40/13763 | 0.004932822 |
| 3 | GO:0005544 | MF | calcium-dependent phospholipid binding | 42/13763 | 0.005660435 |
| 3 | GO:0098960 | MF | postsynaptic neurotransmitter receptor activity | 43/13763 | 0.006047064 |
| 3 | GO:0015108 | MF | chloride transmembrane transporter activity | 49/13763 | 0.008695988 |
| 3 | GO:0005253 | MF | anion channel activity | 49/13763 | 0.008695988 |
| 3 | GO:0005230 | MF | extracellular ligand-gated ion channel activity | 60/13763 | 0.015083767 |
| 3 | GO:0099094 | MF | ligand-gated cation channel activity | 61/13763 | 0.015765868 |
| 3 | GO:0005539 | MF | glycosaminoglycan binding | 76/13763 | 0.028068616 |
| 3 | GO:0015081 | MF | sodium ion transmembrane transporter activity | 81/13763 | 0.03303319 |
| 3 | GO:0005125 | MF | cytokine activity | 92/13763 | 0.045448324 |
| 2 | GO:0016998 | BP | cell wall macromolecule catabolic process | 3/13763 | 0.00022115 |
| 2 | GO:0044036 | BP | cell wall macromolecule metabolic process | 3/13763 | 0.00022115 |
| 2 | GO:0003796 | MF | lysozyme activity | 3/13763 | 0.00022115 |
| 2 | GO:0061783 | MF | peptidoglycan muralytic activity | 4/13763 | 0.000439795 |
| 2 | GO:0000270 | BP | peptidoglycan metabolic process | 5/13763 | 0.000728842 |
| 2 | GO:0009253 | BP | peptidoglycan catabolic process | 5/13763 | 0.000728842 |
| 2 | GO:0035774 | BP | positive regulation of insulin secretion involved in cellular response to glucose stimulus | 11/13763 | 0.003874698 |
| 2 | GO:1902476 | BP | chloride transmembrane transport | 11/13763 | 0.003874698 |
| 2 | GO:0006027 | BP | glycosaminoglycan catabolic process | 11/13763 | 0.003874698 |
| 2 | GO:0098661 | BP | inorganic anion transmembrane transport | 12/13763 | 0.00462344 |
| 2 | GO:0006026 | BP | aminoglycan catabolic process | 12/13763 | 0.00462344 |
| 2 | GO:0019835 | BP | cytolysis | 12/13763 | 0.00462344 |
| 2 | GO:0007159 | BP | leukocyte cell-cell adhesion | 15/13763 | 0.007232005 |
| 2 | GO:0072163 | BP | mesonephric epithelium development | 17/13763 | 0.009262227 |
| 2 | GO:0061178 | BP | regulation of insulin secretion involved in cellular response to glucose stimulus | 21/13763 | 0.013983951 |
| 2 | GO:0072073 | BP | kidney epithelium development | 22/13763 | 0.015296261 |
| 2 | GO:0097553 | BP | calcium ion transmembrane import into cytosol | 23/13763 | 0.016659357 |
| 2 | GO:0001755 | BP | neural crest cell migration | 24/13763 | 0.018072276 |
| 2 | GO:0046889 | BP | positive regulation of lipid biosynthetic process | 24/13763 | 0.018072276 |
| 2 | GO:0006821 | BP | chloride transport | 24/13763 | 0.018072276 |
| 2 | GO:0008361 | BP | regulation of cell size | 24/13763 | 0.018072276 |
| 2 | GO:0032024 | BP | positive regulation of insulin secretion | 24/13763 | 0.018072276 |
| 2 | GO:0050830 | BP | defense response to Gram-positive bacterium | 26/13763 | 0.021043806 |
| 2 | GO:0060402 | BP | calcium ion transport into cytosol | 28/13763 | 0.024203413 |
| 2 | GO:0034605 | BP | cellular response to heat | 28/13763 | 0.024203413 |
| 2 | GO:0045104 | BP | intermediate filament cytoskeleton organization | 29/13763 | 0.025851475 |
| 2 | GO:0045103 | BP | intermediate filament-based process | 29/13763 | 0.025851475 |
| 2 | GO:0018958 | BP | phenol-containing compound metabolic process | 29/13763 | 0.025851475 |
| 2 | GO:0008306 | BP | associative learning | 31/13763 | 0.029279678 |
| 2 | GO:0099003 | BP | vesicle-mediated transport in synapse | 31/13763 | 0.029279678 |
| 2 | GO:0060401 | BP | cytosolic calcium ion transport | 33/13763 | 0.032878204 |
| 2 | GO:0071346 | BP | cellular response to interferon-gamma | 35/13763 | 0.036640278 |
| 2 | GO:0046683 | BP | response to organophosphorus | 35/13763 | 0.036640278 |
| 2 | GO:0002705 | BP | positive regulation of leukocyte mediated immunity | 36/13763 | 0.038580577 |
| 2 | GO:0002687 | BP | positive regulation of leukocyte migration | 36/13763 | 0.038580577 |
| 2 | GO:0034341 | BP | response to interferon-gamma | 37/13763 | 0.040559303 |
| 2 | GO:0007613 | BP | memory | 37/13763 | 0.040559303 |
| 2 | GO:0030203 | BP | glycosaminoglycan metabolic process | 37/13763 | 0.040559303 |
| 2 | GO:0090277 | BP | positive regulation of peptide hormone secretion | 37/13763 | 0.040559303 |
| 2 | GO:0014074 | BP | response to purine-containing compound | 39/13763 | 0.044628856 |
| 2 | GO:0062013 | BP | positive regulation of small molecule metabolic process | 40/13763 | 0.046718121 |
| 2 | GO:0006022 | BP | aminoglycan metabolic process | 41/13763 | 0.048842687 |
| 2 | GO:0015630 | CC | microtubule cytoskeleton | 36/13763 | 0.038580577 |
| 2 | GO:0061778 | MF | intracellular chloride channel activity | 8/13763 | 0.002006341 |
| 2 | GO:0005229 | MF | intracellular calcium activated chloride channel activity | 8/13763 | 0.002006341 |
| 2 | GO:0035381 | MF | ATP-gated ion channel activity | 9/13763 | 0.002565018 |
| 2 | GO:0016646 | MF | oxidoreductase activity, acting on the CH-NH group of donors, NAD or NADP as acceptor | 12/13763 | 0.00462344 |
| 2 | GO:0004970 | MF | ionotropic glutamate receptor activity | 14/13763 | 0.006303181 |
| 2 | GO:0016645 | MF | oxidoreductase activity, acting on the CH-NH group of donors | 17/13763 | 0.009262227 |
| 2 | GO:0008066 | MF | glutamate receptor activity | 19/13763 | 0.011515594 |
| 2 | GO:0022839 | MF | ion gated channel activity | 20/13763 | 0.0127234 |
| 2 | GO:0008373 | MF | sialyltransferase activity | 22/13763 | 0.015296261 |
| 2 | GO:0048020 | MF | CCR chemokine receptor binding | 23/13763 | 0.016659357 |
| 2 | GO:0030971 | MF | receptor tyrosine kinase binding | 24/13763 | 0.018072276 |
| 2 | GO:0005272 | MF | sodium channel activity | 26/13763 | 0.021043806 |
| 2 | GO:0004601 | MF | peroxidase activity | 26/13763 | 0.021043806 |
| 2 | GO:0016684 | MF | oxidoreductase activity, acting on peroxide as acceptor | 29/13763 | 0.025851475 |
| 2 | GO:1990782 | MF | protein tyrosine kinase binding | 33/13763 | 0.032878204 |
| 2 | GO:0042379 | MF | chemokine receptor binding | 33/13763 | 0.032878204 |
| 2 | GO:0022835 | MF | transmitter-gated channel activity | 39/13763 | 0.044628856 |
| 2 | GO:0022824 | MF | transmitter-gated ion channel activity | 39/13763 | 0.044628856 |
| 1 | GO:1903980 | BP | positive regulation of microglial cell activation | 1/13763 | 0.008646371 |
| 1 | GO:1901509 | BP | regulation of endothelial tube morphogenesis | 1/13763 | 0.008646371 |
| 1 | GO:0033141 | BP | positive regulation of peptidyl-serine phosphorylation of STAT protein | 1/13763 | 0.008646371 |
| 1 | GO:0098038 | BP | non-replicative transposition, DNA-mediated | 1/13763 | 0.008646371 |
| 1 | GO:0008272 | BP | sulfate transport | 1/13763 | 0.008646371 |
| 1 | GO:0150078 | BP | positive regulation of neuroinflammatory response | 1/13763 | 0.008646371 |
| 1 | GO:1901529 | BP | positive regulation of anion channel activity | 1/13763 | 0.008646371 |
| 1 | GO:1903969 | BP | regulation of response to macrophage colony-stimulating factor | 1/13763 | 0.008646371 |
| 1 | GO:0032196 | BP | transposition | 1/13763 | 0.008646371 |
| 1 | GO:1902159 | BP | regulation of cyclic nucleotide-gated ion channel activity | 1/13763 | 0.008646371 |
| 1 | GO:0070668 | BP | positive regulation of mast cell proliferation | 1/13763 | 0.008646371 |
| 1 | GO:1904151 | BP | positive regulation of microglial cell mediated cytotoxicity | 1/13763 | 0.008646371 |
| 1 | GO:0048855 | BP | adenohypophysis morphogenesis | 1/13763 | 0.008646371 |
| 1 | GO:0033026 | BP | negative regulation of mast cell apoptotic process | 1/13763 | 0.008646371 |
| 1 | GO:0033025 | BP | regulation of mast cell apoptotic process | 1/13763 | 0.008646371 |
| 1 | GO:0060681 | BP | branch elongation involved in ureteric bud branching | 1/13763 | 0.008646371 |
| 1 | GO:0006842 | BP | tricarboxylic acid transport | 1/13763 | 0.008646371 |
| 1 | GO:1904139 | BP | regulation of microglial cell migration | 1/13763 | 0.008646371 |
| 1 | GO:1902226 | BP | regulation of macrophage colony-stimulating factor signaling pathway | 1/13763 | 0.008646371 |
| 1 | GO:1902227 | BP | negative regulation of macrophage colony-stimulating factor signaling pathway | 1/13763 | 0.008646371 |
| 1 | GO:0035377 | BP | transepithelial water transport | 1/13763 | 0.008646371 |
| 1 | GO:1902943 | BP | positive regulation of voltage-gated chloride channel activity | 1/13763 | 0.008646371 |
| 1 | GO:1902941 | BP | regulation of voltage-gated chloride channel activity | 1/13763 | 0.008646371 |
| 1 | GO:0061146 | BP | Peyer's patch morphogenesis | 1/13763 | 0.008646371 |
| 1 | GO:0006313 | BP | transposition, DNA-mediated | 1/13763 | 0.008646371 |
| 1 | GO:0015746 | BP | citrate transport | 1/13763 | 0.008646371 |
| 1 | GO:0042045 | BP | epithelial fluid transport | 1/13763 | 0.008646371 |
| 1 | GO:1902161 | BP | positive regulation of cyclic nucleotide-gated ion channel activity | 1/13763 | 0.008646371 |
| 1 | GO:1903970 | BP | negative regulation of response to macrophage colony-stimulating factor | 1/13763 | 0.008646371 |
| 1 | GO:1903973 | BP | negative regulation of cellular response to macrophage colony-stimulating factor stimulus | 1/13763 | 0.008646371 |
| 1 | GO:1903972 | BP | regulation of cellular response to macrophage colony-stimulating factor stimulus | 1/13763 | 0.008646371 |
| 1 | GO:1903797 | BP | positive regulation of inorganic anion transmembrane transport | 1/13763 | 0.008646371 |
| 1 | GO:1904140 | BP | negative regulation of microglial cell migration | 1/13763 | 0.008646371 |
| 1 | GO:0070378 | BP | positive regulation of ERK5 cascade | 1/13763 | 0.008646371 |
| 1 | GO:1904149 | BP | regulation of microglial cell mediated cytotoxicity | 1/13763 | 0.008646371 |
| 1 | GO:0070376 | BP | regulation of ERK5 cascade | 1/13763 | 0.008646371 |
| 1 | GO:0060100 | BP | positive regulation of phagocytosis, engulfment | 2/13763 | 0.017218605 |
| 1 | GO:0050665 | BP | hydrogen peroxide biosynthetic process | 2/13763 | 0.017218605 |
| 1 | GO:0034157 | BP | positive regulation of toll-like receptor 7 signaling pathway | 2/13763 | 0.017218605 |
| 1 | GO:0034155 | BP | regulation of toll-like receptor 7 signaling pathway | 2/13763 | 0.017218605 |
| 1 | GO:2000870 | BP | regulation of progesterone secretion | 2/13763 | 0.017218605 |
| 1 | GO:0035799 | BP | ureter maturation | 2/13763 | 0.017218605 |
| 1 | GO:0051351 | BP | positive regulation of ligase activity | 2/13763 | 0.017218605 |
| 1 | GO:1900028 | BP | negative regulation of ruffle assembly | 2/13763 | 0.017218605 |
| 1 | GO:1903961 | BP | positive regulation of anion transmembrane transport | 2/13763 | 0.017218605 |
| 1 | GO:0070666 | BP | regulation of mast cell proliferation | 2/13763 | 0.017218605 |
| 1 | GO:0035860 | BP | glial cell-derived neurotrophic factor receptor signaling pathway | 2/13763 | 0.017218605 |
| 1 | GO:0097323 | BP | B cell adhesion | 2/13763 | 0.017218605 |
| 1 | GO:0030431 | BP | sleep | 2/13763 | 0.017218605 |
| 1 | GO:0016185 | BP | synaptic vesicle budding from presynaptic endocytic zone membrane | 2/13763 | 0.017218605 |
| 1 | GO:0034165 | BP | positive regulation of toll-like receptor 9 signaling pathway | 2/13763 | 0.017218605 |
| 1 | GO:0051672 | BP | catabolism by organism of cell wall peptidoglycan in other organism | 2/13763 | 0.017218605 |
| 1 | GO:1903997 | BP | positive regulation of non-membrane spanning protein tyrosine kinase activity | 2/13763 | 0.017218605 |
| 1 | GO:1903995 | BP | regulation of non-membrane spanning protein tyrosine kinase activity | 2/13763 | 0.017218605 |
| 1 | GO:0061687 | BP | detoxification of inorganic compound | 2/13763 | 0.017218605 |
| 1 | GO:0033139 | BP | regulation of peptidyl-serine phosphorylation of STAT protein | 2/13763 | 0.017218605 |
| 1 | GO:2000553 | BP | positive regulation of T-helper 2 cell cytokine production | 2/13763 | 0.017218605 |
| 1 | GO:2000551 | BP | regulation of T-helper 2 cell cytokine production | 2/13763 | 0.017218605 |
| 1 | GO:0051340 | BP | regulation of ligase activity | 2/13763 | 0.017218605 |
| 1 | GO:0044040 | BP | multi-organism carbohydrate metabolic process | 2/13763 | 0.017218605 |
| 1 | GO:0044041 | BP | multi-organism carbohydrate catabolic process | 2/13763 | 0.017218605 |
| 1 | GO:1903978 | BP | regulation of microglial cell activation | 2/13763 | 0.017218605 |
| 1 | GO:1903976 | BP | negative regulation of glial cell migration | 2/13763 | 0.017218605 |
| 1 | GO:0060099 | BP | regulation of phagocytosis, engulfment | 2/13763 | 0.017218605 |
| 1 | GO:1905155 | BP | positive regulation of membrane invagination | 2/13763 | 0.017218605 |
| 1 | GO:1905153 | BP | regulation of membrane invagination | 2/13763 | 0.017218605 |
| 1 | GO:0033058 | BP | directional locomotion | 2/13763 | 0.017218605 |
| 1 | GO:0010273 | BP | detoxification of copper ion | 2/13763 | 0.017218605 |
| 1 | GO:0009189 | BP | deoxyribonucleoside diphosphate biosynthetic process | 3/13763 | 0.025717332 |
| 1 | GO:0046072 | BP | dTDP metabolic process | 3/13763 | 0.025717332 |
| 1 | GO:0048799 | BP | animal organ maturation | 3/13763 | 0.025717332 |
| 1 | GO:1902035 | BP | positive regulation of hematopoietic stem cell proliferation | 3/13763 | 0.025717332 |
| 1 | GO:0090205 | BP | positive regulation of cholesterol metabolic process | 3/13763 | 0.025717332 |
| 1 | GO:0009186 | BP | deoxyribonucleoside diphosphate metabolic process | 3/13763 | 0.025717332 |
| 1 | GO:0072216 | BP | positive regulation of metanephros development | 3/13763 | 0.025717332 |
| 1 | GO:1905522 | BP | negative regulation of macrophage migration | 3/13763 | 0.025717332 |
| 1 | GO:0072488 | BP | ammonium transmembrane transport | 3/13763 | 0.025717332 |
| 1 | GO:0140239 | BP | postsynaptic endocytosis | 3/13763 | 0.025717332 |
| 1 | GO:0044035 | BP | multi-organism catabolic process | 3/13763 | 0.025717332 |
| 1 | GO:0044033 | BP | multi-organism metabolic process | 3/13763 | 0.025717332 |
| 1 | GO:0072298 | BP | regulation of metanephric glomerulus development | 3/13763 | 0.025717332 |
| 1 | GO:0050942 | BP | positive regulation of pigment cell differentiation | 3/13763 | 0.025717332 |
| 1 | GO:0009196 | BP | pyrimidine deoxyribonucleoside diphosphate metabolic process | 3/13763 | 0.025717332 |
| 1 | GO:0006233 | BP | dTDP biosynthetic process | 3/13763 | 0.025717332 |
| 1 | GO:0010760 | BP | negative regulation of macrophage chemotaxis | 3/13763 | 0.025717332 |
| 1 | GO:0009139 | BP | pyrimidine nucleoside diphosphate biosynthetic process | 3/13763 | 0.025717332 |
| 1 | GO:0034163 | BP | regulation of toll-like receptor 9 signaling pathway | 3/13763 | 0.025717332 |
| 1 | GO:0060602 | BP | branch elongation of an epithelium | 3/13763 | 0.025717332 |
| 1 | GO:0050803 | BP | regulation of synapse structure or activity | 3/13763 | 0.025717332 |
| 1 | GO:0098884 | BP | postsynaptic neurotransmitter receptor internalization | 3/13763 | 0.025717332 |
| 1 | GO:0034123 | BP | positive regulation of toll-like receptor signaling pathway | 3/13763 | 0.025717332 |
| 1 | GO:0048087 | BP | positive regulation of developmental pigmentation | 3/13763 | 0.025717332 |
| 1 | GO:0070142 | BP | synaptic vesicle budding | 3/13763 | 0.025717332 |
| 1 | GO:0045542 | BP | positive regulation of cholesterol biosynthetic process | 3/13763 | 0.025717332 |
| 1 | GO:0009197 | BP | pyrimidine deoxyribonucleoside diphosphate biosynthetic process | 3/13763 | 0.025717332 |
| 1 | GO:0035234 | BP | ectopic germ cell programmed cell death | 3/13763 | 0.025717332 |
| 1 | GO:0055129 | BP | L-proline biosynthetic process | 3/13763 | 0.025717332 |
| 1 | GO:0042044 | BP | fluid transport | 3/13763 | 0.025717332 |
| 1 | GO:1903795 | BP | regulation of inorganic anion transmembrane transport | 3/13763 | 0.025717332 |
| 1 | GO:0006561 | BP | proline biosynthetic process | 3/13763 | 0.025717332 |
| 1 | GO:0072300 | BP | positive regulation of metanephric glomerulus development | 3/13763 | 0.025717332 |
| 1 | GO:0010359 | BP | regulation of anion channel activity | 3/13763 | 0.025717332 |
| 1 | GO:0048265 | BP | response to pain | 3/13763 | 0.025717332 |
| 1 | GO:0006833 | BP | water transport | 3/13763 | 0.025717332 |
| 1 | GO:0002830 | BP | positive regulation of type 2 immune response | 3/13763 | 0.025717332 |
| 1 | GO:1990743 | BP | protein sialylation | 3/13763 | 0.025717332 |
| 1 | GO:0106120 | BP | positive regulation of sterol biosynthetic process | 3/13763 | 0.025717332 |
| 1 | GO:0045636 | BP | positive regulation of melanocyte differentiation | 3/13763 | 0.025717332 |
| 1 | GO:0033693 | BP | neurofilament bundle assembly | 3/13763 | 0.025717332 |
| 1 | GO:0015696 | BP | ammonium transport | 3/13763 | 0.025717332 |
| 1 | GO:0098703 | BP | calcium ion import across plasma membrane | 4/13763 | 0.034143178 |
| 1 | GO:0050932 | BP | regulation of pigment cell differentiation | 4/13763 | 0.034143178 |
| 1 | GO:2000544 | BP | regulation of endothelial cell chemotaxis to fibroblast growth factor | 4/13763 | 0.034143178 |
| 1 | GO:0045852 | BP | pH elevation | 4/13763 | 0.034143178 |
| 1 | GO:0090193 | BP | positive regulation of glomerulus development | 4/13763 | 0.034143178 |
| 1 | GO:0001555 | BP | oocyte growth | 4/13763 | 0.034143178 |
| 1 | GO:0071280 | BP | cellular response to copper ion | 4/13763 | 0.034143178 |
| 1 | GO:0015669 | BP | gas transport | 4/13763 | 0.034143178 |
| 1 | GO:1904847 | BP | regulation of cell chemotaxis to fibroblast growth factor | 4/13763 | 0.034143178 |
| 1 | GO:0006398 | BP | mRNA 3'-end processing by stem-loop binding and cleavage | 4/13763 | 0.034143178 |
| 1 | GO:0009138 | BP | pyrimidine nucleoside diphosphate metabolic process | 4/13763 | 0.034143178 |
| 1 | GO:0050861 | BP | positive regulation of B cell receptor signaling pathway | 4/13763 | 0.034143178 |
| 1 | GO:1903975 | BP | regulation of glial cell migration | 4/13763 | 0.034143178 |
| 1 | GO:0051454 | BP | intracellular pH elevation | 4/13763 | 0.034143178 |
| 1 | GO:0010623 | BP | programmed cell death involved in cell development | 4/13763 | 0.034143178 |
| 1 | GO:0033033 | BP | negative regulation of myeloid cell apoptotic process | 4/13763 | 0.034143178 |
| 1 | GO:0003347 | BP | epicardial cell to mesenchymal cell transition | 4/13763 | 0.034143178 |
| 1 | GO:0045793 | BP | positive regulation of cell size | 4/13763 | 0.034143178 |
| 1 | GO:0045792 | BP | negative regulation of cell size | 4/13763 | 0.034143178 |
| 1 | GO:0045634 | BP | regulation of melanocyte differentiation | 4/13763 | 0.034143178 |
| 1 | GO:0015670 | BP | carbon dioxide transport | 4/13763 | 0.034143178 |
| 1 | GO:0007197 | BP | adenylate cyclase-inhibiting G protein-coupled acetylcholine receptor signaling pathway | 5/13763 | 0.042496762 |
| 1 | GO:0001964 | BP | startle response | 5/13763 | 0.042496762 |
| 1 | GO:0035999 | BP | tetrahydrofolate interconversion | 5/13763 | 0.042496762 |
| 1 | GO:0019896 | BP | axonal transport of mitochondrion | 5/13763 | 0.042496762 |
| 1 | GO:0050855 | BP | regulation of B cell receptor signaling pathway | 5/13763 | 0.042496762 |
| 1 | GO:0043032 | BP | positive regulation of macrophage activation | 5/13763 | 0.042496762 |
| 1 | GO:2001225 | BP | regulation of chloride transport | 5/13763 | 0.042496762 |
| 1 | GO:0120033 | BP | negative regulation of plasma membrane bounded cell projection assembly | 5/13763 | 0.042496762 |
| 1 | GO:0150077 | BP | regulation of neuroinflammatory response | 5/13763 | 0.042496762 |
| 1 | GO:0061756 | BP | leukocyte adhesion to vascular endothelial cell | 5/13763 | 0.042496762 |
| 1 | GO:0019755 | BP | one-carbon compound transport | 5/13763 | 0.042496762 |
| 1 | GO:0045723 | BP | positive regulation of fatty acid biosynthetic process | 5/13763 | 0.042496762 |
| 1 | GO:0050901 | BP | leukocyte tethering or rolling | 5/13763 | 0.042496762 |
| 1 | GO:0030241 | BP | skeletal muscle myosin thick filament assembly | 5/13763 | 0.042496762 |
| 1 | GO:0106118 | BP | regulation of sterol biosynthetic process | 5/13763 | 0.042496762 |
| 1 | GO:1901160 | BP | primary amino compound metabolic process | 5/13763 | 0.042496762 |
| 1 | GO:0045540 | BP | regulation of cholesterol biosynthetic process | 5/13763 | 0.042496762 |
| 1 | GO:0072348 | BP | sulfur compound transport | 5/13763 | 0.042496762 |
| 1 | GO:0001661 | BP | conditioned taste aversion | 5/13763 | 0.042496762 |
| 1 | GO:0071688 | BP | striated muscle myosin thick filament assembly | 5/13763 | 0.042496762 |
| 1 | GO:0090192 | BP | regulation of glomerulus development | 5/13763 | 0.042496762 |
| 1 | GO:0060586 | BP | multicellular organismal iron ion homeostasis | 5/13763 | 0.042496762 |
| 1 | GO:0099590 | BP | neurotransmitter receptor internalization | 5/13763 | 0.042496762 |
| 1 | GO:0045110 | BP | intermediate filament bundle assembly | 5/13763 | 0.042496762 |
| 1 | GO:0071294 | BP | cellular response to zinc ion | 5/13763 | 0.042496762 |
| 1 | GO:0042428 | BP | serotonin metabolic process | 5/13763 | 0.042496762 |
| 1 | GO:0098966 | CC | perisynaptic extracellular matrix | 2/13763 | 0.017218605 |
| 1 | GO:0042584 | CC | chromaffin granule membrane | 2/13763 | 0.017218605 |
| 1 | GO:0098844 | CC | postsynaptic endocytic zone membrane | 2/13763 | 0.017218605 |
| 1 | GO:0044327 | CC | dendritic spine head | 2/13763 | 0.017218605 |
| 1 | GO:0071204 | CC | histone pre-mRNA 3'end processing complex | 4/13763 | 0.034143178 |
| 1 | GO:0017146 | CC | NMDA selective glutamate receptor complex | 4/13763 | 0.034143178 |
| 1 | GO:0030123 | CC | AP-3 adaptor complex | 5/13763 | 0.042496762 |
| 1 | GO:0044194 | CC | cytolytic granule | 5/13763 | 0.042496762 |
| 1 | GO:0005883 | CC | neurofilament | 5/13763 | 0.042496762 |
| 1 | GO:0071207 | MF | histone pre-mRNA stem-loop binding | 1/13763 | 0.008646371 |
| 1 | GO:0015382 | MF | sodium:sulfate symporter activity | 1/13763 | 0.008646371 |
| 1 | GO:0042301 | MF | phosphate ion binding | 1/13763 | 0.008646371 |
| 1 | GO:0106138 | MF | Sec61 translocon complex binding | 1/13763 | 0.008646371 |
| 1 | GO:0004803 | MF | transposase activity | 1/13763 | 0.008646371 |
| 1 | GO:0030298 | MF | receptor signaling protein tyrosine kinase activator activity | 1/13763 | 0.008646371 |
| 1 | GO:0005260 | MF | intracellularly ATP-gated chloride channel activity | 1/13763 | 0.008646371 |
| 1 | GO:0015111 | MF | iodide transmembrane transporter activity | 1/13763 | 0.008646371 |
| 1 | GO:0099142 | MF | intracellularly ATP-gated ion channel activity | 1/13763 | 0.008646371 |
| 1 | GO:0015141 | MF | succinate transmembrane transporter activity | 2/13763 | 0.017218605 |
| 1 | GO:0015142 | MF | tricarboxylic acid transmembrane transporter activity | 2/13763 | 0.017218605 |
| 1 | GO:0098640 | MF | integrin binding involved in cell-matrix adhesion | 2/13763 | 0.017218605 |
| 1 | GO:0005068 | MF | transmembrane receptor protein tyrosine kinase adaptor activity | 2/13763 | 0.017218605 |
| 1 | GO:0045145 | MF | single-stranded DNA 5'-3' exodeoxyribonuclease activity | 2/13763 | 0.017218605 |
| 1 | GO:0005173 | MF | stem cell factor receptor binding | 2/13763 | 0.017218605 |
| 1 | GO:0052851 | MF | ferric-chelate reductase (NADPH) activity | 2/13763 | 0.017218605 |
| 1 | GO:0005157 | MF | macrophage colony-stimulating factor receptor binding | 2/13763 | 0.017218605 |
| 1 | GO:0008823 | MF | cupric reductase activity | 2/13763 | 0.017218605 |
| 1 | GO:0015373 | MF | anion:sodium symporter activity | 2/13763 | 0.017218605 |
| 1 | GO:0015137 | MF | citrate transmembrane transporter activity | 2/13763 | 0.017218605 |
| 1 | GO:0004972 | MF | NMDA glutamate receptor activity | 3/13763 | 0.025717332 |
| 1 | GO:0017153 | MF | sodium:dicarboxylate symporter activity | 3/13763 | 0.025717332 |
| 1 | GO:0019869 | MF | chloride channel inhibitor activity | 3/13763 | 0.025717332 |
| 1 | GO:0043426 | MF | MRF binding | 3/13763 | 0.025717332 |
| 1 | GO:0004477 | MF | methenyltetrahydrofolate cyclohydrolase activity | 3/13763 | 0.025717332 |
| 1 | GO:0016231 | MF | beta-N-acetylglucosaminidase activity | 3/13763 | 0.025717332 |
| 1 | GO:0004735 | MF | pyrroline-5-carboxylate reductase activity | 3/13763 | 0.025717332 |
| 1 | GO:0001665 | MF | alpha-N-acetylgalactosaminide alpha-2,6-sialyltransferase activity | 3/13763 | 0.025717332 |
| 1 | GO:0098634 | MF | cell-matrix adhesion mediator activity | 3/13763 | 0.025717332 |
| 1 | GO:0016174 | MF | NAD(P)H oxidase (H(2)O(2)-forming activity | 3/13763 | 0.025717332 |
| 1 | GO:0004798 | MF | thymidylate kinase activity | 3/13763 | 0.025717332 |
| 1 | GO:0032184 | MF | SUMO polymer binding | 3/13763 | 0.025717332 |
| 1 | GO:0022849 | MF | glutamate-gated calcium ion channel activity | 3/13763 | 0.025717332 |
| 1 | GO:0004486 | MF | methylenetetrahydrofolate dehydrogenase [NAD(P)+] activity | 3/13763 | 0.025717332 |
| 1 | GO:0004487 | MF | methylenetetrahydrofolate dehydrogenase (NAD+) activity | 3/13763 | 0.025717332 |
| 1 | GO:0004488 | MF | methylenetetrahydrofolate dehydrogenase (NADP+) activity | 3/13763 | 0.025717332 |
| 1 | GO:0004563 | MF | beta-N-acetylhexosaminidase activity | 4/13763 | 0.034143178 |
| 1 | GO:0008297 | MF | single-stranded DNA exodeoxyribonuclease activity | 4/13763 | 0.034143178 |
| 1 | GO:0030159 | MF | signaling receptor complex adaptor activity | 4/13763 | 0.034143178 |
| 1 | GO:0015296 | MF | anion:cation symporter activity | 5/13763 | 0.042496762 |
| 1 | GO:0017081 | MF | chloride channel regulator activity | 5/13763 | 0.042496762 |
| 1 | GO:0019238 | MF | cyclohydrolase activity | 5/13763 | 0.042496762 |
